# Long non-coding RNAs as a biomarker for homologous recombination deficiency and parp inhibitor sensitivity in high-grade serous ovarian cancers

**DOI:** 10.1038/s42003-025-08836-9

**Published:** 2025-10-01

**Authors:** Kai Doberstein, Johannes Panther, Eric Hahnen, Lisa Richters, Philip C. Schouten, Philipp Harter, Florian Heitz, Stefan Kommoss, Sebastian Berlit, Benjamin Tuschy, Marc Sütterlin, Frederik Marmé

**Affiliations:** 1 Department of Obstetrics and Gynecology, Medical Faculty Mannheim of the Heidelberg University, University Medical Centre Mannheim, Mannheim, Germany; 2 Mannheim Institute for Innate Immunoscience, Medical Faculty Mannheim of the Heidelberg University, Mannheim, Germany; 3 Center for Familial Breast and Ovarian Cancer and Center for Integrated Oncology (CIO), Cologne, Faculty of Medicine and University Hospital Cologne, Cologne, Germany; 4 Department of histopathology, Addenbrooke’s Hospital, Cambridge University Hospitals NHS Foundation Trust, cambridge, UK; 5 Department of Gynecology and Gynecologic Oncology, Evang. Kliniken Essen-Mitte, Essen, Germany; 6 Department for Gynecology with the Center for Oncologic Surgery Charité Campus Virchow-Klinikum, Charité – Universitätsmedizin Berlin, corporate member of Freie Universität Berlin, Humboldt-Universität zu Berlin, and Berlin Institute of Health, Berlin, Germany; 7 Department of Women’s Health, Tübingen University Hospital, Tübingen, Germany; 8 Department of Obstetrics and Gynecology, Diakonie Klinikum, Schwäbisch Hall, Germany; 9 AGO Study Group, Wiesbaden, Germany; 10 DKFZ-Hector Cancer Institute at University Medical Center Mannheim, Mannheim, Germany

**Keywords:** Ovarian cancer, Tumour biomarkers, Long non-coding RNAs

## Abstract

Homologous recombination deficiency (HRD) and sensitivity to PARP inhibitors are key determinants of therapeutic response in high-grade serous ovarian cancer (HGSC), yet predictive biomarkers beyond BRCA1/2 mutations or genomic HRD scores remain inadequate. Here, we investigate the potential of long non-coding RNAs (lncRNAs) as predictive markers of HRD and PARP inhibitor response. We identify a panel of lncRNAs that stratifies HGSC tumors by HRD status and drug sensitivity. Among these, ENSG00000272172.1 is significantly upregulated in HRD-positive tumors and is detectable in both formalin-fixed tissue and plasma, supporting its use as a minimally invasive biomarker. Functional analyses reveal that this lncRNA contributes to genome stability by modulating replication dynamics. These findings highlight a previously unrecognized role for lncRNAs in the HRD phenotype and suggest translational potential for ENSG00000272172.1 in guiding clinical decision-making.

## Abstract (web summary)

Long non-coding RNAs are identified as non-invasive biomarkers predicting homologous recombination deficiency and PARP inhibitor sensitivity in high-grade serous ovarian cancer, enabling improved precision oncology approaches.

## Introduction

HGSC of the fallopian tube, ovary, and peritoneum is a particularly lethal form of gynecologic malignancy^1–3^. However, targeted therapies, particularly PARPi, have led to significant progress in recent years, with improvements in survival, especially in patients with *BRCA1* or *BRCA2* mutations^4,5^. Pre-clinical and clinical studies have further demonstrated that many patients beyond these *BRCA1/2* mutation carriers may also benefit from PARPi therapy, due to inactivation of the homologous recombination (HR) DNA repair pathway^6–8^. According to the Cancer Genome Atlas (TCGA) consortium, approximately half of HGSCs harbor defects in the HR pathway^9^.

Due to synergistic mechanisms, PARPi are substantially more effective in cells that harbor deficiencies in HR^10^. Tumor cells that lack HR are dependent on alternative mechanisms to repair DNA double strand breaks (DSBs). PARPi traps the PARP1 molecule on the chromatin, creating a DSB at the replication fork during the S phase of the cell cycle that can only be accurately repaired by the HR pathway. The dependence on alternative, error-prone DSB repair pathways leads to the accumulation of intolerable levels of genomic instability, which will finally lead to cell death^7,10,11^.

Predicting PARPi response in HGSC patients remains a significant challenge for clinicians, as the various mechanisms that can cause HRD make it difficult to predict response. Currently, two genome-based approaches are used clinically: (1) targeted capture-based next-generation sequencing (NGS) and (2) measurement of a so called HRD-score, which is based on the quantification of genomic alterations that are characteristic of genomic instability caused by defects in the HR pathway^12^. These methods have limitations, such that they require high-quality tumor tissue and may not cover all causes of HRD. In addition, these “genomic scars” provide information only about past genomic events and may not reflect the current HRD status.

LncRNAs are a class of RNA molecules longer than 500 nucleotides that are generally not thought to encode functional proteins. However, emerging evidence suggests that some lncRNAs may have coding potential, although the extent and biological significance of this remain to be fully defined. Recent studies have revealed that lncRNAs play important roles in a wide range of biological processes through their ability to interact with DNA, RNA, and proteins^13^. Multiple lncRNAs have been shown to regulate gene expression and specific pathways, such as telomere stability, chromosome remodeling, and DNA repair^14^. These interactions can influence mRNA stability through the modulation of mRNA and microRNA (miRNA) levels^15,16^.

When compared to mRNAs, lncRNAs have been characterized by their low evolutionary conservation, low expression levels, and high tissue specificity^17,18^. However, mounting evidence has shown that lncRNAs are dysregulated in multiple cancer types, including HGSC, and play important roles in processes such as metastasis, immune infiltration, invasion, and tumor growth^19–22^.

LncRNAs can also be protected from degradation through interactions with RNA-binding proteins or localization in exosomes, making them potential biomarkers that can be detected in body fluids^23–30^. A number of prognostic lncRNAs, such as PCA3, H19, HOTAIR, and MALAT1, have been developed and investigated for multiple cancer types, such as prostate cancer and gastric cancer^31–33^.

In this study, we aimed to identify a panel of lncRNAs that could predict HRD and PARPi response in HGSC. We identified 29 lncRNAs that could predict the HR-score and PARPi7-score in ovarian cancer tissue of the TCGA dataset. Amongst these lncRNAs, ENSG00000272172.1 strongly correlated with *BRCA* mutation status and HRD-score in RNA isolated from tissue as well as plasma samples. Mechanistic analysis revealed that ENSG00000272172.1 stabilizes replication procession by reducing replication speed. Our results suggest that lncRNAs may have potential as a blood-based biomarker to predict PARPi response in HGSC patients.

## Results

### Selecting lncRNAs to predict HRD and PARP inhibitor sensitivity

The ability of cells to repair double-strand breaks via HR is an intrinsic mechanism that plays a central role in determining the response to PARPi.

In this study, we investigated whether a panel of lncRNAs plays a role in HRD and is able to predict therapeutic response to PARPi in HGSCs. To identify a robust panel of relevant lncRNAs, we utilized different pre-calculated genomic scores, such as the genomic-based HRD-score, as well as expression-based scores such as the PARPi7 and eCARD, which were developed to predict drug responses based on mRNA expression data^34,35^. Additionally, *CCNE1* amplification status was chosen as a measure, as its amplification is mutually exclusive with *BRCA1* mutation, and *CCNE1* amplified tumors are less sensitive towards PARP inhibitors^36^. We first compared the similarity between different scores of the pan-cancer, breast-cancer, and ovarian-cancer TCGA dataset using Pearson correlation (Supplementary Fig. 1a–f). In the TCGA ovarian cancer dataset, we observed that *CCNE1* expression levels and *CCNE1* copy number status correlated negatively with ploidy-score, aneuploidy-scores, and genome doubling, while both showed a negative correlation to the PARPi7-score and different HRD-scores (Supplementary Fig. 1a). No correlation was found between *CCNE1* and the chemosensitivity score eCARD. Furthermore, when analyzing how the HRD-score correlates with other scores, we observed a positive correlation to LOH and the PARPi7-score, and a negative correlation to aneuploidy score (Supplementary Fig. 1a).

Our results from the ovarian cancer dataset did not reproduce in the pan-cancer or breast cancer datasets (Supplementary Fig. 1c–f). Due to the fact that HGSC is mainly driven by copy number changes rather than recurrent mutations, except the nearly ubiquitous *TP53* mutation and recurrent *BRCA1/2* mutations, it is genetically more homogenous compared to other cancer types. With approximately half the cancers harboring HRD, the ovarian cancer dataset of the TCGA is also more balanced and suitable to compare the similarity between different scores, such as HRD or the PARPi7-score^9^.

**Fig. 1:**
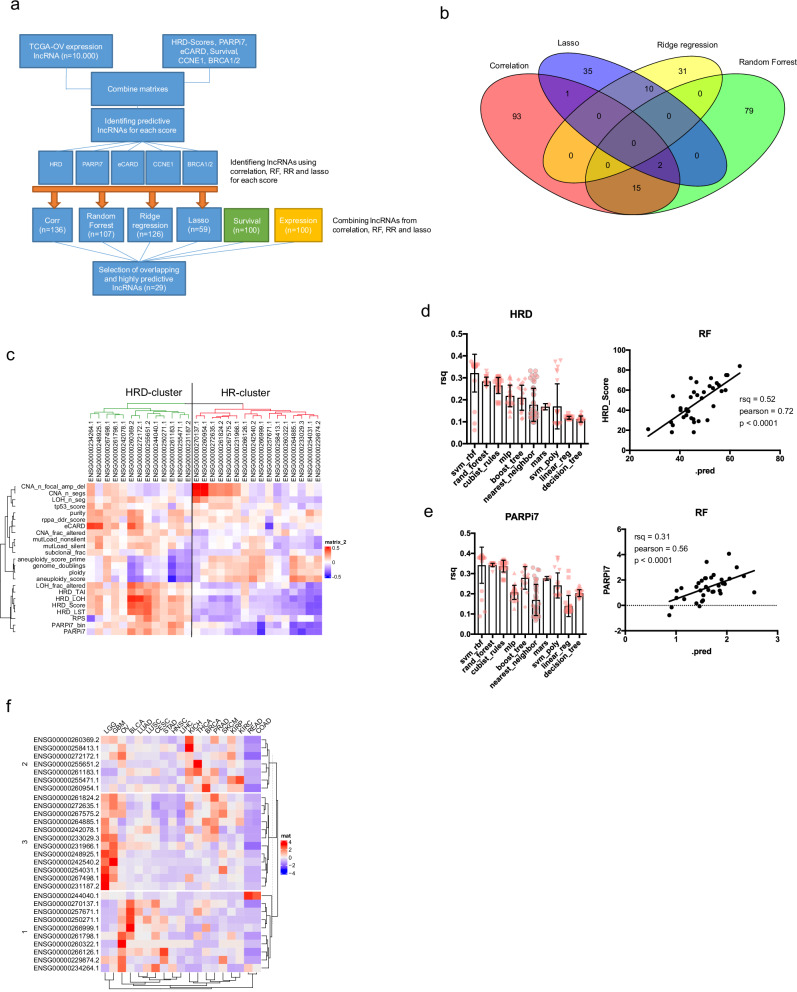
Selecting lncRNAs to predict HRD and PARP inhibitor sensitivity. **a** Flowchart of the lncRNA selection process based on their predictive value for HRD-score, PARPi7-score, eCARD-score, *CCNE1* amplification status or *BRCA1/2* mutation status using multiple feature extraction methods. **b** Venn diagram showing overlap of different feature extraction methods. **c** Heatmap of pearson correlation matrix of different scores against the expression of 29 lncRNAs. **d** Left: Prediction of HRD-scores using different algorithms with 10-time cross validation on the TCGA ovarian cancer dataset. Right: Correlation of predicted HRD-score and measured HRD-score on the test dataset using the random forest algorithm. **e** Left: Prediction of PARPi7-scores using different algorithms with 10-time cross validation on the TCGA ovarian cancer dataset. Right: Correlation of predicted-PARPi7 and measured PARPi7 on the test dataset using the random forest algorithm. **f** Heat map of the average expression of 29 lncRNA in different cancer types of the pan-cancer dataset of the TCGA data.

We aimed to identify a panel of lncRNAs with predictive value for CCNE1 amplification status, BRCA1/2 mutation status, eCARD-, HRD-, and PARPi7-scores (Fig. 1a). To do so, we employed multiple feature extraction techniques, including correlation coefficient analysis, random forest, lasso regression, and ridge regression algorithms. These methods were applied to over 10,000 annotated lncRNAs (Fig. 1b). For each feature extraction method, a list of predictive lncRNAs was generated based on their relevance to the specific predictive value (Supplementary Table 1).

In order to improve the robustness and reliability of the selected panel, we manually selected 29 lncRNAs that were identified by more than one method or that appeared in more than one feature extraction process. This approach ensured that the lncRNAs included were consistently predicted across multiple methods. Additionally, we incorporated lncRNAs that demonstrated high tumor expression or that were correlated with significant changes in patient survival, which provided further validation of their potential relevance. The final panel of 29 lncRNAs thus represents those most consistently predictive of the different outcomes, offering robustness in their potential as biomarkers for further investigation. To evaluate the potential protein-coding capacity of the 29 selected lncRNAs, we analyzed them using five independent computational tools; PRIDE reprocessing 2.0, Lee translation initiation site predictions, PhyloCSF, CPAT, and Bazzini small ORFs. Only three lncRNAs showed evidence of coding potential in one of the five tools, supporting the general classification of these transcripts as non-coding (Supplementary Table 2).

### Predicting HRD- and PARPi7-score

When comparing the correlation of the 29 lncRNAs to different scores, we observed that about half of the lncRNAs clustered with a positive correlation to HRD scores and the other half with a negative correlation (Fig. 1c). Since no external dataset with compatible lncRNA expression and HRD/PARPi7-score annotations was available for independent validation, we tested the predictive utility of our lncRNA panel within the TCGA ovarian cancer dataset. To ensure methodological rigor and reduce overfitting, we implemented a structured Train/Validation/Test splitting strategy: the dataset was divided into training (60%), validation (20%), and test (20%) sets using stratified sampling based on HRD/PARPi7-scores to ensure balanced outcome distribution. The model was trained and tuned exclusively on the training set using 10-fold cross-validation, and final performance metrics were evaluated on the held-out test set, which remained untouched during model development. Using cross-validation with various machine learning algorithms, we found that support vector machine (SVM) and random forest (RF) models yielded the best performance in predicting the HRD score (Fig. 1d). The RF model achieved an *R*² of 0.52 and a Pearson correlation of 0.72 on the testing dataset (Fig. 1d). Using a permutation feature importance analysis further confirmed that the lncRNAs that correlated highest with HRD score, such as ENSG0000272172.1, were also among the top predictors (Supplementary Fig. 2a). We next assessed the ability of the 29 lncRNAs to predict the continuous or binary PARPi7 score^35,37^. Using ten-time cross-validation, the RF, Cubist, and SVM models showed strong predictive performance in the ovarian cancer dataset (Fig. 1e). The RF model achieved an *R*² of 0.31 and a Pearson correlation of 0.56 in the testing dataset (Fig. 1e). For binary classification of PARPi7 sensitivity, XGBoost (XGB) and generalized linear model (GLM) performed best on the test dataset (Supplementary Fig. 2b,c). Feature importance analysis from the GLM model identified ENSG00000264885.1 and ENSG00000261183.1 as the top predictive lncRNAs (Supplementary Fig. 2d).

These data indicate that lncRNAs from this panel could be useful for predicting HRD status or PARP inhibitor sensitivity.

Since lncRNAs have been shown to have a unique tissue expression, we asked if the expression pattern of those 29 lncRNAs was unique in different cancer types^14^. When comparing the average expression of the 29 lncRNAs in 19 cancer types of the pan-cancer dataset of the TCGA, we observed a unique pattern of expression for multiple cancer types (Fig. 1f).

### Distinct prognostic profiles revealed by lncRNA expression

When analyzing the correlation between lncRNA expression and Reverse-Phase Protein Array (RPPA) data in the ovarian cancer TCGA data we observe a similar clustering of the lncRNAs as compared with the HR and HRD cluster in Fig. 1c (Fig. 1c and Supplementary Fig. 3a). Notably, ENSG00000272172.1 and ENSG00000255651.2 that display the highest correlation to HRD- and PARPi-scores cluster together in the RPPA analysis.

**Fig. 2:**
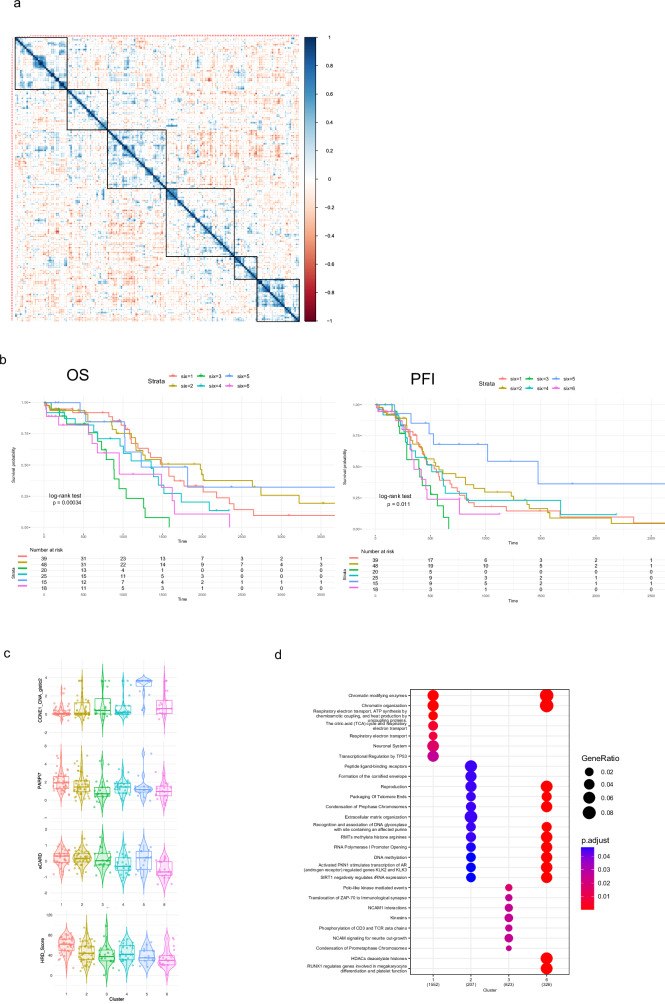
Clustering of patients revealed poor prognosis. **a** Pearson correlation matrix of ovarian cancer patients based on the expression of the 29 lncRNAs. Matrix was clustered using k-means into six clusters. **b** Overall survival (OS) and progression free survival (PFI) analysis using the Kaplan-Meier curve of the six clusters from (**a**). *p* values were calculated using log-rank test. **c** The six clusters were analyzed for *CCNE1* CNA, PARPi7-level, cCARD-level and HRD-score using violin blot. **d** Analysis of enriched pathways in cluster 1, 2, 3, and 6 using the Reactome pathway analysis.

Based on the expression profiles of the 29 lncRNAs, we identified six distinct clusters of patients within the ovarian TCGA dataset (Fig. 2a and Supplementary Table 3). These clusters exhibited significant differences in gene expression patterns (Supplementary Fig. 3b), which led to substantial separations in both overall survival (OS) and progression-free survival (PFS) (Fig. 2b). Notably, after controlling for the confounding factors of age and tumor purity, the statistical significance of these associations remained robust (Supplementary Fig. 3c and d).

Further analysis using a multivariate Cox proportional hazards model revealed that the clusters 3 (HR = 4.92, 95% CI: 2.26–10.70, *p* < 0.0001) and 6 (HR = 4.10, 95% CI: 1.77–9.53, *p* = 0.001) were most significantly associated with poor survival outcomes, further highlighting the prognostic value of these lncRNA-based clusters in ovarian cancer (Supplementary Fig. 3d).

When analyzing the different characteristics of each cluster we found that cluster 1 best describes patients with high HRD- and PARPi7-score whereas cluster 3 represents patients with a low HRD- and PARPi7-scores (Fig. 2c). Cluster 6 shows additionally to a low HRD- and PARPi7-score a low eCARD-score. Cluster 5 in contrast, represents patients with tumors expressing high levels of *CCNE1* or harboring an amplification of *CCNE1*. We next investigated which are the most determining lncRNAs for each cluster. We therefore used a random forest model (Supplementary Fig. 3e, f). ENSG00000257671.1 was the most determining lncRNA that is expressed highest in cluster 6 followed by cluster 4 (Supplementary Fig. 3g). ENSG00000261183.1 and ENSG00000272172.1 showed the highest expression in HRD cluster 1. In the *CCNE1* cluster 5 the lncRNAs ENSG00000272635.1 and ENSG00000267575.2 were highest expressed and in the cluster 3 that represents HR proficient tumors ENSG00000229874.2 had the highest expression. Based on the differential gene expression in the patient clusters we analyzed which pathways were enriched (Fig. 2d). Notably, cluster 1 and 6 showed changes in chromatin organization whereas cluster 2 and 6 are enriched for changes in DNA methylation and RNA Polymerase I promoter opening. Cluster 6 also showed a unique enrichment for changes in histone deacetylase (*HDAC*), whereas cluster 3 was enriched for changes in chromosome condensation and kinesins. Those data indicate the panel of lncRNAs could be useful to predict patient outcome.

### LncRNA expression and drug response in cell lines

**Fig. 3:**
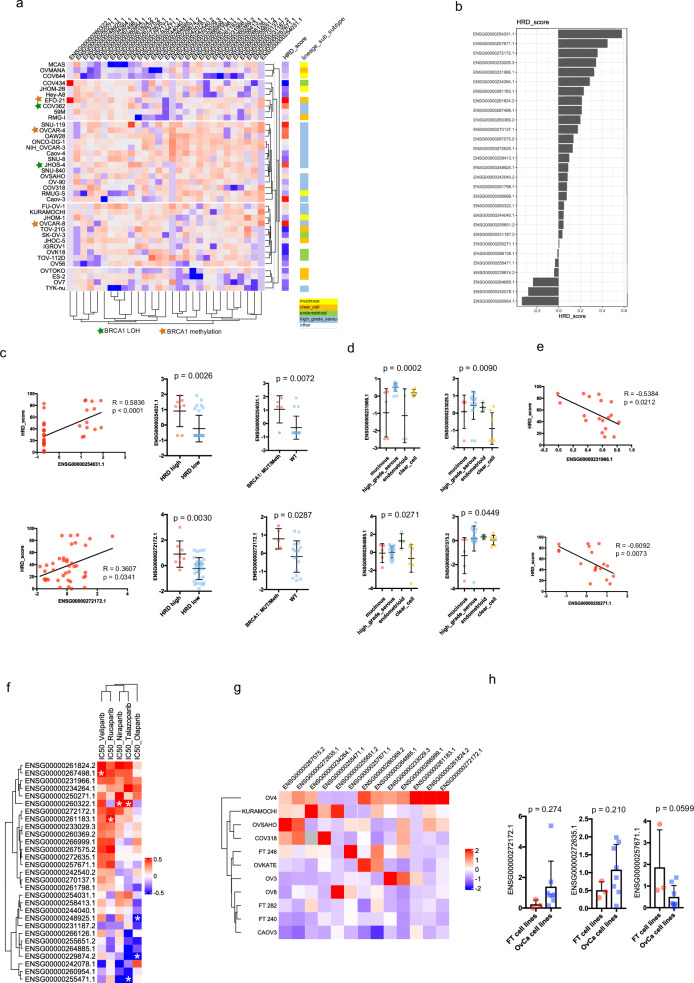
Cell line expression and drug response. **a** Heatmap depicting the normalized expression levels of 29 selected lncRNAs and HRD- scores across ovarian cancer cell lines from the CCLE. Expression values were normalized across cell lines to allow comparison. **b** Pearson correlation coefficients between individual lncRNA expression levels and HRD scores across CCLE ovarian cancer cell lines are shown. **c** Top row: Scatter plot of HRD score versus ENSG00000254031.1 expression (left), and comparisons of ENSG00000254031.1 expression between HRD-high (HRD ≥ 52) vs HRD-low (middle) and *BRCA1*-mutated/methylated vs *BRCA1*-unaltered cell lines (right). Bottom row: Similar analyses for ENSG00000272172.1. Statistical comparisons were performed using Pearson correlation and two-tailed unpaired *t*-tests. **d** Expression profiles of four representative lncRNAs across ovarian cancer cell lines stratified by histological subtype (mucinous, high-grade serous, endometrioid, clear cell). Differences were assessed using multi-way ANOVA to account for subtype-specific variance. **e** Pearson correlation plots showing HRD scores versus expression of ENSG00000231966.1 (top) and ENSG00000250271.1 (bottom) specifically in HGSC cell lines. **f** Correlation matrix showing Pearson correlation coefficients between the IC50 values of five PARP inhibitors and expression of the 29 lncRNAs. Statistically significant correlations (*p* < 0.05) are marked with an asterisk. **g** Heatmap illustrating relative expression of selected lncRNAs across seven ovarian cancer cell lines and three immortalized fallopian tube epithelial cell lines, as measured by real-time qPCR. Expression was normalized to *GAPDH* and represented as fold change. **h** Bar plots comparing expression levels of selected lncRNAs between immortalized fallopian tube epithelial cell lines and ovarian cancer cell lines, as measured by real-time qPCR. Data shown represent one biological replicate of three independent experiments. Statistical significance was assessed using unpaired two-tailed t-tests; differences with *p* values < 0.05 were considered significant.

Next, we analyzed the expression of the 29 lncRNAs in different ovarian cancer cell lines. We therefore used the RNAseq dataset of the cancer cell line encyclopedia (CCLE) that was recently analyzed for HR-deficiency (Supplementary Table 4)^38^. Clustering of the different cell lines based on the expression of the 29 lncRNAs revealed no major subgroups of cell lines that were enriched for either *BRCA1* inactivation or differential HRD-scores (Fig. 3a). However, we found that the expression of several lncRNA correlated significantly with the HRD scores of the cell lines (Fig. 3b). Notably, ENSG0000025031.1 (*R* = 0.58, *p* < 0.0001) and ENSG00000272172.1 (*R* = 0.36, *p* = 0.031) correlated positively with the HRD-score and were significantly higher expressed in cell lines that had a high HRD-score (HRD ≥ 52) or a *BRCA1* inactivation when compared to *BRCA* wild-type and HRD negative cell lines (Fig. 3c). Since the ovarian cancer cell lines were derived from patients with different histological subtypes, we analyzed the lncRNA expression of those subtypes. Using anova multi-way test, we identified four lncRNAs that show significant differences (Fig. 3d). Furthermore, when we analyzed only cells derived from HGSCs, we observed that both ENSG00000231966.1 (*R* = −0.54, *p* = 0.0212) and ENSG00000250271.1 (*R* = −0.61, *p* = 0.0073) showed a strong negative correlation to the HRD-score (Fig. 3e).

We then used the available IC50 values from five PARP inhibitors: olaparib, rucaparib, talazoparib, veliparib, and niraparib, and calculated the pearson correlation coefficient for each lncRNA (Fig. 3f). While rucaparib, talazoparib, veliparib, and niraparib clustered together, olaparib showed an individual correlation pattern with few lncRNAs regulated in common with the other PARPis. Since the dataset was limited to 21 cell lines, only few correlations reached significance. We observed that the expression of ENSG00000255471.1, ENSG00000248925.1, and ENSG00000229874.2 correlated negatively with PARPi sensitivity while the expression of ENSG00000260322.1, ENSG00000261183.1, and ENSG00000267498.1 correlated positively with sensitivity to different PARPi (white stars in heat map, Fig. 3f).

In agreement with Takamatsu et al., who found that HRD-score and PARP inhibitor response are unrelated in the cancer cell line encyclopedia dataset, we did not identify lncRNAs that were correlated to both HRD-score and PARP inhibitor response^38^.

We then developed a real time PCR assay to quantify the lncRNAs in our cell lines under different treatment conditions. We used primer pairs for lncRNA that produced consistent ct values, melting curves and were detectable using real time PCR in different cell lines (Fig. 3g). We next compared the lncRNA expression of 7 ovarian cancer cell lines to 3 immortalized fallopian tube cell lines (Fig. 3h). The expression of three lncRNAs, including ENSG00000272172.1, showed a difference between FT cell lines and ovarian cancer cell lines, but did not reach significance (p = 0.27) (Fig. 3h). Overall, our data indicate that lncRNAs such as ENSG00000272172.1 and ENSG0000025031.1 are connected to HR deficient phenotypes.

### ENSG00000272172.1 expression is higher in HRD-positive patients

**Fig. 4:**
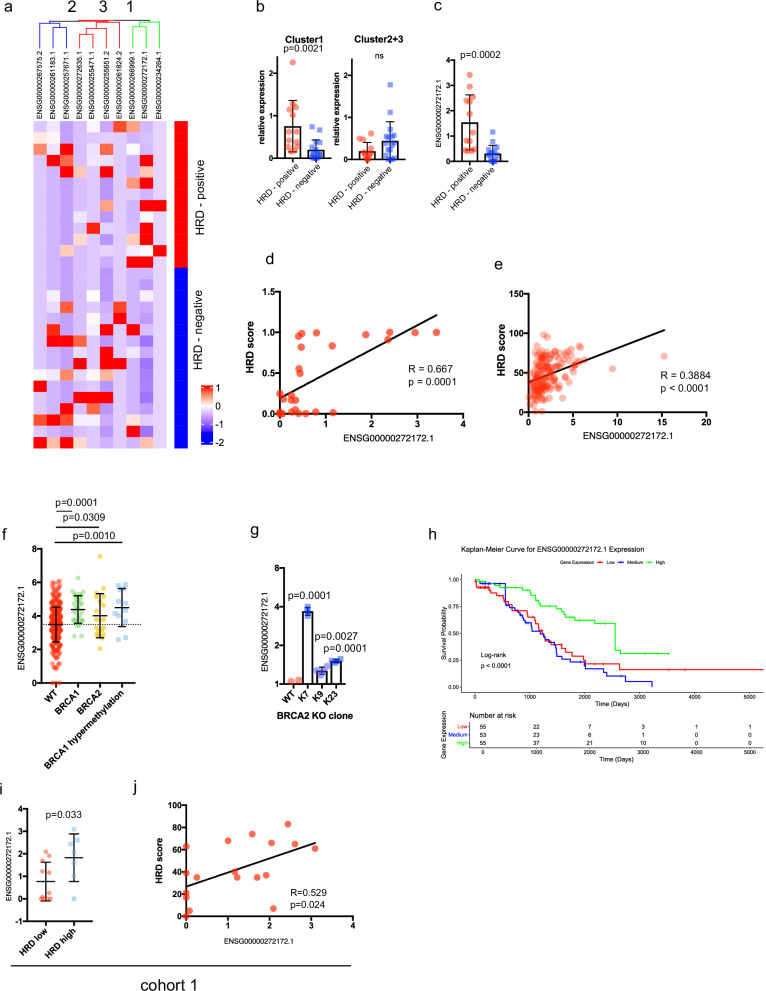
ENSG00000272172.1 is higher expressed in patients with HRD. **a** Heatmap showing normalized expression levels of selected lncRNAs, as measured by real-time qPCR, in formalin-fixed paraffin-embedded (FFPE) tumor samples stratified by HR status (HR-deficient vs HR-proficient), as determined by genetic profiling. Expression was normalized to housekeeping gene (GAPDH) and was z-score normalized across samples. **b** Bar plot comparing expression of lncRNA expression clusters (Cluster 1 vs Clusters 2 and 3) between HR-deficient and HR-proficient tumors. Statistical significance was determined using unpaired *t*-tests. **c** Bar plot comparing expression levels of ENSG00000272172.1 in HR-deficient versus HR-proficient tumors from the same FFPE cohort. Statistical significance was determined using unpaired *t*-tests. **d** Scatter plot showing Pearson correlation between HRD scores and ENSG00000250271.1 expression across ovarian cancer samples analyzed in (**a**). **e** Pearson correlation of HRD score and ENSG00000250271.1 expression in ovarian cancer samples from The Cancer Genome Atlas. **f** Expression levels of ENSG00000272172.1 in patient tumors stratified by *BRCA1/2* mutation status: wild-type (WT), mutated, or *BRCA1* promoter hypermethylation. Statistical comparisons used ANOVA multi comparison test. **g** Expression of ENSG00000272172.1 in OVCAR8 cells following *BRCA2* knockdown using CRISPR/Cas9. RNA was extracted and expression was measured by qPCR (ΔCt method). **h** Kaplan–Meier analysis of overall survival in patients stratified into high (red), medium (blue), and low (green) ENSG00000272172.1 expression tertiles. Survival differences were assessed using the log-rank test. **i** Comparison of ENSG00000272172.1 expression in plasma samples from patients with high vs low HRD scores, based on a predefined threshold in cohort 1. **j** Scatter plot showing Pearson correlation between HRD scores and ENSG00000250271.1 expression in plasma samples of cohort 1.

We isolated RNA from FFPE sections that were derived from patients who were participating in the observational AGO-TR1 trial (NCT02222883, Supplementary Table 5). The HR proficiency of the samples was previously determined by using a copy-number-based ovarian cancer–specific classifier as well as gene panel sequencing and methylation status analysis^39^. We compared the lncRNAs expression of 13 patients harboring HR deficient ovarian cancers to 16 HR proficient ovarian cancers. Real-time PCR analysis of 10 lncRNAs lead to the separation of three main clusters (Fig. 4a). The HR deficient patients expressed significantly more of the lncRNAs in cluster 1 compared to the HR proficient patients (Fig. 4b). HR proficient patients displayed more frequently high expression of lncRNA in cluster 2 and 3. When analyzing the individual lncRNAs we identified that ENSG00000272172.1 was significantly higher expressed in the HR deficient patients (Fig. 4c). In those patients the copy number derived HRD-scores correlated significantly with the expression of ENSG00000272172.1 (Pearson = 0.667, *p* = 0.0001, Fig. 4d). This was in agreement with the TCGA-OV dataset, which also demonstrated a positive correlation of ENSG00000272172.1 with the HRD-score (Figs. 1e and 4e, Pearson = 0.3884, *p* = 2.3 × 10^-7^) that was derived by measuring genome-wide loss-of-heterozygosity (LOH), telomeric allelic imbalance (TAI), and large-scale state transitions (LST)^40–42^.

Additionally, ENSG00000272172.1 expression was also positively correlated with PARPi7-score (Pearson = 0.19, *p* = 0.012) and eCARD-score (Pearson = 0.23, *p* = 0.018) in the TCGA dataset (Supplementary Fig. 4a). While ENSG00000272172.1 was most highly expressed in ovarian cancers compared to other cancer types in the TCGA pan-cancer dataset, it also correlated significantly with PARPi7 and HRD-score in multiple other cancer types, such as Lung Squamous Cell Carcinoma (LUSC), Cervical Squamous Cell Carcinoma and Endocervical Adenocarcinoma (CESC), Kidney Chromophobe (KICH), Head and Neck Squamous Cell Carcinoma (HNSC), Stomach Adenocarcinoma (STAD) and Prostate Adenocarcinoma (PRAD) (Supplementary Fig. 4b–d).

When analyzing the TCGA ovarian cancer dataset we observed that ENSG00000272172.1 was overexpressed in *BRCA1* and *BRCA2* mutated tumors as well as tumors with *BRCA1* hypermethylation, indicating that an overexpression of ENSG00000272172.1 can be the result of different HR-deficiency mechanisms (Fig. 4f)^43^. We did not observe differences between somatic and germline *BRCA1* or *BRCA2* mutations. To confirm the link between the loss of *BRCA2* and ENSG00000272172.1 we analyzed *BRCA2* CRISPR knockout cell lines, where we observed a significant increase in ENSG00000272172.1 expression in all clonal cell lines (Fig. 4g). Patients with a higher expression of ENSG00000272172.1 also had a significantly better overall survival (*p* < 0.0001) (Fig. 4h). When we adjusted the OS analysis for age and tumor purity, ENSG00000272172.1 expression remained significantly associated with OS. Specifically, the “High” expression group of ENSG00000272172.1 exhibited a significant positive association with both OS (hazard ratio = 0.3990, 95% CI: 0.2271–0.7009, *p* = 0.00139), indicating that high expression of this lncRNA is linked to better survival outcomes. In contrast, the “Medium” expression group did not demonstrate a statistically significant effect compared to the “low” expressing group (hazard ratio = 1.2803, 95% CI: 0.7712–2.1254, *p* = 0.33939).

### ENSG00000272172.1 can be detected in the plasma of ovarian cancer patients

To detect the ENSG00000272172.1 in low quantities patient plasma samples, we used the LNA technology probes (QuantiNova, Qiagen). Using 200 µl plasma for RNA isolation we were able to detect ENSG00000272172.1 in 20 out of 24 plasma samples by real time PCR (Plasma HIPO H059 cohort 1, Supplementary Table 5, *n* = 24). Surprisingly, we did not detect higher levels of ENSG00000272172.1 in the plasma of *BRCA1* mutant (*n* = 7) carriers compared to *BRCA1* wild type (wt) (*n* = 17) patients (Supplementary Fig. 4e). When analyzing the *BRCA1/2 *wt patients, we found that patients with a high HRD-score (*n* = 7) have significantly higher plasma levels of ENSG00000272172.1 when compared to patients with low (*n* = 11) HRD-scores (Fig. 4i). The HRD-score, defined by quantification of LOH, TAI and LST, also showed a significant correlation to ENSG00000272172.1 expression with a pearson correlation of 0.529 (*p* = 0.024)(Fig. 4j). To confirm these results we analyzed an independent set of plasma samples (Plasma Mannheim Cohort 2, Supplementary Table 5, *n* = 16). Here, we also did not observe elevated ENSG00000272172.1 levels in patients with *BRCA1/2* mutations (Supplementary Fig. 4f). However, when comparing the patients with a high HRD-score to patients with a low HRD-score, which was determined using the Myriad myChoice HRD test, we detected a significant ENSG00000272172.1 increase in HRD-positive patients (Supplementary Fig. 4g).

In summary, our data suggests that ENSG00000272172.1 might be a useful biomarker to determine the HRD status in plasma or tissue of *BRCA1/2 *wt patients.

### Loss of ENSG00000272172.1 increases DNA damage

**Fig. 5:**
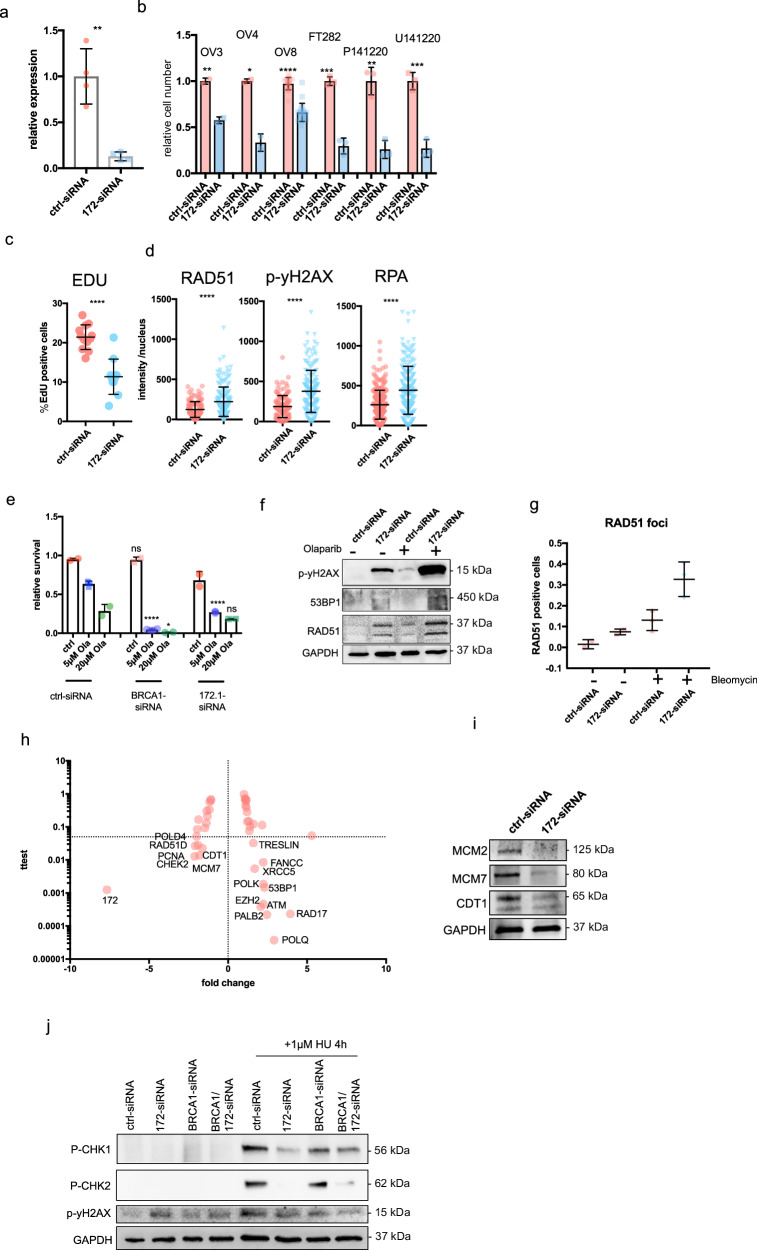
Loss of the lncRNA ENSG00000272172.1 increases DNA damage. **a** Expression of ENSG00000272172.1 in OVCAR8 cells 72 h post-transfection with either control siRNA or siRNA targeting ENSG00000272172.1, measured by real-time PCR using the ΔCt method and normalized to *GAPDH*. Displayed is one of three representative biological replicates. **b** Relative cell number in a panel of ovarian cancer cell lines (*n* = 6) 72 hours after transfection with control or ENSG00000272172.1-targeting siRNA, quantified using the MTS cell viability assay. Data normalized to control-siRNA-treated cells. **c** Quantification of EdU-positive (actively replicating) OVCAR8 cells following 72 h after siRNA-mediated knockdown of ENSG00000272172.1, assessed by immunofluorescence microscopy. Value represents percentage of positive nuclear EDU cells per field of view. **d** Nuclear intensities of RAD51, phosphorylated γH2AX (p-γH2AX), and RPA were quantified in OVCAR8 cells 72 h post-knockdown, using immunofluorescence and automated image analysis. **e** Relative survival of OVCAR8 cells treated with control-siRNA, *BRCA1*-siRNA, or ENSG00000272172.1-siRNA. 24 h after siRNA treatment, the cells were treated for 72 h with 5 µM or 20 µM of olaparib. Cell viability was measured by a MTS assay. **f** Western blot analysis of DNA damage and repair markers (p-γH2AX, 53BP1, RAD51) in OVCAR8 cells treated with control or ENSG00000272172.1-siRNA. Cells were exposed to 20 µM olaparib 24 h post-transfection and lysed after an additional 48 h. GAPDH was used as a loading control. **g** Quantification of RAD51-positive nuclei in OVCAR8 cells treated with control or ENSG00000272172.1-siRNA. Cells were treated with bleomycin (10 µg/mL, 1 h) 48 h post-transfection, followed by a 16-h recovery period before fixation. **h** Volcano plot showing differential gene expression in OVCAR8 cells following ENSG00000272172.1 knockdown versus control-siRNA treatment, based on real-time PCR profiling of a selected gene panel. Fold changes and *p* values were calculated using the ΔCt method. **i** Western blot analysis of MCM2, MCM7, CDT1 and GAPDH in OVCAR8 cells treated with control or ENSG00000272172.1-siRNA. **j** Western blot showing phosphorylation levels of checkpoint proteins CHK1, CHK2, and γH2AX in cells transfected with control-siRNA, *BRCA1*-siRNA, or ENSG00000272172.1-siRNA. At 48 h post-transfection, cells were treated with 2 mM hydroxyurea (HU) for 1 h before lysis. For all quantified assays, results are representative of at least three biological replicates unless otherwise noted. Statistical analysis was performed using unpaired two-tailed Student’s *t*-tests. Significance is indicated as *p* < 0.05 (*), *p* < 0.01 (**), *p* < 0.001 (***), and *p* < 0.0001 (****).

To elucidate the biological function of ENSG00000272172.1, we used a pool of three different siRNA to downregulate its expression (Fig. 5a, Supplementary Fig. 5a). The knock down led to a significant reduction in proliferation in multiple cell lines, including two primary cell lines (Fig. 5b). The reduction in proliferation could also be observed in reduced EDU incorporation (Fig. 5c).

The knockdown led to an increase in DNA damage response, measured through an increase in nuclear phospho-yH2AX, RAD51, and RPA staining (Fig. 5d).

We then asked if the loss of ENSG00000272172.1 might sensitize cancer cells to PARPi, similarly to the loss of *BRCA1* or *BRCA2*. We therefore knocked down either *BRCA1* or ENSG00000272172.1 and subsequently treated the cells with different doses of olaparib. The knock down of ENSG00000272172.1 exhibited an additive effect with PARPi treatment, but we did not observe synergistic effects comparable to the loss of *BRCA1* (Fig. 5e). Interestingly, the increase in DNA damage was amplified in ENSG00000272172.1 knock-down cells treated with olaparib or the DSB inducer bleomycin (Fig. 5f, g).

In the next step, we analyzed changes in gene expression of a DNA damage gene panel, following the knock down of ENSG00000272172.1 (Fig. 5h). Interestingly, besides an upregulation of genes involved in multiple DNA-damaging response pathways, such as *RAD17*, *53BP1*, and *FANCC*, we also observed changes in origin of replication genes, such as *MCM7* and *CDT1*. The reduced expression of *MCM2, MCM7*, and *CDT1* was confirmed by western blot analysis (Fig. 5i). Surprisingly, we also observe almost complete abrogation of CHK1 or CHK2 activation following ENSG00000272172.1 siRNA (Fig. 5j), indicating that the p-yH2AX activation was CHK1/2 independent. Overall, the knockdown of ENSG00000272172.1 led to a significantly reduced proliferation and an increased DNA damage response, accompanied by changes in expression of genes involved in DNA damage response and origin of replication genes.

### Loss of ENSG00000272172.1 increases DNA replication speed

An increase in replication speed can lead to replication stress and DNA damage^44^. PARP inhibitors accelerate the replication speed, leading to replication fork instability, which contributes to the therapeutic effects in HR deficient cancer cells.

**Fig. 6:**
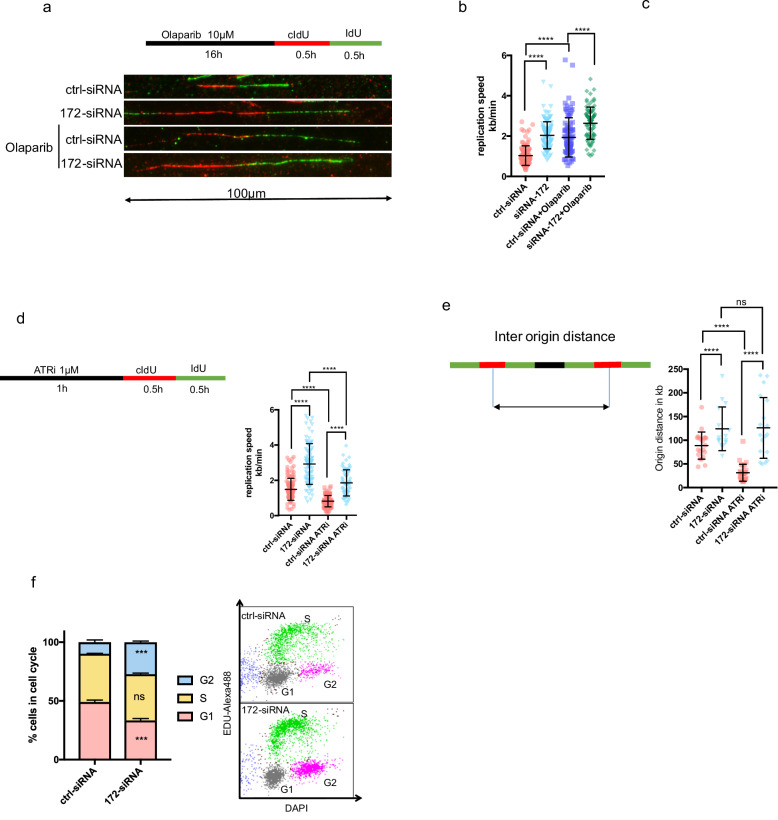
Loss of ENSG00000272172.1 increases DNA replication speed and alters replication dynamics. **a** Upper panel: Schematic of the DNA fiber assay. OVCAR8 cells were transfected with either control siRNA or siRNA targeting ENSG00000272172.1. After 48 h, cells were treated with 20 µM olaparib for 16 h, followed by sequential labeling with CldU and IdU (30 min each). DNA fibers were then spread and stained with specific antibodies, as described in the Methods. Lower panel: Representative immunofluorescence images of labeled DNA fibers. **b** Quantification of DNA replication speed in OVCAR8 cells treated as in (**a**). Replication track length was measured in microns and converted to kilobases per minute using a standard conversion factor. **c** Fork symmetry analysis of individual replication forks. The ratio of CldU to IdU tract length was calculated to assess fork progression balance. **d** Left: Schematic of the modified DNA fiber assay. OVCAR8 cells were transfected with siRNAs, and 48 h later, treated with 1 µM ATR inhibitor AZD6738 for 1 hour prior to sequential labeling with CldU and IdU. Right: Quantification of replication speed under ATR-inhibited conditions. **e** Left: Schematic of origin-to-origin distance measurement. Experimental conditions were identical to (**d**). Right: Quantification of inter-origin distances, measured as the distance between adjacent replication initiation sites on individual DNA fibers. **f** Left: Cell cycle distribution of OVCAR8 cells transfected with control or ENSG00000272172.1-targeting siRNA, assessed by flow cytometry following DNA content staining (EDU and DAPI). All fiber measurements represent data from at least three independent experiments with ≥100 fibers analyzed per condition. Data are shown as mean ± SD. Statistical comparisons were performed using unpaired two-tailed Student’s *t*-tests. Significance is indicated as *p* < 0.05 (*), *p* < 0.01 (**), *p* < 0.001 (***), and *p* < 0.0001 (****).

Since we observed changes in *RECQL4*, *TRESLIN*, as well as MCM proteins, which are known to have effects on the replication origin machinery, we analyzed the replication speed using fiber spread assay (Fig. 6a). Similarly to olaparib, the knockout of ENSG00000272172.1 led to an increase in replication speed (Fig. 6a, b). The knockout of ENSG00000272172.1 led to a further increase in replication speed of cancer cells treated with olaparib, indicating different mechanisms (Fig. 6b). We did not observe significant changes in fork symmetry, which would indicate changes in fork stability (Fig. 6c).

Two mechanisms can lead to an increase in replication speed: (1) An increase in the expression of proteins, such as TRESLIN, increases the replication speed directly. (2) The increase in replication speed counterbalances the reduction in the number of replication origins due to the increased interorigin distance. The cell thereby increases the probability of successfully replicating its genome during a single cell cycle.

Previous research has established that ATR inhibitors (ATRi) trigger the activation of all dormant replication origins, thereby decreasing the inter-origin distance. ATRi (AZD6738, 1 µM) treatment resulted in a lower replication speed (Fig. 6d). Despite the reduction in replication speed observed in both the control and knockdown cells following ATRi treatment, a significant difference between the two groups remained, suggesting that the loss of ENSG00000272172.1 has a greater impact on origin distance than on replication speed. This hypothesis is further supported by direct measurements of origin distance, which revealed a significant decrease in control cells, but no change in knockdown cells upon ATRi treatment. This suggests that there is no reserve of dormant origins in knockdown cells (Fig. 6e).

Since licensing of replication origins is restricted to the G1-phase of the cell cycle to prevent re-replication and over-amplification, a shorter G1 phase provides less time for loading replication origins onto the DNA. Our results showed that siRNA knockdown of ENSG00000272172.1 resulted in a marked decrease in cells in the G1 phase and an increase in cells in the G2 phase of the cell cycle (Fig. 6f). These findings suggest that the expression of ENSG00000272172.1 leads to a longer G1 phase, providing more time for replication origin loading and protection against replication stress.

## Discussion

Targeted therapy has made significant advancements in recent years. A prime example is that patients with an inactive HR pathway are particularly sensitive to PARPi^4,5^. PARP1 molecule is responsible for repairing single-strand breaks (SSBs), and PARP inhibitors are able to trap the PARP1 molecule at the SSB site, preventing it from recruiting the necessary proteins for repair by a process known as parylation^45,46^. As a result, when the SSB-PARP1 complex encounters a replication fork, it is converted into a double-strand break DSB that can only accurately be repaired through HR. Based on this mechanism of action, PARPis have been approved for multiple cancer types, and multiple clinical studies are ongoing^47^. Clinical studies have revealed that, besides patients with somatic or germline *BRCA1/2* mutations, a much broader patient population benefits from PARPi treatment^48^. Today, the approval of PARPi as first-line therapy is not limited to *BRCA1/2* mutation carriers. However, the magnitude of benefit differs significantly based on *BRCA1/2* mutation status. Different approaches have been developed to identify patient populations that benefit from PARPi treatment.

The most frequently used approach to predicting PARPi response is to perform targeted hybrid capture and next-generation sequencing (NGS) technology to identify mutations in *BRCA1/2* genes^49^. While this approach can detect mutations in *BRCA1* or *BRCA2*, it is not able to identify other mechanisms that lead to the functional inactivation of the HR pathway, such as *BRCA1* methylation or mutations in alternative genes.

An alternative approach is to quantify genomic alterations that are characteristic for HR-deficient cells to calculate a HRD-score^50,51^. It is important to note that these tests only inform about past events, and are not able to determine if cancer cells have acquired resistance mechanisms such as reversion mutations.

The identification of biomarkers that predict PARP inhibitor response is clinically important. To address this, we aimed to identify a panel of lncRNAs that could predict PARP inhibitor response. We chose lncRNAs because previous studies have demonstrated that they are highly tissue-specific, relatively stable when released into the blood, and may have roles in DNA stability^17,18^.

For our initial analysis, we used The Cancer Genome Atlas datasets, as these are well-characterized and include different scores to predict HRD and drug sensitivity. We selected a panel of 29 lncRNAs from over 10,000 lncRNAs in the TCGA dataset. To identify a robust panel, we included genome-based and expression-based scores^9,34–36^.

We applied clustering methods and identified distinct molecular clusters with significantly different survival outcomes. These clusters also varied in *CCNE1* amplification status as well as in their predicted PARPi sensitivity, eCARD scores, and HRD scores. Notably, Cluster 5 showed the best progression-free interval prognosis, which is counterintuitive given its enrichment for CCNE1 amplification, a feature typically associated with poor survival and resistance to PARPi. Although *CCNE1* amplification is generally linked to adverse outcomes, we did not observe the expected negative impact on survival in this cluster. One possible explanation is that our HRD and PARPi sensitivity assessments relied on gene expression-based and genomic signatures derived from other datasets, which may not fully capture true clinical responses. Furthermore, while Cluster 5 showed improved PFI, this was not reflected in overall survival, and these patients also exhibited lower HRD and PARPi scores, consistent with a more resistant tumor profile. The relatively small sample size of Cluster 5 may also limit statistical power and contribute to these findings. Overall, these results underscore the need for validation in larger, independent cohorts and suggest that factors beyond *CCNE1* status may modulate treatment response.

Our study has several limitations, including the inability to validate the functional relevance of the 29 identified lncRNAs in an independent dataset. Additionally, we relied on precomputed genomic and transcriptomic scores to infer HRD status and PARPi sensitivity, both of which have known limitations. For instance, current HRD tests do not consistently predict treatment response, and many patients relapse despite being HRD-positive and receiving PARPi therapy. Future studies are needed to investigate the biological roles of these lncRNAs and evaluate their prognostic and predictive potential in independent patient cohorts.

When analyzing the expression of the 29 lncRNAs in the cell line expression from the Cancer Cell Line Encyclopedia dataset, we neither observe a clustering of cell lines based on HRD-score nor on the BRCA1 inactivation status^52^. However, when analyzing individual lncRNAs, we found that ENSG00000272172.1 and ENSG00000254031.1 were strongly associated with HRD-score and *BRCA1* status. However, lncRNAs that were associated with HRD and *BRCA1* mutation status did not overlap with the lncRNAs that were associated with sensitivity towards different PARP inhibitors. This is in line with the finding from Takamatsu and colleagues, who found no association between HRD-score and PARP inhibitor sensitivity in cell lines using the same dataset^38^. It is unclear why the HRD-score is a poor predictor for PARP inhibitor sensitivity in cell lines. Potentially, primary patient derived cultures represent a better cell culture model.

The differences observed between cell line based analyses (CCLE) and tumor-derived (TCGA) results likely reflect biological and technical distinctions, including reduced heterogeneity, clonal selection, and altered microenvironmental context in cell lines, as well as disparities in RNA processing methods.

Comparing the expression of three immortalized fallopian tube cell lines to a panel of ovarian cancer cell lines, we observed differential expression in three lncRNAs, but this did not reach significance. Notably, these lncRNAs included ENSG00000272172.1, which significantly correlated positively with the HRD-score in four independent datasets, including the TCGA public dataset, cell line CCLE dataset, and RNA that was isolated from paraffin tumor sections as well as from patient plasma. It is also important that the HRD scores in the datasets were generated using different methodologies, strengthening the notion of ENSG00000272172.1 as a robust biomarker for HRD. ENSG00000272172.1 was also higher expressed in germline and somatic *BRCA1* and *BRCA2* mutated tumors, as well as *BRCA1*-hypermethylated tumors, indicating that this lncRNA is upregulated independently on the mechanism that causes HRD. This is further supported by the finding that ENSG00000272172.1 levels are also elevated in cases with a high HRD-score that harbor no *BRCA1* or *BRCA2* alteration.

The fact that we were able to detect lncRNA ENSG00000272172.1 in RNA formalin-fixed paraffin-embedded (FFPE) samples as well as plasma samples indicates a high stability of this lncRNA. This, together with its strong overexpression in HRD tumors, make it a promising biomarker to predict PARP inhibitor response.

We acknowledge that the quantification of circulating lncRNAs remains a methodological challenge, primarily due to the absence of consensus on appropriate normalization strategies^31^. In this study, we normalized based on the volume of plasma used for RNA extraction, an approach widely used in the field but known to be imperfect. This method does not account for inter-sample variability in RNA content or extraction efficiency, and it lacks the correction power offered by stable endogenous controls. However, RNA yields from plasma or serum are often too low to be reliably quantified by NanoDrop, and levels can be artificially elevated in disease states such as cancer. These factors complicate normalization based on total RNA quantity, and in such cases, volume-based input may be more practical and consistent. Nevertheless, the field still lacks standardized endogenous reference genes or spike-in strategies, and this remains a major obstacle to the reproducibility and clinical translatability of circulating RNA biomarker studies.

It has to be determined how useful ENSG00000272172.1 could be used as an independent predictor or whether it is best used in combination with other markers.

While we detected a strong correlation of ENSG00000272172.1 in plasma samples to the individual HRD-scores, we did not observe an increase in patients with a *BRCA1* mutation. The release of RNAs is influenced by multiple factors such as tumor size, secretion, exosome formation, and angiogenesis. It has to be determined what factors could influence the release of this lncRNA into the plasma and if it is released in microvesicles, which could explain its stability.

We also had no information of how many of those *BRCA1* mutation carries retained a wild type *BRCA1* allele. It was recently shown that a substantial percentage of *BRCA1* and *BRCA2* mutated cancers do not lose their wild type allele through locus-specific LOH^43^.

To our knowledge ENSG00000272172.1 (alternative name: RP13-582O9.7) has been described only in one publications: Ma et al. have shown its upregulation in hepatocellular cancer compared to normal tissue and its association with the Jun activation domain-binding protein 1(JAB1) expression^53^. Genomically, ENSG00000272172.1 is located at chromosome 8q24 and overlaps with the promoter region of ZNF696, although we observed only weak co-expression between these transcripts, suggesting independent regulation. Both genes are also transcribed in opposite directions.

Our analysis also revealed a moderate positive correlation between 8q24 copy number alterations (CNA) and HRD status in the TCGA ovarian cancer cohort (*R* = 0.32), suggesting that amplification of this region may be linked to homologous recombination deficiency–associated mechanisms. While 8q24 CNA did not directly correlate with overall survival in our dataset, its association with HRD underscores the potential functional relevance of this locus and supports further investigation into how specific transcripts within this amplified region, including ENSG00000272172.1, may contribute to treatment response and genomic instability.

Since ENSG00000272172.1 correlates with the HRD-score, PARPi7-score, and eCARD-score, we suspected that it might be involved or co-regulated with important DNA repair pathways. We found that the knockdown led to an upregulation of many DNA repair genes and downregulation of origin of replication genes. The increased replication speed, which was most likely responsible for the increase in DNA damage, was likely caused by the reduced number of replication origins. Recently, it was shown that olaparib accelerated the replication apparatus and led to increased replication fork collapse, which contributes to the synthetic lethal effects of olaparib in HR deficient patients^44^. To test whether the increased replication speed was caused directly by proteins that regulate the replication complex or indirectly as a compensation for increased distance between replication origins, we pre-treated the cells with an ATRi prior to measuring replication speed. ATR inhibitors have been shown to induce unscheduled firing of dormant origins^54^. While the ATR inhibitors reduced the replication speed in the ENSG00000272172.1 knockdown cells, it was still significantly faster than the cells treated with control siRNA. Additionally, the distance between replication origins was not reduced when treated with ATR inhibitors. These data indicate that the increase in replication speed is a result of compensating for the reduced number of replication origins. A reduced number of activated and dormant origins has been shown to render cells sensitive to replication stress and lead to DNA damage^55,56^.

Our findings also suggest that HR-deficient cells might upregulate ENSG00000272172.1 as a means to stabilize the genome during replication stress.

Future studies will need to elucidate the precise mechanisms by which ENSG00000272172.1 contributes to genome stability, including direct interactions with key replication proteins, cis- or trans-regulation of neighboring or distal genes (such as ZNF696 or DNA repair factors), and roles as a molecular scaffold or decoy, for example, acting as a microRNA sponge to fine-tune DNA damage response pathways. Overall, the findings can open potential avenues of research towards understanding how lncRNAs can impact DNA repair and replication processes, which could eventually lead to therapeutic targets for treating cancer.

Recent studies have described mecanisms of other lncRNAs in regulating the DNA damage response and maintaining genomic integrity in cancer. Notably, NORAD was shown to stabilize the PUMILIO proteins, preventing degradation of mitotic and repair genes, with its loss causing chromosomal instability^57^. The lncRNA ANRIL has been shown to promote HR-mediated DNA repair by maintaining ATR protein stability in lung cancer models^58^. Similarly, the lncRNA scaRNA2 binds ATR and thereby promotes efficient DNA end resection during homologous recombination repair^59^. PCAT-1 represses BRCA2, inducing HR deficiency and synthetic lethality with PARP inhibitors in prostate cancer^60^. Additionally, DDSR1 supports BRCA1 recruitment during HR^61^, while targeting NEAT1 sensitices ovarian cancer cells to PARPi by regulating RAD51^62^. These findings underscore that lncRNAs are key modulators of DDR and represent potential targets for enhancing cancer therapy efficacy.

The identified lncRNA biomarker ENSG00000272172.1 demonstrates significant promise for future clinical applications, as it was detectable not only in tumor tissue but also in plasma-derived RNA, underscoring its potential for non-invasive biomarker development. This opens potential avenues for liquid biopsy approaches, where monitoring lncRNA expression could complement or even replace more invasive tissue sampling methods. Moreover, our approach highlights the feasibility of computationally prioritizing and experimentally validating lncRNAs for their association with HRD status, offering a robust framework for discovering potential biomarkers. As PARP inhibitors gain broader clinical use, our findings support the potential development of a qPCR-based diagnostic assay that could stratify patients based on ENSG00000272172.1 expression. Such an assay would be particularly beneficial in clinical settings where genomic testing for BRCA mutations is either limited or inconclusive. Additionally, this approach could be extended to other cancers characterized by HRD phenotypes, such as triple-negative breast cancer and prostate cancer, broadening the impact of personalized cancer therapy.

The utility of ENSG00000272172.1 as a predictive biomarker must be prospectively evaluated on larger patient cohorts and stratified for PARPi response. The measurement of ENSG00000272172.1 offers several advantages over current methods: (1) it can be measured using a simple real-time PCR; (2) it can be detected in low quantities in plasma samples; and (3) it can be measured in FFPE tissue samples. Further studies will also need to explore whether ENSG00000272172.1 levels in plasma reflect current HR status or past genomic events, including whether tumors that acquire PARPi resistance exhibit altered expression of this lncRNA. Such insights could guide treatment decisions in recurrent disease. Ultimately, further research and clinical trials are needed to determine the practicality and full utility of these findings.

## Methods

### Bioinformatics and data analysis

The data from the TCGA Ovarian cancer cohort (dbGaP Study Accession: phs000178.v11.p8) were extracted and downloaded from the XENA portal of the University of California, Santa Cruz (http://xena.ucsc.edu/) and the cBio-Portal website (https://www.cbioportal.org)^63^. We used RNA-seq expression, copy number variation, and mutation data. Cell line expression and drug sensitivity data were obtained from the Cancer Dependency Map (DepMap) portal (https://depmap.org), release 23Q4, which includes CCLE expression data and PRISM drug response screens^64^. lncRNA expression data from the TCGA data and the CCLE were downloaded from the TARNIC website (https://tanric.org/tanric/_design/basic/main.html), hosted by MD Anderson^65^. Genomic instability scores were used from the Publication Knijnenburg et al.^66^. BRCA aberration status in the TCGA-OV data was extracted from the publication Kraya et al.^67^. HRD-scores for the CCLE dataset were extracted from Takamatsu et al.^38,39^. Data were analyzed with the R programming language and the program GraphPad Prism. Heatmaps were created using the R package complex heatmaps^68^. To predict HRD, PARPi, or tumor type, the Tidy Models, ranger, kernlab, kknn, xgboost, glmnet, lme4, baguette, ipred, finetune, and partykit packages were used. For selecting lncRNAs, we used the following R packages: caret, randomForest, reprtree, glmnet, psych, and corrr. For dimension reduction, we used the umap package. Pathway analysis was performed using the ReactomePA, DOSE, graphite, GOSemSim, and clusterProfiler R package. All R packages are listed in Supplementary Table 6. All code and documentation are publicly available at: https://github.com/kaidob/lncRNA_HRD_PAPER_2025.git.

### Selecting of lncRNA panel

To identify lncRNAs predictive of key genomic and clinical outcomes, including *CCNE1* amplification, eCARD, HRD, and PARPi7 scores, we applied a multi-algorithm feature selection strategy using four complementary methods: Random Forest, Pearson correlation, lasso regression, and ridge regression. Random Forest importance scores, significant correlation coefficients, and regression-based coefficient weights guided feature ranking. A panel of 29 lncRNAs was curated based on recurrence across ≥2 methods, relevance to multiple outcomes, high tumor expression or survival associations, and statistical significance. Coding potential was excluded through five independent tools (PRIDE 2.0, Lee TIS, PhyloCSF, CPAT, Bazzini sORFs, Supplementary Table 2). Analyses were conducted in R using tidyverse, tidymodels, caret, randomForest, and glmnet.

### Machine learning framework

We developed machine learning models to predict HRD and PARPi7 scores in ovarian cancer using lncRNA expression profiles. A curated dataset filtered for ovarian cancer samples was used, with 29 previously identified lncRNAs selected as predictive features. Samples with missing values were excluded to ensure data quality. All analyses were conducted in R (v4.2.3) using the tidymodels framework. The dataset was split into training (60%), validation (20%), and test (20%) sets using stratified sampling based on HRD or PARPi7 scores. A consistent preprocessing pipeline was applied across all models. Each model underwent hyperparameter tuning via 10-fold cross-validation using grid search, optimizing for root mean squared error (RMSE). Model performance was evaluated using RMSE, mean absolute error (MAE), *R*², and Pearson correlation. The random forest model outperformed others and was selected for final testing. To enhance interpretability, we conducted permutation-based feature importance analysis.

### Survival analysis

Survival analyses were conducted using Kaplan–Meier estimation and Cox proportional hazards modeling to evaluate the prognostic significance of lncRNA expression groups. Kaplan–Meier survival curves were generated using the survfit function from the survival R package, stratified by lncRNA expression cluster or individual lncRNA expression, and statistical significance was assessed with the log-rank test. Survival curves and risk tables were visualized using the ggsurvplot function from the survminer package. For multivariate analysis, Cox proportional hazards models were constructed using the coxph function, adjusting for confounding variables such as age and tumor purity. Hazard ratios were extracted from the models, and the proportional hazards assumption was tested.

### Western blot and antibodies

For western blotting, cells were lysed in RIPA buffer supplemented with protease inhibitors for 30 min on ice. Proteins content was quantified by Bradford assay (Bio-Rad). Twenty microgram proteins were analyzed through a 4–15% gradient SDS-PAGE before being transferred to the PVDF membrane with the TurboBlot system (Bio-Rad). The membrane was blocked with 5% (w/v) milk in TBS-tween for one hour at room temperature before incubating in primary antibody overnight at 4 °C. All antibodies are listed in Supplementary Table 7. After washing three times with TBS-tween the membrane was incubated either with an anti-mouse or an anti-rabbit HRP-conjugated secondary antibody for 1 h. Protein bands were detected using Clarity Chemiluminescent HRP Antibody Detection Reagent (Bio-Rad) and visualized with a chemiluminescence reader (Fusion SL) imaging system.

### Cell culture and cell lines

The cell lines FT282, FT240, FT246, KURAMOCHI, OVKATE, COV318, and OVSAHO cells were a gift from Ronny Drapkin (UPenn, Philadelphia, USA). The establishment of the FT cell lines has been previously described in ref. ^69^. The ovarian cancer cell lines (OVCAR8, OVCAR3, CAOV4, and CAOV3) were obtained from American Type Culture Collection (ATCC, Manassas, VA). All cancer cell lines were cultured in DMEM F12 (Invitrogen, Carlsbad, CA) supplemented with 10% fetal bovine serum (FBS, Atlanta Biologicals) and 1% penicillin/streptomycin (Invitrogen). All cells were grown inside an incubator maintaining 37 °C and a 5% CO_2_-containing atmosphere. All cells were regularly tested for mycoplasma contamination. Human cancer cell lines were authenticated by STR profiling within the past three years using PCR-single-locus-technology (16 loci, AmpFlSTR® Identifiler® Plus, Thermo Fisher). We used the Cell Line Authentication service from Eurofins.

### Short-interfering RNA transfection

A pool of three short-interfering RNA (siRNA) duplexes was used to downregulate the expression of the lncRNA ENSG00000272172.1. The siRNA sequences CAUUGCGCACUGCACCAGUAAACAC, AGAGAACCCUAAGAAGACAGCAACA, and UGCACCAGUAAACACCUGAACACTC are targeting all three transcripts of ENSG00000272172.1. For the knock down of *BRCA1*, we used the commercially validated siRNA sc-29219 from Santa Cruz Biotechnology (Dallas, USA). As a negative control the SR30004, Trilencer-27 Universal Scrambled Negative Control (Origene) siRNA Duplex was used. Twenty-four hours after seeding 1 × 10^5^ cells in six-well plates or 2000 cells in well of a 96-well plate, the cells were transfected with the siRNA using Lipofectamine RNAiMAX (Invitrogen, Karlsruhe, Germany) together with 10 nM siRNA duplex per well as described in the instructions.

### Cell survival

Cell growth and survival were measured using a CellTiter 96® Non-Radioactive Cell Proliferation Assay at an absorbance of 570 nm using (Promega, Mannheim, Germany). Each assay was performed in triplicates and repeated at least 3 times. Data are presented by means ± SD. Statistical and significant differences were determined by ANOVA with post-hoc analysis. Cells were additionally stained with crystal violet to count the remaining attached cells.

### Immunofluorescence

Cells were grown overnight on coverslips in 96-well Cell Imaging Plates (Eppendorf). After washing, cells were fixed in 4% (v/v) paraformaldehyde in PBS for 20 min at room temperature. Cells were permeabilized and blocked with a super-block buffer (Thermo Scientific) supplemented with 0.3% Triton X-100 for 10 min. Cells were incubated with primary antibodies (Table S7) for 2 h at room temperature or overnight in the cold room. Following three washing steps the secondary antibody conjugated to Alexa Fluor Dyes (Supplementary Table 7) was incubated for 45 min at room temperature. Nuclei were stained with DAPI for 3 minutes. After three washing steps, the cells were mounted with Fluoromount-G (Sigma-Aldrich) prior to microscopy using a Zeiss Axio Observer inverted microscope with 40x magnification. For EDU proliferation analysis, we cultured the cells for 20 min with 10 µM EdU before washing and fixing with 4% PFA for 20 minutes. The cells will then be incubated with a buffer containing 100 mM Tris-buffered saline (pH 7.6), CuSO4 (4 mM), Azide-488 (2 µM), and 100 mM freshly made sodium ascorbate. After incubation for 5 min the cells are washed with PBS and co-stained with DAPI before imaging.

### Image analysis

Quantification of nuclear foci and nuclear fluorescence intensity was performed using CellProfiler (Broad Institute), an open-source image analysis software. Custom analysis pipelines were developed to identify individual nuclei and quantify foci number, size, and total fluorescence intensity within each nucleus. For each experimental condition, we analyzed images acquired from at least three different fields of view per sample, ensuring representation across the slide. A minimum of 100 nuclei per biological replicate was quantified to provide statistically robust measurements and repeated at least three times. The full CellProfiler pipeline, including input settings, is publicly available at our GitHub repository: https://github.com/kaidob/lncRNA_HRD_PAPER_2025.git.

### Fiber spread

1 × 105 cells per well were plated in each six-well. Seventy-two hours after treatment, cells were labeled with CIdU (38 µM) for 30 min, washed 3 times before medium containing 250 µM IdU was added for 30 min. Cells were trypsinized, washed, and re-suspended in 30 µl PBS. Directly on a glass slide, 2.5 µl cell suspension was mixed with 7.5 µl of lysis buffer (200 mM TrisHCl, pH 7.4, 50 mM EDTA, 0.5% SDS). Slides were tilted manually 45° to allow the drop to run slowly down the slide and air dried. The DNA was fixed in cold methanol/acetic acid (3:1 o/n) for 5 min. Rehydrated slides were denatured in 2.5 M HCl for 1 h. After washing the slides were blocked for 30 min in Superblock solution, following the incubation with the primary antibodies mouse anti-BrdU/IdU (1:100, BD Bioscience) and rat anti-BRDU/CIdU (1:200, Abcam), for overnight at 4 °C. After washing, slides were incubated with secondary fluorescent antibodies (anti-mouse alexa 488, 1:300, Molecular Probes, anti-rat Cy3, 1:300 Jackson ImmunoResearch) diluted in blocking solution for one hour. Slides were washed and air dried before mounted with coverslip and 20 µl Antifade Gold (Invitorgen). Fibers were examined using a DMRE, upright, Light Microscope Leica fluorescence microscope with a 100 × oil immersion objective, and analyzed using ImageJ software. Each assay at least 100 fibers were analyzed per condition and repeated at least 3 times.

### Cell cycle analysis

Cells were incubated with 10 µM EdU for 20–30 min, washed with PBS, and fixed in 70% ethanol at −20°C for 1 h. After washing, EdU incorporation was detected using a click chemistry reaction (10 mM 6-Carboxyfluorescein-TEG-azide, 10 mM sodium ascorbate, 2 mM CuSO₄ in PBS) for 30 min in the dark. Cells were then washed and stained with 1 µg/mL DAPI. Fluorescence was measured on a BD FACS Canto II, acquiring 10,000 single cells per sample, and analyzed using FlowJo. Initial gating was performed on forward scatter (FSC) and side scatter (SSC) to exclude debris and select the main cell population. Next, doublets were excluded by plotting DAPI width (355–450/50 linear mode) against DAPI area, and gating on the singlet population to ensure only single nuclei were analyzed. S phase cells were identified via DAPI vs. EdU dot plots, and EdU intensity was exported for individual cells (Supplementary Fig. 5b). Data were plotted in GraphPad Prism as scatter dot plots. Mean fluorescence intensity was normalized to the untreated control for each biological replicate (*n* = 3) and presented as relative expression.

### RNA isolation and real-time PCR

Total RNA was isolated using the RNeasy Plus Mini Kit (Qiagen) according to the manufacturer’s protocol. RNA quantity and purity were assessed using a NanoDrop spectrophotometer. For each sample, 0.5 µg of RNA was reverse transcribed to cDNA using the RT² Easy First Strand Kit (Qiagen). Quantitative real-time PCR (qPCR) was carried out using the PowerUp SYBR Green Master Mix (Life Technologies) and gene-specific primers (Supplementary Table 8). PCR amplification was performed on a QuantStudio Real-Time PCR System (Thermo Fisher Scientific), and Ct-values were used to calculate relative gene expression using the ΔΔCt method. All gene expression levels were normalized to GAPDH, and each experiment was performed with at least three independent biological replicates.

For RNA isolation from plasma samples, whole blood was first centrifuged at 5000 rpm for 5 min, followed by a second centrifugation at 13,000 rpm for 5 min. RNA was extracted from 200 µL of plasma supernatant using the RNeasy Plasma Kit (Qiagen). Detection of lncRNA ENSG00000272172.1 in plasma was performed using the QuantiNova SYBR Green RT-PCR Kit (Qiagen, #208152) and the corresponding QuantiNova LNA PCR Assay (Qiagen, #249990). Plasma lncRNA expression levels were normalized to plasma input volume. Due to the limited amaRNA from formalin-fixed paraffin-embedded (FFPE) tissue sections was extracted using the RNeasy FFPE Kit (Qiagen) according to the manufacturer’s instructions.

### Crispr CAS9

OVCAR8 cells were seeded in a 24-well plate 24 h prior to transfection to 80% confluency. 3pmol of the Cas9 Nuclease and 3.9pmol of each *BRCA2* sgRNAs (Sequences: UCUACCUGACCAAUCGA; UAGCACGCAUUCACAUA, were diluted in 25 µL Opti-MEM media together with 1 µL Lipofectamine Cas9 Plus reagent (Synthego CRISPR system). 1.5 µL of the Lipofectamine CRISPRMAX transfection reagent diluted in 25 µL Opti-MEM media was then mixed with the diluted nucleic acid dilution and mixed gently. The nucleic acid/transfection reagent solution was incubated at room temperature for 5 min before added to the cells. The cells were incubated for 72 h before seeding single cells to 96-well plates. Grown clones were expanded and analyzed by Western blot for the loss of *BRCA2* expression. Potential clones were sequenced to confirm cleaved sequences. We used two different clones for this study.

### Statistics and reproducibility

Statistical analyses were performed using GraphPad Prism and R. For comparisons between two groups, we used unpaired two-tailed *t*-tests. For comparisons involving more than two groups, one-way ANOVA followed by appropriate post hoc tests was applied. For correlation analyses, we used Pearson’s correlation coefficient.

All reported sample sizes (*n*) refer to biologically independent samples, as specified in each figure legend. Each experiment was repeated independently at least three times, unless stated otherwise, with similar results obtained. Details of the number of replicates, error bars, and the statistical tests used are provided in the corresponding figure legends.

### Ethics oversight

This study did not involve animal subjects. Human samples were obtained from patients undergoing surgery at the University Medical Center Mannheim. Ethical approval for the use of these samples was granted by the Ethics Committee of the Medical Faculty Mannheim, Heidelberg University (approval number 2011-380N-MA). Informed consent was obtained from all participants prior to sample collection. All ethical regulations relevant to human research participants were followed.

### Reporting summary

Further information on research design is available in the Nature Portfolio Reporting Summary linked to this article.

## Supplementary information

Supplementary Information

Description of Additional Supplementary Files

Supplementary Data File 1

Supplementary Data File 2

Reporting Summary

## Data Availability

All data used in this study are included in the Supplementary Data files or are publicly available through the TCGA and CCLE data portals. CCLE expression and drug sensitivity data are summarized in Supplementary Table 4, and TCGA ovarian cancer data with associated scores are provided in Supplementary Data File 1. Uncropped and unedited Western blot images are available in Supplementary Fig. 6. Values to generate the main figures can be found in Supplementary Data File 2.

## Code availability

All custom code and scripts used for data analysis and figure generation in this study are openly available at https://github.com/kaidob/lncRNA_HRD_PAPER_2025.git. For long-term access, the code has also been archived in Zenodo under the DOI: 10.5281/zenodo.16757894.
