# Supplementary material for: Long non-coding RNAs as a biomarker for homologous recombination deficiency and parp inhibitor sensitivity in high-grade serous ovarian cancers
Source: Commun Biol. 2025 Oct 1;8:1410. doi: 10.1038/s42003-025-08836-9 (PMC12488886; doi:10.1038/s42003-025-08836-9)
Supplement: Supplementary file 1 — Supplementary Information [file 42003_2025_8836_MOESM1_ESM.pdf]

Long Non-coding RNAs as a Biomarker for Homologous Recombination Deficiency and PARP Inhibitor Sensitivity in High-Grade Serous Cancers

Kai Doberstein[1,2,*], Johannes Panther[1], Eric Hahnen[3], Lisa Richters[3], Philip C Schouten[4], Philipp Harter[5], Florian Heitz[5,6], Stefan Kommoss[7,8,9], Sebastian Berlit[1], Benjamin Tuschy[1], Marc Sütterlin[1,2], Frederik Marmé[1,2,9,10]

[1]Department of Obstetrics and Gynecology, Medical Faculty Mannheim of the Heidelberg University, University Medical Centre Mannheim, 68167 Mannheim, Germany.
[2]Mannheim Institute for Innate Immunoscience, Medical Faculty Mannheim of the Heidelberg University, 68167 Mannheim, Germany.
[3]Center for Familial Breast and Ovarian Cancer and Center for Integrated Oncology (CIO), Cologne, Faculty of Medicine and University Hospital Cologne, Cologne, Germany;
[4]Department of histopathology, Addenbrooke's Hospital, Cambridge University Hospitals NHS Foundation Trust, cambridge, United Kingdom
[5]Department of Gynecology and Gynecologic Oncology, Evang. Kliniken Essen-Mitte, Essen, Germany.
[6]Department for Gynecology with the Center for Oncologic Surgery Charité Campus Virchow-Klinikum, Charité – Universitätsmedizin Berlin, corporate member of Freie Universität Berlin, Humboldt-Universität zu Berlin, and Berlin Institute of Health, Berlin, Germany.
[7]Department of Women's Health, Tübingen University Hospital, Tübingen, Germany.
[8]Department of Obstetrics and Gynecology, Diakonie Klinikum, Schwäbisch Hall, Germany
[9]AGO Study Group, Wiesbaden, Germany
[10]DKFZ-Hector Cancer Institute at University Medical Center Mannheim, Mannheim, Germany.

***Corresponding author:** kai.doberstein@medma.uni-heidelberg.de

Supplementary information

Supplementary Figure 1

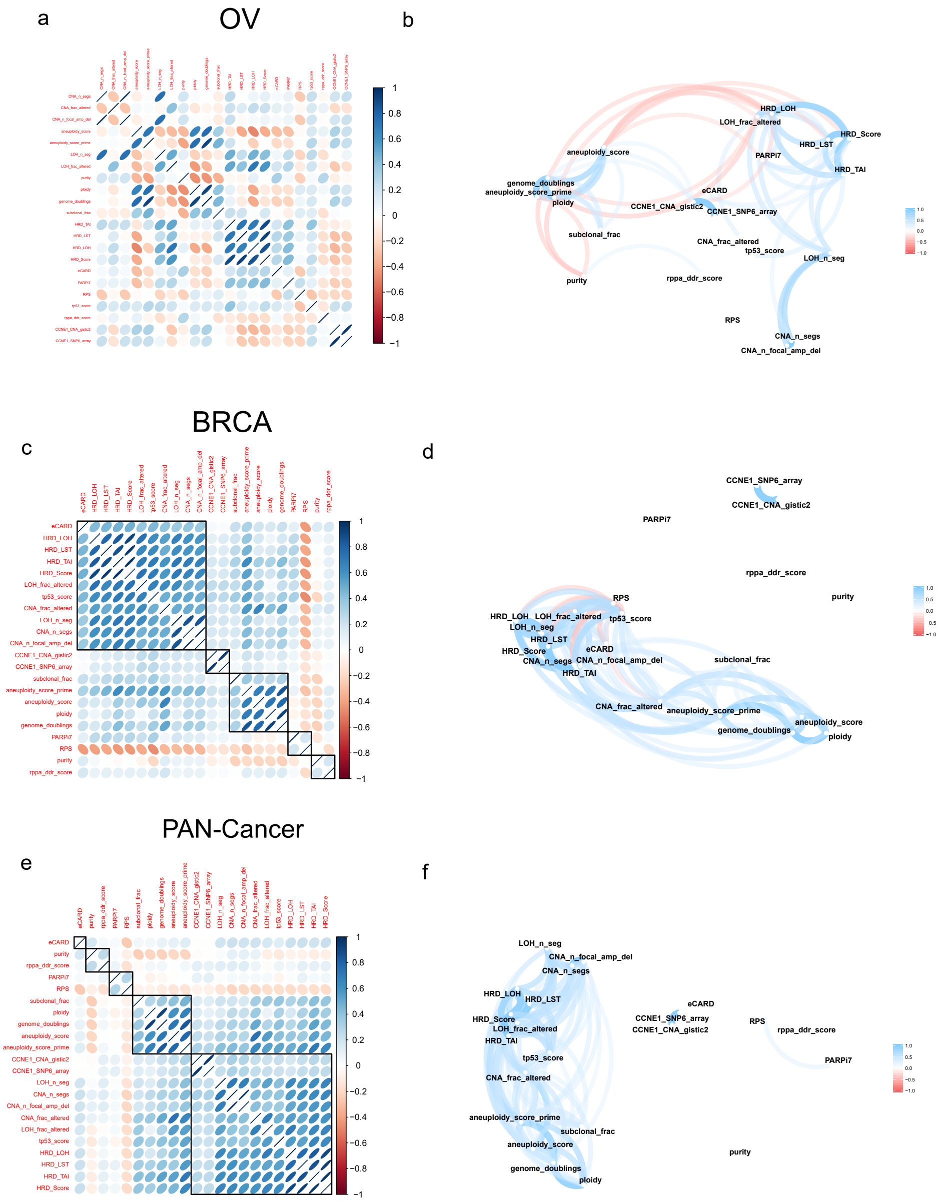

Supplementary Figure 1:

a) Pearson correlation matrix between different scores of the OV-cancer TCGA dataset. b) Correlation network based on a) between different scores in the OV-cancer TCGA dataset. c) Pearson correlation matrix between different scores of the BRCA-cancer TCGA datasets. d) Correlation network based on c) between different scores in the BRCA and PAN-cancer datasets of the TCGA data. e) Pearson correlation matrix between different scores of the BRCA-cancer TCGA datasets. f) Correlation network based on e) between different scores in the BRCA and PAN-cancer datasets of the TCGA data.

Supplementary Figure 2

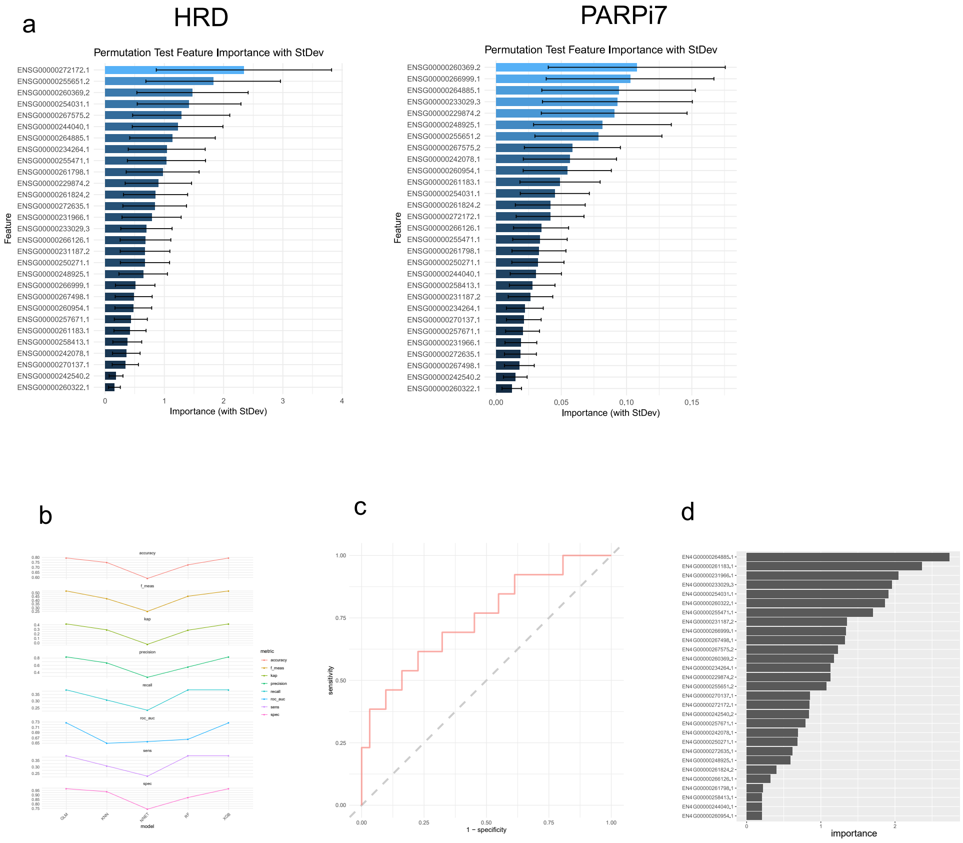

Supplementary Figure 2:

a) Feature importance analysis of the TCGA ovarian cancer dataset for predicting HRD and PARPi7 using permutation test. b) Different metrics to predict PARPi7 binary score with different models in the ovarian cancer dataset of the TCGA data. c) AUC predicting PARPi7 using GLM algorithm on the validation dataset in the ovarian cancer dataset of the TCGA data. d) Feature importance analysis to predict PARPi7 values using RF in the ovarian cancer dataset.

Supplementary Figure 3

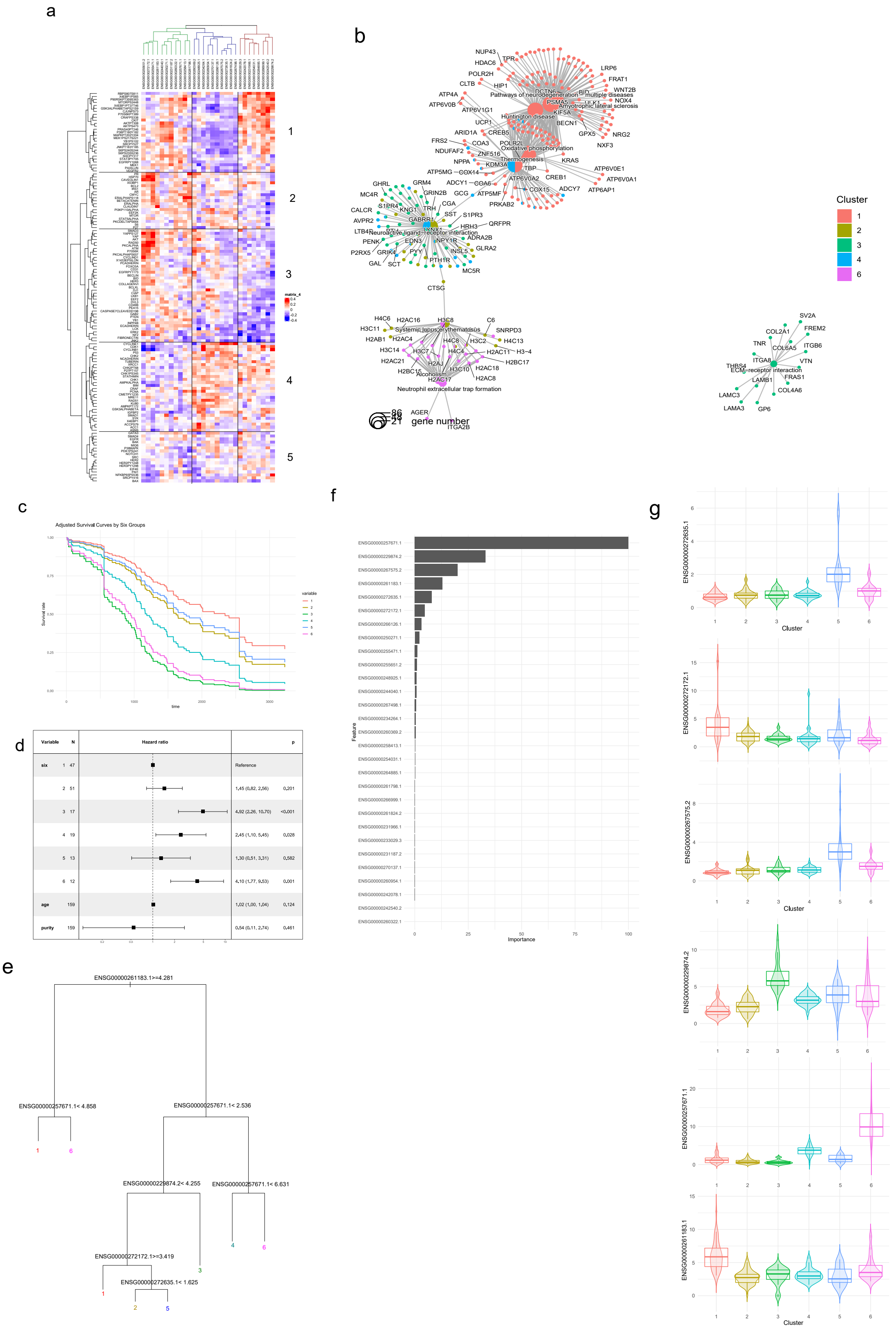

Supplementary Figure 3:

a) Pearson correlation matrix of RPPA expression with the expression of 29 lncRNAs. b) Enriched genes in each of the six cluster. c) Adjusted Kaplan-Meier survival analysis of OS and the six cluster adjusted for confounding factors age and tumor purity. d) Analysis using a multivariate Cox proportional hazard model of the six cluster from c). e and f) Feature importance analysis for each cluster using RF. g) Expression of most important lncRNAs for each cluster.

Supplementary Figure 4

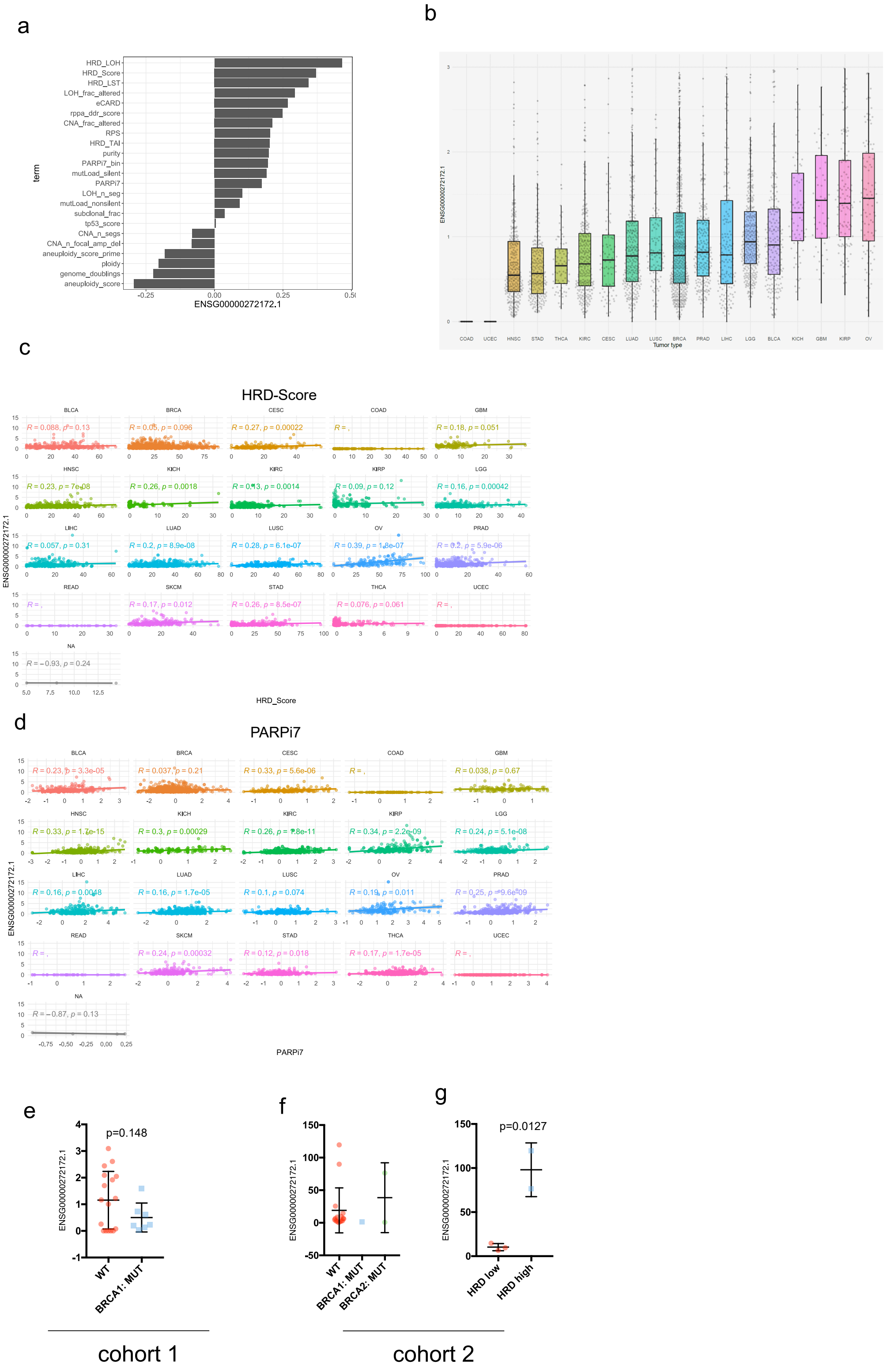

Supplementary Figure 4:

a) Pearson correlation of predetermined scores with ENSG00000272172.1 in the ovarian cancer dataset of the TCGA data. b) Expression of ENSG00000272172.1 in different tumor types of the PAN-cancer dataset of the TCGA. c) Correlation of ENSG00000272172.1 against HRD-score in different tumor types of the PAN-cancer dataset of the TCGA. d) Correlation of ENSG00000272172.1 against PARPi7-score in different tumor types of the PAN-cancer dataset of the TCGA. e) Detection of ENSG00000272172.1 in patient plasma samples of cohort 1, stratified by BRCA1/2 status (WT vs BRCA1 mutation). Expression levels were normalized to plasma volume and measured via RT-qPCR. f) Plasma expression levels of ENSG00000272172.1 in patients grouped by BRCA1/2 status: WT, BRCA1-mutated, or BRCA2-mutated in cohort 2. g) Comparison of ENSG00000272172.1 plasma expression between HRD-high and HRD-low patients in cohort 2.

Supplementary Figure 5

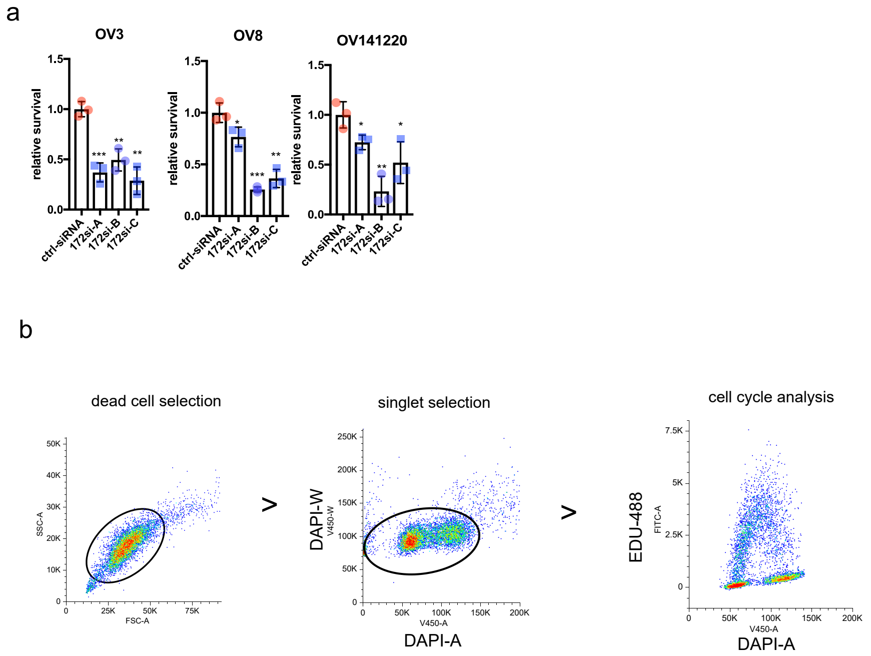

Supplementary Figure 5:

a) Relative survival of OVCAR3, OVCAR8 or OV141220 cells following the treatment with ctrl-siRNA or three different siRNAs targeting ENSG00000272172.1. b) Visualization of the FACS gating strategy for cell cycle distribution (EDU-488 and DAPI staining). The gating strategy is shown for one exemplary experiment.

Supplementary Figure 6

Figure 5f

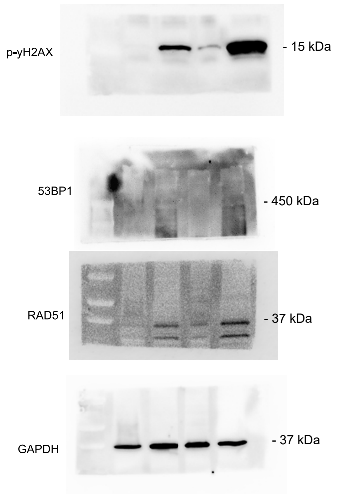

Figure 5j

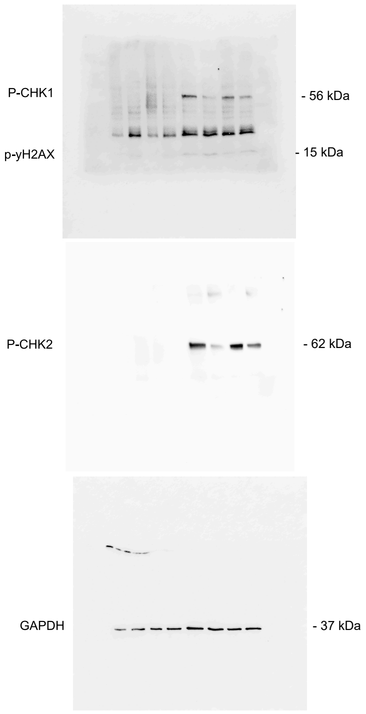

Figure 5i

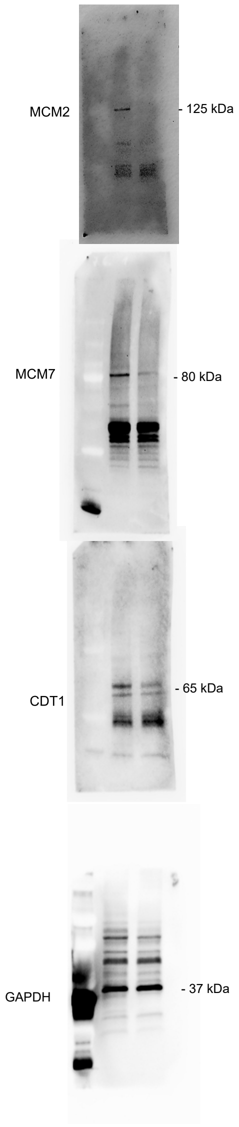

Supplementary Figure 6:

Uncropped western blots from the main manuscript.

Supplementary Table 1 | list of selected lncRNAs

Corrr_analysis	Random_Forrest	Ridge regression	lasso	all_lncRNAs	common_lncRNAs	dublicated_lncRNAs	survival	high_exp	HRD_score	CCNE1amp	PARPi	Polyplodie	eCARD	CNV	tp53	BRCA
ENSG00000235897.1	ENSG00000231754.1	ENSG00000263765.1	ENSG00000224533.3	ENSG00000253778.1	ENSG00000272172.1	ENSG00000260902.1	ENSG00000226022.1	ENSG00000269888.1	ENSG00000263765.1	ENSG00000261824.2	ENSG00000237476.1	ENSG00000233757.2	ENSG00000234129.3	ENSG00000236031.1	ENSG00000242553.1	ENSG00000261798.1
ENSG00000272172.1	ENSG00000227372.6	ENSG00000249478.1	ENSG00000230773.2	ENSG00000251161.2	ENSG00000229874.2	ENSG00000230174.1	ENSG00000227517.2	ENSG00000177410.8	ENSG00000249478.1	ENSG00000267575.2	ENSG00000255651.2	ENSG00000259623.1	ENSG00000224281.4	ENSG00000251141.1	ENSG00000234264.1	ENSG00000250271.1
ENSG00000234006.1	ENSG00000269996.1	ENSG00000261803.1	ENSG00000230174.1	ENSG00000251161.2	ENSG00000261824.2	ENSG00000261803.1	ENSG00000249662.1	ENSG00000262477.1	ENSG00000261803.1	ENSG00000272635.1	ENSG00000230438.5	ENSG00000269792.1	ENSG00000232412.1	ENSG00000271653.1	ENSG00000237594.2	ENSG00000254031.1
ENSG00000269038.1	ENSG00000266999.1	ENSG00000230174.1	ENSG00000233907.1	ENSG00000273344.1	ENSG00000267575.2	ENSG00000267498.1	ENSG00000254123.1	ENSG00000258486.2	ENSG00000230174.1	ENSG00000267498.1	ENSG00000261393.1	ENSG00000233029.3	ENSG00000230337.1	ENSG00000249279.1	ENSG00000271921.1	ENSG00000260322.1
ENSG00000260369.2	ENSG00000260743.1	ENSG00000232458.1	ENSG00000237713.1	ENSG00000234264.1	ENSG00000272635.1	ENSG00000260954.1	ENSG00000238185.1	ENSG00000251562.3	ENSG00000232458.1	ENSG00000253978.1	ENSG00000260369.2	ENSG00000232311.1	ENSG00000248925.1	ENSG00000272677.1	ENSG00000175701.6	ENSG00000244040.1
ENSG00000273344.1	ENSG00000229874.2	ENSG00000230773.2	ENSG00000241151.1	ENSG00000255651.2	ENSG00000248925.1	ENSG00000266450.1	ENSG00000265369.2	ENSG00000130600.11	ENSG00000230773.2	ENSG00000261183.1	ENSG00000254682.1	ENSG00000260978.1	ENSG00000259802.1	ENSG00000259798.1	ENSG00000271930.1	ENSG00000242078.1
ENSG00000229874.2	ENSG00000229839.2	ENSG00000167459.12	ENSG00000267006.1	ENSG00000260708.1	ENSG00000257671.1	ENSG00000267006.1	ENSG00000250271.1	ENSG00000248223.1	ENSG00000224533.3	ENSG00000255651.2	ENSG00000264885.1	ENSG00000242540.2	ENSG00000259736.1	ENSG00000272657.1	ENSG00000258884.1	ENSG00000266126.1
ENSG00000243849.1	ENSG00000272365.1	ENSG00000266893.1	ENSG00000230969.2	ENSG00000270673.1	ENSG00000231187.2	ENSG00000267575.2	ENSG00000266126.1	ENSG00000175061.13	ENSG00000230773.2	ENSG00000254610.1	ENSG00000267104.1	ENSG00000269072.1	ENSG00000227857.2	ENSG00000224939.1	ENSG00000259417.2	ENSG00000258413.1
ENSG00000272093.1	ENSG00000203739.3	ENSG00000249478.1	ENSG00000167459.12	ENSG00000255517.2	ENSG00000270137.1	ENSG00000270137.1	ENSG00000226235.1	ENSG00000269900.2	ENSG00000230174.1	ENSG00000255471.1	ENSG00000257742.1	ENSG00000260293.1	ENSG00000234264.1	ENSG00000270137.1	ENSG00000250033.1	ENSG00000242540.2
ENSG00000254031.1	ENSG00000258317.1	ENSG00000259690.1	ENSG00000249036.1	ENSG00000224961.1	ENSG00000260954.1	ENSG00000270145.1	ENSG00000272024.1	ENSG00000245910.4	ENSG00000233907.1	ENSG00000261824.2	ENSG00000233029.3	ENSG00000228714.2	ENSG00000249476.1	ENSG00000260954.1	ENSG00000253447.1	ENSG00000231966.1
ENSG00000254154.4	ENSG00000270137.1	ENSG00000267006.1	ENSG00000236226.1	ENSG00000231050.1	ENSG00000264885.1	ENSG00000273344.1	ENSG00000237686.2	ENSG00000260032.1	ENSG00000237713.1	ENSG00000267575.2	ENSG00000266999.1	ENSG00000231187.2	ENSG00000249996.1	ENSG00000229739.1	ENSG00000237457.2	
ENSG00000251161.2	ENSG00000249279.1	ENSG00000223643.2	ENSG00000273059.1	ENSG00000273117.1	ENSG00000266999.1	ENSG00000229494.1	ENSG00000254177.1	ENSG00000226950.2	ENSG00000241151.1	ENSG00000272635.1	ENSG00000225386.1	ENSG00000259448.2	ENSG00000229841.1	ENSG00000258413.1	ENSG00000257989.1	
ENSG00000237476.1	ENSG00000260954.1	ENSG00000229775.2	ENSG00000226759.3	ENSG00000234134.1	ENSG00000234129.3	ENSG00000234264.1	ENSG00000236464.1	ENSG00000269972.1	ENSG00000267709.1	ENSG00000267498.1	ENSG00000234426.1	ENSG00000253404.1	ENSG00000257671.1	ENSG00000247287.2	ENSG00000228528.1	
ENSG00000255651.2	ENSG00000259843.2	ENSG00000236678.3	ENSG00000232132.1	ENSG00000232065.1	ENSG00000272288.1	ENSG00000249478.1	ENSG00000256994.1	ENSG00000259001.2	ENSG00000260962.1	ENSG00000261183.1	ENSG00000225278.3	ENSG00000254109.1	ENSG00000257671.1	ENSG00000250072.1	ENSG00000264727.1	
ENSG00000230438.5	ENSG00000261589.1	ENSG00000236445.2	ENSG00000225166.1	ENSG00000272288.1	ENSG00000175701.6	ENSG00000251161.2	ENSG00000257947.1	ENSG00000204272.6	ENSG00000260466.1	ENSG00000272172.1	ENSG00000205041.1	ENSG00000261183.1	ENSG00000268093.1	ENSG00000241577.1	ENSG00000224032.2	
ENSG00000261393.1	ENSG00000260331.1	ENSG00000229981.3	ENSG00000236355.2	ENSG00000245468.3	ENSG00000224032.2	ENSG00000259690.1	ENSG00000245688.1	ENSG00000270170.1	ENSG00000254563.1	ENSG00000263627.1	ENSG00000228980.3	ENSG00000270604.1	ENSG00000248925.1	ENSG00000245573.3	ENSG00000254115.1	
ENSG00000260369.2	ENSG00000257671.1	ENSG00000250754.1	ENSG00000250141.1	ENSG00000235897.1	ENSG00000254682.1	ENSG00000261183.1	ENSG00000227486.1	ENSG00000232388.2	ENSG00000226425.1	ENSG00000253982.1	ENSG00000243715.1	ENSG00000250938.1	ENSG00000246523.3	ENSG00000253978.1	ENSG00000214353.3	
ENSG00000254682.1	ENSG00000268093.1	ENSG00000232132.1	ENSG00000236355.2	ENSG00000272172.1	ENSG00000269996.1	ENSG00000261824.2	ENSG00000263677.1	ENSG00000269893.2	ENSG00000259730.1	ENSG00000255471.1	ENSG00000232542.1	ENSG00000262454.1	ENSG00000237857.2	ENSG00000248019.2	ENSG00000253778.1	
ENSG00000264885.1	ENSG00000248925.1	ENSG00000260322.1	ENSG00000228750.1	ENSG00000234006.1	ENSG00000273117.1	ENSG00000263765.1	ENSG00000273313.1	ENSG00000203875.6	ENSG00000248810.1	ENSG00000196972.6	ENSG00000231754.1	ENSG00000234494.3	ENSG00000223714.1	ENSG00000235246.1	ENSG00000257517.1	
ENSG00000267104.1	ENSG00000246523.3	ENSG00000236678.3	ENSG00000225979.1	ENSG00000269038.1	ENSG00000203362.2	ENSG00000266893.1	ENSG00000266903.1	ENSG00000234741.3	ENSG00000267626.1	ENSG00000255983.1	ENSG00000227372.6	ENSG00000250604.1	ENSG00000260648.2	ENSG00000261123.1	ENSG00000230728.1	
ENSG00000257742.1	ENSG00000237857.2	ENSG00000267320.1	ENSG00000256560.1	ENSG00000260369.2	ENSG00000267498.1	ENSG00000272635.1	ENSG00000261475.2	ENSG00000269987.1	ENSG00000248138.1	ENSG00000246084.2	ENSG00000269996.1	ENSG00000223784.1	ENSG00000261803.1	ENSG00000259933.2	ENSG00000255651.2	
ENSG00000233029.3	ENSG00000223714.1	ENSG00000260902.1	ENSG00000265125.1	ENSG00000273344.1	ENSG00000266450.1	ENSG00000167459.12	ENSG00000246084.2	ENSG00000260260.1	ENSG00000253524.1	ENSG00000225386.1	ENSG00000266999.1	ENSG00000249236.1	ENSG00000228309.2	ENSG00000262117.1	ENSG00000251144.1	
ENSG00000266999.1	ENSG00000255399.2	ENSG00000260986.1	ENSG00000229282.1	ENSG00000229874.2	ENSG00000261183.1	ENSG00000175701.6	ENSG00000248935.1	ENSG00000225733.1	ENSG00000226287.3	ENSG00000250334.1	ENSG00000260743.1	ENSG00000256146.1	ENSG00000258779.2	ENSG00000258274.1	ENSG00000224825.2	
ENSG00000234264.1	ENSG00000224842.2	ENSG00000259690.1	ENSG00000241151.1	ENSG00000243849.1		ENSG00000203362.2	ENSG00000249885.1	ENSG00000253352.4	ENSG00000273139.1	ENSG00000248663.2	ENSG00000229874.2	ENSG00000233431.1	ENSG00000259998.1	ENSG00000227292.1	ENSG00000231963.1	
ENSG00000237594.2	ENSG00000253563.2	ENSG00000261446.1	ENSG00000229990.2	ENSG00000272093.1		ENSG00000223643.2	ENSG00000272209.1	ENSG00000240567.1	ENSG00000260924.2	ENSG00000234426.1	ENSG00000229839.2	ENSG00000236495.1	ENSG00000230174.1	ENSG00000240032.1	ENSG00000234393.1	
ENSG00000271921.1	ENSG00000234918.1	ENSG00000260648.2	ENSG00000229702.1	ENSG00000254031.1		ENSG00000224032.2	ENSG00000254746.1	ENSG00000126005.11	ENSG00000272172.1	ENSG00000255325.2	ENSG00000272365.1	ENSG00000248605.1	ENSG00000231481.1	ENSG00000245322.2	ENSG00000226383.1	
ENSG00000175701.6	ENSG00000235687.4	ENSG00000261803.1	ENSG00000229494.1	ENSG00000254154.4		ENSG00000224842.2	ENSG00000261629.1	ENSG00000267449.1	ENSG00000259222.1	ENSG00000225278.3	ENSG00000203739.3	ENSG00000230345.1	ENSG00000229494.1	ENSG00000251141.1	ENSG00000260303.1	
ENSG00000271930.1	ENSG00000259104.2	ENSG00000253311.1	ENSG00000251127.2	ENSG00000251161.2		ENSG00000224939.1	ENSG00000253369.1	ENSG00000270726.1	ENSG00000261447.1	ENSG00000205041.1	ENSG00000258317.1	ENSG00000239921.2	ENSG00000250754.1	ENSG00000236031.1	ENSG00000242741.1	
ENSG00000258884.1	ENSG00000272635.1	ENSG00000235881.2	ENSG00000250274.1	ENSG00000261824.2		ENSG00000226954.1	ENSG00000253314.1	ENSG00000242125.2	ENSG00000255651.2	ENSG00000272732.1	ENSG00000260322.1	ENSG00000261803.1	ENSG00000253139.1	ENSG00000272677.1	ENSG00000257004.1	
ENSG00000228528.1	ENSG00000267575.2	ENSG00000260902.1	ENSG00000239921.2	ENSG00000267575.2		ENSG00000229739.1	ENSG00000267546.2	ENSG00000250742.1	ENSG00000260708.1	ENSG00000272635.1	ENSG00000236678.3	ENSG00000232084.1	ENSG00000253297.1	ENSG00000272657.1	ENSG00000233008.1	
ENSG00000264727.1	ENSG00000267498.1	ENSG00000249236.1	ENSG00000261803.1	ENSG00000272635.1		ENSG00000229874.2	ENSG00000272764.1	ENSG00000273432.1	ENSG00000270673.1	ENSG00000267575.2	ENSG00000267320.1	ENSG00000229494.1	ENSG00000260515.1	ENSG00000249279.1	ENSG00000272724.1	
ENSG00000224032.2	ENSG00000261824.2	ENSG00000256146.1	ENSG00000232084.1	ENSG00000267498.1		ENSG00000230768.3	ENSG00000234026.1	ENSG00000272430.1	ENSG00000255517.2	ENSG00000267498.1	ENSG00000260902.1	ENSG00000261432.1		ENSG00000259798.1	ENSG00000235770.1	
ENSG00000254115.1	ENSG00000267640.1	ENSG00000233431.1	ENSG00000229494.1	ENSG00000255471.1		ENSG00000230773.2	ENSG00000203362.2	ENSG00000214783.5	ENSG00000224961.1	ENSG00000261824.2	ENSG00000260986.1	ENSG00000234015.1		ENSG00000271653.1	ENSG00000256268.1	
ENSG00000214353.3	ENSG00000267219.1	ENSG00000236495.1	ENSG00000261432.1	ENSG00000196972.6		ENSG00000230969.2	ENSG00000255382.1	ENSG00000255717.2	ENSG00000231050.1	ENSG00000267640.1	ENSG00000259690.1			ENSG00000224939.1	ENSG00000253343.1	
ENSG00000253778.1	ENSG00000253563.2	ENSG00000248605.1	ENSG00000234015.1	ENSG00000261824.2		ENSG00000231187.2	ENSG00000269758.1	ENSG00000232677.2	ENSG00000273117.1	ENSG00000267219.1	ENSG00000236355.2			ENSG00000270137.1	ENSG00000253774.1	
ENSG00000257517.1	ENSG00000255399.2	ENSG00000230345.1	ENSG00000231481.1	ENSG00000267575.2		ENSG00000231607.4	ENSG00000249196.2	ENSG00000224032.2	ENSG00000234134.1	ENSG00000167459.12	ENSG00000228750.1			ENSG00000260954.1	ENSG00000261446.1	
ENSG00000272677.1	ENSG00000223808.1	ENSG00000260648.2	ENSG00000229494.1	ENSG00000272635.1		ENSG00000232084.1	ENSG00000233338.1	ENSG00000234608.3	ENSG00000232065.1	ENSG00000266893.1	ENSG00000225979.1			ENSG00000258891.1	ENSG00000260648.2	
ENSG00000259798.1	ENSG00000258844.1	ENSG00000261803.1	ENSG00000250754.1	ENSG00000267498.1		ENSG00000232132.1	ENSG00000225316.1	ENSG00000272888.1	ENSG00000272288.1	ENSG00000249478.1	ENSG00000256560.1			ENSG00000261672.1	ENSG00000261803.1	
ENSG00000272657.1	ENSG00000234918.1	ENSG00000228309.2	ENSG00000253139.1	ENSG00000258057.1		ENSG00000234129.3	ENSG00000250313.2	ENSG00000272913.1	ENSG00000245468.3	ENSG00000259690.1	ENSG00000265125.1			ENSG00000239415.1	ENSG00000253311.1	
ENSG00000224939.1	ENSG00000224842.2	ENSG00000258779.2	ENSG00000253297.1	ENSG00000259488.1		ENSG00000234918.1	ENSG00000232749.1	ENSG00000163597.10	ENSG00000235897.1	ENSG00000267006.1	ENSG00000229282.1			ENSG00000261167.1	ENSG00000235881.2	
ENSG00000270137.1	ENSG00000267605.1	ENSG00000259998.1	ENSG00000260515.1	ENSG00000235910.1		ENSG00000236355.2	ENSG00000263427.1	ENSG00000261512.2	ENSG00000272172.1	ENSG00000223643.2	ENSG00000229775.2			ENSG00000242861.1	ENSG00000260902.1	
ENSG00000260954.1	ENSG00000227292.1	ENSG00000230174.1	ENSG00000236673.1	ENSG00000262099.1		ENSG00000236673.1	ENSG00000226953.3	ENSG00000243004.1	ENSG00000234006.1	ENSG00000267006.1	ENSG00000236678.3			ENSG00000251615.3		
ENSG00000229739.1	ENSG00000258404.1	ENSG00000260902.1	ENSG00000226954.1	ENSG00000245248.3		ENSG00000236678.3	ENSG00000264269.1	ENSG00000245060.2	ENSG00000269038.1	ENSG00000230969.2	ENSG00000236445.2			ENSG00000258999.1		
ENSG00000258413.1	ENSG00000214546.3	ENSG00000230174.1	ENSG00000266450.1	ENSG00000229720.1		ENSG00000239921.2	ENSG00000227094.1	ENSG00000232533.1	ENSG00000260369.2	ENSG00000167459.12	ENSG00000229981.3			ENSG00000247287.2		
ENSG00000247287.2	ENSG00000248859.2	ENSG00000253199.1	ENSG00000250634.1	ENSG00000227964.1		ENSG00000241151.1	ENSG00000238195.1	ENSG00000261373.1	ENSG00000273344.1	ENSG00000249036.1	ENSG00000250754.1			ENSG00000229739.1		
ENSG00000250072.1	ENSG00000258791.3	ENSG00000230768.3	ENSG00000260902.1	ENSG00000255465.3		ENSG00000241577.1	ENSG00000248279.1	ENSG00000255198.3	ENSG00000236933.1	ENSG00000236226.1	ENSG00000232132.1			ENSG00000258413.1		
ENSG00000241577.1	ENSG00000270604.1	ENSG00000233048.1	ENSG00000224865.3	ENSG00000230838.1		ENSG00000245573.3	ENSG00000239508.1	ENSG00000253859.2	ENSG00000257509.1	ENSG00000273059.1	ENSG00000226759.3			ENSG00000250072.1		
ENSG00000245573.3	ENSG00000250938.1	ENSG00000263765.1	ENSG00000266450.1	ENSG00000233117.2		ENSG00000248925.1	ENSG00000272626.1	ENSG00000267100.1	ENSG00000237990.2	ENSG00000249478.1	ENSG00000232132.1			ENSG00000241577.1		
ENSG00000253978.1	ENSG00000262454.1	ENSG00000249478.1	ENSG00000236673.1	ENSG00000248925.1		ENSG00000249036.1	ENSG00000258826.1	ENSG00000245532.4	ENSG00000255310.2	ENSG00000259690.1	ENSG00000225166.1			ENSG00000245573.3		
ENSG00000235246.1	ENSG00000234494.3	ENSG00000259690.1	ENSG00000250634.1	ENSG00000259802.1		ENSG00000250072.1	ENSG00000251642.1	ENSG00000182165.13	ENSG00000267582.1	ENSG00000258784.1	ENSG00000236355.2			ENSG00000270137.1		
ENSG00000261123.1	ENSG00000250604.1	ENSG00000258784.1	ENSG00000226954.1	ENSG00000259736.1		ENSG00000250634.1	ENSG00000230120.1	ENSG00000254635.1	ENSG00000242611.1	ENSG00000223643.2	ENSG00000250141.1			ENSG00000249279.1		
ENSG00000259933.2	ENSG00000223784.1	ENSG00000223643.2	ENSG00000254519.1	ENSG00000227857.2		ENSG00000250754.1	ENSG00000234929.1	ENSG00000263934.2	ENSG00000257681.1	ENSG00000266893.1	ENSG00000264885.1			ENSG00000260954.1		
ENSG00000262117.1	ENSG00000226287.3	ENSG00000266893.1	ENSG00000234129.3	ENSG00000234264.1		ENSG00000253199.1	ENSG00000249309.1	ENSG00000166770.6	ENSG00000269427.1	ENSG00000267006.1	ENSG00000254438.1			ENSG00000259843.2		
ENSG00000258274.1	ENSG00000273139.1	ENSG00000267006.1	ENSG00000267006.1	ENSG00000249476.1		ENSG00000253404.1	ENSG00000259834.1	ENSG00000233137.2	ENSG00000229874.2	ENSG00000267006.1	ENSG00000267128.1			ENSG00000261589.1		
ENSG00000227292.1	ENSG00000260924.2	ENSG00000260902.1	ENSG00000230969.2	ENSG00000249996.1		ENSG00000253563.2	ENSG00000231073.1	ENSG00000256234.1	ENSG00000243849.1	ENSG00000230969.2	ENSG00000259230.1			ENSG00000260331.1		
ENSG00000249279.1	ENSG00000272172.1	ENSG00000230174.1	ENSG00000249036.1	ENSG00000229841.1		ENSG00000253778.1	ENSG00000233754.1	ENSG00000259187.1	ENSG00000272093.1	ENSG00000249036.1	ENSG00000260710.1			ENSG00000267605.1		
ENSG00000259798.1	ENSG00000259222.1	ENSG00000263765.1	ENSG00000236389.1	ENSG00000257671.1		ENSG00000254682.1	ENSG00000235337.1	ENSG00000233901.1	ENSG00000254031.1	ENSG00000236389.1	ENSG00000259814.1			ENSG00000227292.1		
ENSG00000271653.1	ENSG00000261447.1	ENSG00000253199.1	ENSG00000226921.2	ENSG00000260978.1		ENSG00000255399.2	ENSG00000257582.1	ENSG00000224189.2	ENSG00000254154.4	ENSG00000226921.2	ENSG00000269086.2			ENSG00000258404.1		
ENSG00000224939.1	ENSG00000264885.1	ENSG00000230768.3	ENSG00000267498.1	ENSG00000242540.2		ENSG00000255651.2	ENSG00000259284.1	ENSG00000235106.4	ENSG00000251161.2	ENSG00000267498.1	ENSG00000225760.1			ENSG00000214546.3		
ENSG00000270137.1	ENSG00000254438.1	ENSG00000266893.1		ENSG00000269072.1		ENSG00000257671.1	ENSG00000235785.1	ENSG00000272933.1		ENSG00000236528.1	ENSG00000254615.2			ENSG00000248859.2		
ENSG00000260954.1	ENSG00000267128.1	ENSG00000261803.1		ENSG00000260293.1		ENSG00000258413.1	ENSG00000226659.1	ENSG00000218510.3		ENSG00000267381.1	ENSG00000224992.1			ENSG00000258791.3		
ENSG00000247287.2	ENSG00000259230.1	ENSG00000260902.1		ENSG00000228714.2		ENSG00000259153.1	ENSG00000236471.1	ENSG00000226363.3		ENSG00000226797.1	ENSG00000230156.2			ENSG00000270137.1		
ENSG00000229739.1	ENSG00000260710.1	ENSG00000260322.1		ENSG00000231187.2		ENSG00000259798.1	ENSG00000270178.1	ENSG00000237512.2		ENSG00000230366.5	ENSG00000185837.3			ENSG00000260954.1		
ENSG00000258413.1	ENSG00000259814.1	ENSG00000239921.2		ENSG00000259448.2		ENSG00000259843.2	ENSG00000266579.1	ENSG00000251022.2		ENSG00000272172.1	ENSG00000272620.1			ENSG00000259843.2		
ENSG00000250072.1	ENSG00000270137.1	ENSG00000235389.2		ENSG00000253404.1		ENSG00000260322.1	ENSG00000272086.1	ENSG00000225973.3		ENSG00000267219.1	ENSG00000241520.1			ENSG00000185904.7		
ENSG00000241577.1	ENSG00000260954.1	ENSG00000232084.1		ENSG00000254109.1		ENSG00000260331.1	ENSG00000253661.1	ENSG00000270066.2		ENSG00000260361.1	ENSG00000230489.1			ENSG00000259534.1		
ENSG00000245573.3	ENSG00000259843.2			ENSG00000261183.1		ENSG00000260369.2	ENSG00000237343.1	ENSG00000237149.4		ENSG00000267014.1	ENSG00000259005.1			ENSG00000260331.1		
ENSG00000232311.1	ENSG00000185904.7			ENSG00000259798.1		ENSG00000260648.2	ENSG00000255440.1	ENSG00000257084.1		ENSG00000223392.1	ENSG00000223960.2					
ENSG00000260978.1	ENSG00000259534.1			ENSG00000271653.1		ENSG00000264885.1	ENSG00000273019.1	ENSG00000206337.6		ENSG00000245573.3	ENSG00000228150.1					
ENSG00000242540.2	ENSG00000260331.1			ENSG00000224939.1		ENSG00000266999.1	ENSG00000235572.1	ENSG00000215039.2		ENSG00000272172.1	ENSG00000228544.1					
ENSG00000269072.1	ENSG00000233008.1			ENSG00000270137.1		ENSG00000267104.1	ENSG00000251543.1	ENSG00000255135.3		ENSG00000271948.1	ENSG00000235997.2					
ENSG00000260293.1	ENSG00000272724.1			ENSG00000260954.1		ENSG00000269416.1	ENSG00000230857.2	ENSG00000215256.3		ENSG00000267219.1						
ENSG00000228714.2	ENSG00000235770.1			ENSG00000229739.1		ENSG00000269996.1	ENSG00000227061.1	ENSG00000247228.2		ENSG00000248554.1						
ENSG00000231187.2	ENSG00000256268.1			ENSG00000258413.1		ENSG00000272172.1	ENSG00000229961.1	ENSG00000269996.1		ENSG00000267381.1						
ENSG00000259448.2	ENSG00000253343.1			ENSG00000250072.1		ENSG00000272288.1	ENSG00000237327.1	ENSG00000248275.1		ENSG00000230366.5						
ENSG00000253404.1	ENSG00000253774.1			ENSG00000241577.1		ENSG00000273117.1	ENSG00000249550.2	ENSG00000233016.2		ENSG00000273032.1						
ENSG00000254109.1	ENSG00000267575.2			ENSG00000245573.3			ENSG00000272243.1	ENSG00000236850.4		ENSG00000267575.2						
ENSG00000261183.1	ENSG00000267498.1			ENSG00000259798.1			ENSG00000203886.4	ENSG00000233461.1		ENSG00000259881.1						

ENSG00000230337.1	ENSG00000231560.1			ENSG00000272657.1			ENSG00000260038.1	ENSG00000236618.2		ENSG00000266904.1					
ENSG00000248925.1	ENSG00000266450.1			ENSG00000224939.1			ENSG00000231422.2	ENSG00000260552.1							
ENSG00000259802.1	ENSG00000267745.1			ENSG00000270137.1			ENSG00000262213.1	ENSG00000238142.1							
ENSG00000259736.1	ENSG00000251179.1			ENSG00000260954.1			ENSG00000265671.1	ENSG00000234171.1							
ENSG00000227857.2	ENSG00000272068.1			ENSG00000229739.1			ENSG00000232693.2	ENSG00000272288.1							
ENSG00000234264.1	ENSG00000233725.3			ENSG00000258413.1			ENSG00000238057.4	ENSG00000175701.6							
ENSG00000249476.1	ENSG00000229118.1			ENSG00000247287.2			ENSG00000245857.2	ENSG00000234678.1							
ENSG00000249996.1	ENSG00000273409.1			ENSG00000250072.1			ENSG00000269951.1	ENSG00000233368.2							
ENSG00000229841.1	ENSG00000239453.1			ENSG00000241577.1			ENSG00000254434.1	ENSG00000254682.1							
ENSG00000257671.1	ENSG00000267701.1			ENSG00000245573.3			ENSG00000253643.1	ENSG00000247556.2							
ENSG00000225411.2	ENSG00000261183.1			ENSG00000237594.2			ENSG00000260484.1	ENSG00000248008.2							
ENSG00000258057.1	ENSG00000231187.2			ENSG00000271921.1			ENSG00000257156.1	ENSG00000215808.2							
ENSG00000259488.1	ENSG00000228274.3			ENSG00000175701.6			ENSG00000249509.1	ENSG00000196756.7							
ENSG00000235910.1	ENSG00000237015.1			ENSG00000271930.1			ENSG00000257346.1	ENSG00000273344.1							
ENSG00000262099.1	ENSG00000253642.1			ENSG00000258884.1			ENSG00000251075.1	ENSG00000170846.11							
ENSG00000245248.3	ENSG00000205634.2			ENSG00000224032.2			ENSG00000273344.1	ENSG00000235437.3							
ENSG00000227248.1	ENSG00000236390.1			ENSG00000254115.1			ENSG00000255733.1	ENSG00000255026.1							
ENSG00000229720.1	ENSG00000258172.1			ENSG00000214353.3			ENSG00000226004.1	ENSG00000261183.1							
ENSG00000227964.1	ENSG00000262265.1			ENSG00000253778.1			ENSG00000273117.1	ENSG00000270081.1							
ENSG00000255465.3	ENSG00000270060.1			ENSG00000257517.1			ENSG00000228412.2	ENSG00000254615.2							
ENSG00000230838.1	ENSG00000267904.1			ENSG00000255651.2			ENSG00000230972.1	ENSG00000251580.1							
ENSG00000233117.2	ENSG00000261642.1			ENSG00000230438.5			ENSG00000236145.1	ENSG00000260336.1							
ENSG00000223960.2	ENSG00000265478.1			ENSG00000261393.1											
ENSG00000270145.1	ENSG00000255750.1			ENSG00000260369.2											
ENSG00000231607.4	ENSG00000225721.1			ENSG00000254682.1											
ENSG00000269416.1	ENSG00000263120.1			ENSG00000264885.1											
ENSG00000234129.3	ENSG00000271717.1			ENSG00000267104.1											
ENSG00000266999.1	ENSG00000223561.2			ENSG00000257742.1											
ENSG00000270145.1				ENSG00000233029.3											
ENSG00000266450.1				ENSG00000223960.2											
ENSG00000259623.1				ENSG00000270145.1											
ENSG00000267080.1				ENSG00000231607.4											
ENSG00000234506.1				ENSG00000269416.1											
ENSG00000253404.1				ENSG00000234129.3											
ENSG00000268081.1				ENSG00000266999.1											
ENSG00000250413.1				ENSG00000270145.1											
ENSG00000226272.1				ENSG00000266450.1											
ENSG00000224738.1				ENSG00000259623.1											
ENSG00000263470.1				ENSG00000267080.1											
ENSG00000264920.1				ENSG00000234506.1											
ENSG00000267104.1				ENSG00000253404.1											
ENSG00000270145.1				ENSG00000268081.1											
ENSG00000259153.1				ENSG00000250413.1											
ENSG00000259038.1				ENSG00000226272.1											
ENSG00000259146.2				ENSG00000224738.1											
ENSG00000259153.1				ENSG00000263470.1											
ENSG00000270145.1				ENSG00000264920.1											
ENSG00000269416.1				ENSG00000267104.1											
ENSG00000231607.4				ENSG00000270145.1											
ENSG00000203362.2				ENSG00000259153.1											
ENSG00000234264.1				ENSG00000259038.1											
ENSG00000261824.2				ENSG00000259146.2											
ENSG00000267575.2				ENSG00000259153.1											
ENSG00000272635.1				ENSG00000270145.1											
ENSG00000267498.1				ENSG00000269416.1											
ENSG00000253978.1				ENSG00000231607.4											
ENSG00000261183.1				ENSG00000203362.2											
				ENSG00000234264.1											
				ENSG00000231754.1											
				ENSG00000227372.6											
				ENSG00000269996.1											
				ENSG00000266999.1											
				ENSG00000260743.1											
				ENSG00000229874.2											
				ENSG00000229839.2											
				ENSG00000272365.1											
				ENSG00000203739.3											
				ENSG00000258317.1											
				ENSG00000270137.1											
				ENSG00000249279.1											
				ENSG00000260954.1											
				ENSG00000259843.2											
				ENSG00000261589.1											
				ENSG00000260331.1											
				ENSG00000257671.1											
				ENSG00000268093.1											
				ENSG00000248925.1											
				ENSG00000246523.3											
				ENSG00000237857.2											
				ENSG00000223714.1											
				ENSG00000255399.2											
				ENSG00000224842.2											
				ENSG00000253563.2											
				ENSG00000234918.1											

				ENSG00000235687.4											
				ENSG00000259104.2											
				ENSG00000272635.1											
				ENSG00000267575.2											
				ENSG00000267498.1											
				ENSG00000261824.2											
				ENSG00000267640.1											
				ENSG00000267219.1											
				ENSG00000253563.2											
				ENSG00000255399.2											
				ENSG00000223808.1											
				ENSG00000258844.1											
				ENSG00000234918.1											
				ENSG00000224842.2											
				ENSG00000267605.1											
				ENSG00000227292.1											
				ENSG00000258404.1											
				ENSG00000214546.3											
				ENSG00000248859.2											
				ENSG00000258791.3											
				ENSG00000270604.1											
				ENSG00000250938.1											
				ENSG00000262454.1											
				ENSG00000234494.3											
				ENSG00000250604.1											
				ENSG00000223784.1											
				ENSG00000226287.3											
				ENSG00000273139.1											
				ENSG00000260924.2											
				ENSG00000272172.1											
				ENSG00000259222.1											
				ENSG00000261447.1											
				ENSG00000264885.1											
				ENSG00000254438.1											
				ENSG00000267128.1											
				ENSG00000259230.1											
				ENSG00000260710.1											
				ENSG00000259814.1											
				ENSG00000270137.1											
				ENSG00000260954.1											
				ENSG00000259843.2											
				ENSG00000185904.7											
				ENSG00000259534.1											
				ENSG00000260331.1											
				ENSG00000233008.1											
				ENSG00000272724.1											
				ENSG00000235770.1											
				ENSG00000256268.1											
				ENSG00000253343.1											
				ENSG00000253774.1											
				ENSG00000267575.2											
				ENSG00000267498.1											
				ENSG00000231560.1											
				ENSG00000266450.1											
				ENSG00000267745.1											
				ENSG00000251179.1											
				ENSG00000272068.1											
				ENSG00000233725.3											
				ENSG00000229118.1											
				ENSG00000273409.1											
				ENSG00000239453.1											
				ENSG00000267701.1											
				ENSG00000261183.1											
				ENSG00000231187.2											
				ENSG00000228274.3											
				ENSG00000237015.1											
				ENSG00000253642.1											
				ENSG00000205634.2											
				ENSG00000236390.1											
				ENSG00000258172.1											
				ENSG00000262265.1											
				ENSG00000270060.1											
				ENSG00000267904.1											
				ENSG00000261642.1											
				ENSG00000265478.1											
				ENSG00000255750.1											
				ENSG00000225721.1											
				ENSG00000263120.1											
				ENSG00000271717.1											
				ENSG00000223561.2											
				ENSG00000263765.1											
				ENSG00000249478.1											
				ENSG00000261803.1											
				ENSG00000230174.1											

				ENSG00000232458.1											
				ENSG00000230773.2											
				ENSG00000224533.3											
				ENSG00000230773.2											
				ENSG00000230174.1											
				ENSG00000233907.1											
				ENSG00000237713.1											
				ENSG00000241151.1											
				ENSG00000167459.12											
				ENSG00000266893.1											
				ENSG00000249478.1											
				ENSG00000259690.1											
				ENSG00000267006.1											
				ENSG00000223643.2											
				ENSG00000267006.1											
				ENSG00000230969.2											
				ENSG00000167459.12											
				ENSG00000249036.1											
				ENSG00000236226.1											
				ENSG00000273059.1											
				ENSG00000229775.2											
				ENSG00000236678.3											
				ENSG00000236445.2											
				ENSG00000229981.3											
				ENSG00000250754.1											
				ENSG00000232132.1											
				ENSG00000226759.3											
				ENSG00000232132.1											
				ENSG00000225166.1											
				ENSG00000236355.2											
				ENSG00000250141.1											
				ENSG00000260322.1											
				ENSG00000236678.3											
				ENSG00000267320.1											
				ENSG00000260902.1											
				ENSG00000260986.1											
				ENSG00000259690.1											
				ENSG00000236355.2											
				ENSG00000228750.1											
				ENSG00000225979.1											
				ENSG00000256560.1											
				ENSG00000265125.1											
				ENSG00000229282.1											
				ENSG00000261446.1											
				ENSG00000260648.2											
				ENSG00000261803.1											
				ENSG00000253311.1											
				ENSG00000235881.2											
				ENSG00000260902.1											
				ENSG00000241151.1											
				ENSG00000229990.2											
				ENSG00000229702.1											
				ENSG00000229494.1											
				ENSG00000251127.2											
				ENSG00000250274.1											
				ENSG00000249236.1											
				ENSG00000256146.1											
				ENSG00000233431.1											
				ENSG00000236495.1											
				ENSG00000248605.1											
				ENSG00000230345.1											
				ENSG00000239921.2											
				ENSG00000261803.1											
				ENSG00000232084.1											
				ENSG00000229494.1											
				ENSG00000261432.1											
				ENSG00000234015.1											
				ENSG00000260648.2											
				ENSG00000261803.1											
				ENSG00000228309.2											
				ENSG00000258779.2											
				ENSG00000259998.1											
				ENSG00000230174.1											
				ENSG00000231481.1											
				ENSG00000229494.1											
				ENSG00000250754.1											
				ENSG00000253139.1											
				ENSG00000253297.1											
				ENSG00000260515.1											
				ENSG00000260902.1											
				ENSG00000230174.1											
				ENSG00000253199.1											
				ENSG00000230768.3											
				ENSG00000233048.1											

				ENSG00000263765.1											
				ENSG00000236673.1											
				ENSG00000226954.1											
				ENSG00000266450.1											
				ENSG00000250634.1											
				ENSG00000260902.1											
				ENSG00000224865.3											
				ENSG00000260902.1											
				ENSG00000230174.1											
				ENSG00000263765.1											
				ENSG00000253199.1											
				ENSG00000230768.3											
				ENSG00000266893.1											
				ENSG00000266450.1											
				ENSG00000236673.1											
				ENSG00000250634.1											
				ENSG00000226954.1											
				ENSG00000254519.1											
				ENSG00000234129.3											
				ENSG00000261803.1											
				ENSG00000260902.1											
				ENSG00000260322.1											
				ENSG00000239921.2											
				ENSG00000235389.2											
				ENSG00000232084.1											
				ENSG00000249478.1											
				ENSG00000259690.1											
				ENSG00000258784.1											
				ENSG00000223643.2											
				ENSG00000266893.1											
				ENSG00000267006.1											
				ENSG00000267006.1											
				ENSG00000230969.2											
				ENSG00000249036.1											
				ENSG00000236389.1											
				ENSG00000226921.2											
				ENSG00000267498.1											
				ENSG00000269888.1											
				ENSG00000177410.8											
				ENSG00000262477.1											
				ENSG00000258486.2											
				ENSG00000251562.3											
				ENSG00000130600.11											
				ENSG00000248223.1											
				ENSG00000175061.13											
				ENSG00000269900.2											
				ENSG00000245910.4											
				ENSG00000260032.1											
				ENSG00000226950.2											
				ENSG00000269972.1											
				ENSG00000259001.2											
				ENSG00000204272.6											
				ENSG00000270170.1											
				ENSG00000232388.2											
				ENSG00000269893.2											
				ENSG00000203875.6											
				ENSG00000234741.3											
				ENSG00000269987.1											
				ENSG00000260260.1											
				ENSG00000225733.1											
				ENSG00000253352.4											
				ENSG00000240567.1											
				ENSG00000126005.11											
				ENSG00000267449.1											
				ENSG00000270726.1											
				ENSG00000242125.2											
				ENSG00000250742.1											
				ENSG00000273432.1											
				ENSG00000272430.1											
				ENSG00000214783.5											
				ENSG00000255717.2											
				ENSG00000232677.2											
				ENSG00000224032.2											
				ENSG00000234608.3											
				ENSG00000272888.1											
				ENSG00000272913.1											
				ENSG00000163597.10											
				ENSG00000261512.2											
				ENSG00000243004.1											
				ENSG00000245060.2											
				ENSG00000232533.1											
				ENSG00000261373.1											
				ENSG00000255198.3											
				ENSG00000253859.2											

				ENSG00000267100.1											
				ENSG00000245532.4											
				ENSG00000182165.13											
				ENSG00000254635.1											
				ENSG00000263934.2											
				ENSG00000166770.6											
				ENSG00000233137.2											
				ENSG00000256234.1											
				ENSG00000259187.1											
				ENSG00000233901.1											
				ENSG00000224189.2											
				ENSG00000235106.4											
				ENSG00000272933.1											
				ENSG00000218510.3											
				ENSG00000226363.3											
				ENSG00000237512.2											
				ENSG00000251022.2											
				ENSG00000225973.3											
				ENSG00000270066.2											
				ENSG00000237149.4											
				ENSG00000257084.1											
				ENSG00000206337.6											
				ENSG00000215039.2											
				ENSG00000255135.3											
				ENSG00000215256.3											
				ENSG00000247228.2											
				ENSG00000269996.1											
				ENSG00000248275.1											
				ENSG00000233016.2											
				ENSG00000236850.4											
				ENSG00000233461.1											
				ENSG00000236618.2											
				ENSG00000260552.1											
				ENSG00000238142.1											
				ENSG00000234171.1											
				ENSG00000272288.1											
				ENSG00000175701.6											
				ENSG00000234678.1											
				ENSG00000233368.2											
				ENSG00000254682.1											
				ENSG00000247556.2											
				ENSG00000248008.2											
				ENSG00000215808.2											
				ENSG00000196756.7											
				ENSG00000273344.1											
				ENSG00000170846.11											
				ENSG00000235437.3											
				ENSG00000255026.1											
				ENSG00000261183.1											
				ENSG00000270081.1											
				ENSG00000254615.2											
				ENSG00000251580.1											
				ENSG00000260336.1											
				ENSG00000226022.1											
				ENSG00000227517.2											
				ENSG00000249662.1											
				ENSG00000254123.1											
				ENSG00000238185.1											
				ENSG00000265369.2											
				ENSG00000250271.1											
				ENSG00000266126.1											
				ENSG00000226235.1											
				ENSG00000272024.1											
				ENSG00000237686.2											
				ENSG00000254177.1											
				ENSG00000236464.1											
				ENSG00000256994.1											
				ENSG00000257947.1											
				ENSG00000245688.1											
				ENSG00000227486.1											
				ENSG00000263677.1											
				ENSG00000273313.1											
				ENSG00000266903.1											
				ENSG00000261475.2											
				ENSG00000246084.2											
				ENSG00000248935.1											
				ENSG00000249885.1											
				ENSG00000272209.1											
				ENSG00000254746.1											
				ENSG00000261629.1											
				ENSG00000253369.1											
				ENSG00000253314.1											
				ENSG00000267546.2											
				ENSG00000272764.1											

				ENSG00000234026.1											
				ENSG00000203362.2											
				ENSG00000255382.1											
				ENSG00000269758.1											
				ENSG00000249196.2											
				ENSG00000233338.1											
				ENSG00000225316.1											
				ENSG00000250313.2											
				ENSG00000232749.1											
				ENSG00000263427.1											
				ENSG00000226953.3											
				ENSG00000264269.1											
				ENSG00000227094.1											
				ENSG00000238195.1											
				ENSG00000248279.1											
				ENSG00000239508.1											
				ENSG00000272626.1											
				ENSG00000258826.1											
				ENSG00000251642.1											
				ENSG00000230120.1											
				ENSG00000234929.1											
				ENSG00000249309.1											
				ENSG00000259834.1											
				ENSG00000231073.1											
				ENSG00000233754.1											
				ENSG00000235337.1											
				ENSG00000257582.1											
				ENSG00000259284.1											
				ENSG00000235785.1											
				ENSG00000226659.1											
				ENSG00000236471.1											
				ENSG00000270178.1											
				ENSG00000266579.1											
				ENSG00000272086.1											
				ENSG00000253661.1											
				ENSG00000237343.1											
				ENSG00000255440.1											
				ENSG00000273019.1											
				ENSG00000235572.1											
				ENSG00000251543.1											
				ENSG00000230857.2											
				ENSG00000227061.1											
				ENSG00000229961.1											
				ENSG00000237327.1											
				ENSG00000249550.2											
				ENSG00000272243.1											
				ENSG00000203886.4											
				ENSG00000260038.1											
				ENSG00000231422.2											
				ENSG00000262213.1											
				ENSG00000265671.1											
				ENSG00000232693.2											
				ENSG00000238057.4											
				ENSG00000245857.2											
				ENSG00000269951.1											
				ENSG00000254434.1											
				ENSG00000253643.1											
				ENSG00000260484.1											
				ENSG00000257156.1											
				ENSG00000249509.1											
				ENSG00000257346.1											
				ENSG00000251075.1											
				ENSG00000273344.1											
				ENSG00000255733.1											
				ENSG00000226004.1											
				ENSG00000273117.1											
				ENSG00000228412.2											
				ENSG00000230972.1											
				ENSG00000236145.1											
				ENSG00000237476.1											
				ENSG00000228528.1											
				ENSG00000264727.1											
				ENSG00000272677.1											
				ENSG00000253978.1											
				ENSG00000235246.1											
				ENSG00000261123.1											
				ENSG00000259933.2											
				ENSG00000262117.1											
				ENSG00000258274.1											
				ENSG00000232311.1											
				ENSG00000230337.1											
				ENSG00000225411.2											
				ENSG00000227248.1											

Supplementary Table 2 | Selected lncRNA information

ensembl_gene_id	Transcript ID	LNCipedia gene ID:	Alternative gene names	Alternative gene names	Alternative gene names	Chromosomal Location	Class	Sequence Ontology term	gene_biotype	PRIDE reprocessing 2.0	Lee translation initiation sites	PhyloCSF score	CPAT coding probability	Bazzini small ORFs	close genes (http://genome.ucsc.edu/)
ENSG00000229874	ENSG00000229874.2	lnc-RHEX-3	RP11-312O7.2			chr1:206135293-206137984	sense-overlapping	sense_overlap_ncRNA	lncRNA	non-coding	non-coding	non-coding	non-coding	non-coding	SRGAP2 FAM72A
ENSG00000231187	ENSG00000231187.2	lnc-GPRIN2-1	RP11-38L15.3	SYT15-AS1		chr10:46951472-46966835	antisense	antisense_lncRNA	lncRNA	non-coding	non-coding	non-coding	non-coding	non-coding	PTPN20
ENSG00000231966	ENSG00000231966.1	lnc-TOR1AIP2-1	RP11-12M5.4	LINC02818	LINC02818	chr1:179829609-179836124	intergenic	lincRNA	lncRNA	non-coding	non-coding	non-coding	non-coding	non-coding	piR-43107-025 HSALNG0008779
ENSG00000233029	ENSG00000233029.3	lnc-FAM72B-17	RP11-439A17.9	lnc-FAM72B-17:1	AC244453.2	chr1:121090289-121097655	antisense	antisense_lncRNA	lncRNA	non-coding	non-coding	non-coding	non-coding	non-coding	H3P4 FCGR1BP
ENSG00000234264	ENSG00000234264.1	DEPDC1-AS1	RP4-694A7.4			chr1:68496676-68538627	antisense	antisense_lncRNA	lncRNA	non-coding	non-coding	non-coding	non-coding	non-coding	DEPDC1 TXNP2
ENSG00000242078	ENSG00000242078.1	lnc-UBE2H-1	AC084864.1	AC084864.1	RP11-738B7.1	chr7:129783370-129785185	intergenic	lincRNA	lncRNA	non-coding	non-coding	non-coding	non-coding	non-coding	UBE2H MIR183 MIR182
ENSG00000242540	ENSG00000242540.2	lnc-SOX11-1	AC010729.2	AC010729.2	ENST00000455579.2	chr2:5696220-5719670	intronic	sense_intronic_ncRNA	lncRNA	non-coding	non-coding	non-coding	non-coding	non-coding	SOX11
ENSG00000244040	ENSG00000244040.1	IL12A-AS1	IL12A-AS1	MICT00000253474.1		chr3:159902045-160225299	intergenic	lincRNA	lncRNA	non-coding	non-coding	non-coding	non-coding	non-coding	IL12A
ENSG00000248925	ENSG00000248925.1	lnc-CCDC127-1	CTD-2083E4.6	XLOC_004692	AC021087.1	chr5:269973-271631	bidirectional		lncRNA	non-coding	non-coding	non-coding	non-coding	non-coding	PDCD6
ENSG00000250271	ENSG00000250271.1	lnc-AADAC-1	RP11-64D22.5	OTTHUMG00000162581.1		chr3:151515272-151526037	intergenic	lincRNA	transcribed_unprocessed_pseudogene	non-coding	non-coding	non-coding	non-coding	non-coding	IGSF10
ENSG00000254031	ENSG00000254031.1	lnc-XKR9-2	RP11-326E22.1	AC022858.1	OTTHUMG00000164432.1	chr8:71155454-71204223	intergenic	lincRNA	lncRNA	non-coding	non-coding	non-coding	non-coding	non-coding	EYA1
ENSG00000255471	ENSG00000255471.1	lnc-FZD4-1	RP11-736K20.5	PRSS23-AS1		chr11:86603256-86636079	antisense	antisense_lncRNA	lncRNA	non-coding	non-coding	non-coding	non-coding	non-coding	ME3
ENSG00000255651	ENSG00000255651.2	lnc-C11orf95-1	RP11-466C23.4			chr11:63767344-63768641	intronic	sense_intronic_ncRNA	lncRNA	non-coding	non-coding	non-coding	non-coding	non-coding	ZFTA
ENSG00000257671	ENSG00000257671.1	KRT7-AS	RP3-416H24.1			chr12:52245048-52247448	antisense	antisense_lncRNA	lncRNA	non-coding	non-coding	non-coding	non-coding	non-coding	KRT7
ENSG00000258413	ENSG00000258413.1	lnc-DLGAP5-4	RP11-665C16.6	AL158801.2	OTTHUMG00000171032.1	chr14:55262222-55272075	antisense	antisense_lncRNA	lncRNA	non-coding	non-coding	non-coding	non-coding	non-coding	FBXO34
ENSG00000260322	ENSG00000260322.1	lnc-ADGRL2-3	RP11-339A11.2	AC098657.2		chr1:80580628-80582603	intergenic	lincRNA	lncRNA	non-coding	non-coding	non-coding	non-coding	non-coding	
ENSG00000260369	ENSG00000260369.2	lnc-NPTX1-1	CTD-2526A2.2	lnc-NPTX1-1	AC120024.1	chr17:80453735-80454729	intergenic	lincRNA	lncRNA	non-coding	non-coding	non-coding	non-coding	non-coding	NPTX1
ENSG00000260954	ENSG00000260954.1	lnc-PTX4-2	lnc-PTX4–2	lnc-PTX4-2:1	OTTHUMG00000176603.1	chr16:1579242-1580308	intronic	sense_intronic_ncRNA	lncRNA	non-coding	non-coding	non-coding	non-coding	non-coding	IFT140
ENSG00000261183	ENSG00000261183.1	SPINT1-AS1	RP11-532F12.5	SPINT1-AS1:20	OTTHUMG00000172899.1	chr15:40835993-40844387	antisense	antisense_lncRNA	lncRNA	non-coding	non-coding	non-coding	non-coding	non-coding	SPINT1
ENSG00000261824	ENSG00000261824.2	LINC00662	LINC00662	linc-ZNF681-4		chr19:28175488-28284848	intergenic	lincRNA	lncRNA	non-coding	non-coding	non-coding	non-coding	coding	
ENSG00000264885	ENSG00000264885.1	lnc-TBC1D28-1	RP11-815I9.4	lnc-TBC1D28-1:1	OTTHUMG00000179787.1	chr17:18667629-18669461	intronic	sense_intronic_ncRNA	lncRNA	non-coding	non-coding	non-coding	non-coding	non-coding	ZNF286B FOXO3B
ENSG00000266126	ENSG00000266126.1	lnc-ULK2-1	RP11-209D14.4	OTTHUMG00000178491.1		chr17:19929372-19929737	intronic	sense_intronic_ncRNA	lncRNA	non-coding	non-coding	non-coding	non-coding	non-coding	AKAP10
ENSG00000266999	ENSG00000266999.1	lnc-RDM1-1	AC015849.16	lnc-RDM1-1:4		chr17:35893707-35911023	intergenic	lincRNA	lncRNA	non-coding	non-coding	non-coding	coding	non-coding	CCL5 RDM1
ENSG00000267498	ENSG00000267498.1	lnc-VSTM2B-5	CTB-32O4.2	UQCRFS1-DT		chr19:29704142-29706722	antisense	antisense_lncRNA	lncRNA	non-coding	non-coding	non-coding	non-coding	non-coding	PLEKHF1
ENSG00000267575	ENSG00000267575.2	lnc-VSTM2B-9	CTC-459F4.3	lnc-VSTM2B-9	AC006504.5	chr19:28284371-28409771	intergenic	lincRNA	lncRNA	non-coding	non-coding	non-coding	non-coding	non-coding	CCNE1 POP4 VSTM2B NRF1
ENSG00000270137	ENSG00000270137.1	lnc-TG-2	CTC-137K3.1	lnc-TG-2:1	AF230666.2	chr8:132826179-132826903	intronic	sense_intronic_ncRNA	lncRNA	non-coding	non-coding	non-coding	non-coding	non-coding	PHF20L1
ENSG00000270977	ENSG00000270977.1	lnc-RDM1-1	AC015849.16	AC015849.5		chr17:34220711-34238027	intergenic	lincRNA	transcribed_processed_pseudogene	non-coding	non-coding	non-coding	coding	non-coding	CCL2
ENSG00000272172	ENSG00000272172.1	lnc-TOP1MT-4	RP13-582O9.7	lnc-TOP1MT-4:2	AC138696.2	chr8:143290399-143290621	antisense	antisense_lncRNA	lncRNA	non-coding	non-coding	non-coding	non-coding	non-coding	ZNF696
ENSG00000272635	ENSG00000272635.1	lnc-VSTM2B-9	LLNLF-65H9.1	lnc-VSTM2B-9:8		chr19:28284396-28475892	intergenic	lincRNA	lncRNA	non-coding	non-coding	non-coding	coding	non-coding	CCNE1 POP4 VSTM2B NRF1

Supplementary Table 3 | TCGA-OV patient cluster

1	2	3	4	5	6
TCGA-04-1348	TCGA-04-1357	TCGA-09-0364	TCGA-09-1670	TCGA-13-0916	TCGA-13-1403
TCGA-04-1362	TCGA-09-1673	TCGA-09-1674	TCGA-20-1682	TCGA-13-2060	TCGA-13-1511
TCGA-09-0369	TCGA-13-0800	TCGA-09-2044	TCGA-23-1114	TCGA-20-1684	TCGA-23-1111
TCGA-09-2051	TCGA-13-0890	TCGA-10-0928	TCGA-23-1122	TCGA-23-1123	TCGA-24-1418
TCGA-13-0893	TCGA-13-0899	TCGA-13-1506	TCGA-23-2084	TCGA-24-1424	TCGA-24-1474
TCGA-13-0897	TCGA-13-1489	TCGA-20-1683	TCGA-24-1413	TCGA-24-2019	TCGA-24-2033
TCGA-13-0913	TCGA-13-1489	TCGA-20-1685	TCGA-24-1417	TCGA-25-1329	TCGA-24-2288
TCGA-13-0913	TCGA-13-1505	TCGA-23-1809	TCGA-24-1436	TCGA-29-1695	TCGA-24-2298
TCGA-13-0920	TCGA-13-1507	TCGA-24-1419	TCGA-24-1469	TCGA-29-1703	TCGA-25-1312
TCGA-13-1405	TCGA-23-1029	TCGA-24-1552	TCGA-24-1562	TCGA-29-1761	TCGA-25-2399
TCGA-13-1481	TCGA-23-2077	TCGA-24-1564	TCGA-24-1842	TCGA-57-1994	TCGA-29-1710
TCGA-13-1497	TCGA-24-1416	TCGA-25-1315	TCGA-24-2254	TCGA-59-2348	TCGA-29-1710
TCGA-13-1498	TCGA-24-1423	TCGA-25-1316	TCGA-24-2261	TCGA-61-1733	TCGA-30-1862
TCGA-13-1512	TCGA-24-1430	TCGA-25-2396	TCGA-24-2267	TCGA-61-1741	TCGA-57-1993
TCGA-20-1687	TCGA-24-1467	TCGA-25-2404	TCGA-24-2271	TCGA-61-2012	TCGA-61-1736
TCGA-23-1027	TCGA-24-1550	TCGA-29-1697	TCGA-24-2281		TCGA-61-1910
TCGA-23-2081	TCGA-24-1603	TCGA-29-1702	TCGA-24-2290		TCGA-61-1995
TCGA-24-1103	TCGA-24-1616	TCGA-59-2350	TCGA-25-1314		TCGA-61-2088
TCGA-24-1427	TCGA-24-1843	TCGA-59-2352	TCGA-25-1323		
TCGA-24-1428	TCGA-24-1844	TCGA-61-2102	TCGA-25-2392		
TCGA-24-1551	TCGA-24-1846		TCGA-29-1778		
TCGA-24-1847	TCGA-24-1850		TCGA-31-1953		
TCGA-24-2024	TCGA-24-2035		TCGA-61-2094		
TCGA-24-2262	TCGA-25-1319		TCGA-61-2104		
TCGA-25-1313	TCGA-25-2393		TCGA-61-2109		
TCGA-25-2391	TCGA-25-2400		TCGA-61-2110		
TCGA-25-2397	TCGA-29-1691		TCGA-61-2113		
TCGA-29-1688	TCGA-29-1699				
TCGA-29-1693	TCGA-29-1701				
TCGA-29-1694	TCGA-29-1705				
TCGA-29-1696	TCGA-29-1705				
TCGA-29-1711	TCGA-29-1762				
TCGA-29-1770	TCGA-29-1763				
TCGA-29-1770	TCGA-29-1781				
TCGA-29-1776	TCGA-29-1783				
TCGA-29-1784	TCGA-29-2427				
TCGA-29-1785	TCGA-30-1855				
TCGA-30-1718	TCGA-30-1891				
TCGA-61-1725	TCGA-59-2351				
TCGA-61-1738	TCGA-59-2355				
TCGA-61-1740	TCGA-59-2363				
TCGA-61-1907	TCGA-61-1728				
TCGA-61-1914	TCGA-61-1900				
	TCGA-61-1919				
	TCGA-61-1998				
	TCGA-61-2000				
	TCGA-61-2008				
	TCGA-61-2008				
	TCGA-61-2009				
	TCGA-61-2016				
	TCGA-61-2092				
	TCGA-61-2111				

Supplementary Table 4 | CCLE lncRNA expression

	ENSG0000(	ENSG0000(	ENSG0000(	ENSG0000(	ENSG0000(	ENSG0000(	ENSG0000(	ENSG0000(	ENSG0000(	ENSG0000(	ENSG0000(	ENSG0000(	ENSG0000(	ENSG0000(	ENSG0000(	ENSG0000(	ENSG0000(	ENSG0000(	ENSG0000(	ENSG0000(	ENSG0000(	ENSG0000(	ENSG0000(	ENSG0000(	ENSG0000(	ENSG0000(	ENSG0000(	ENSG0000(	ENSG0000(	HRD_score	lineage_sub
OV56	0.953125	0.821495	0.889406	-1.596787	-1.795437	0.677698	-0.648242	0.466864	-3.202717	-1.357804	-0.687409	1.248527	0.126248	0.11601	0.741453	-0.229459	0.604978	-0.186994	-0.056196	-0.027767	0.459234	0.613793	0.262285	-2.230409	-1.965683	0.600561	0.718371	-0.247262	0.385004	22	serous
COV644	-0.609895	-0.066786	-2.470309	0.63948	-2.39945	0.061218	-0.648242	-1.500192	-3.202717	0.696747	-0.687409	-1.090723	-3.242446	0.271139	-5.230178	-0.229459	-2.732623	0.331593	0.445623	-0.020687	-0.827139	-1.695886	-1.755531	-0.954941	-1.965683	-0.91683	-2.070699	-1.788139	-0.426441	37	mucinous
MCAS	-0.233963	0.708597	-0.32555	0.652195	-0.912995	0.141957	-0.648242	0.360924	-0.20816	1.294071	-0.687409	-1.085546	-0.532987	0.772077	-0.30686	-0.229459	0.640968	-0.500315	0.957568	0.400571	-3.512257	0.233759	-1.755531	-1.548959	-1.965683	-3.378074	0.266623	-1.469573	-5.125312	29	mucinous
RMUG-S	0.33372	1.24615	0.207148	0.589006	1.10038	0.459449	-0.648242	0.749857	-3.202717	-1.357804	1.134063	-0.06413	-0.069577	0.751735	0.308003	-0.229459	0.432525	0.648252	0.999484	0.834058	-2.216402	-0.556837	0.635035	1.252269	0.503662	-2.119954	0.413603	1.218139	-1.485401	27	mucinous
JHOM-1	0.511594	1.932132	0.254208	-1.596787	-0.46616	0.563527	-0.648242	0.275922	0.603101	1.417016	1.065149	0.658452	0.463825	0.596796	0.316831	-0.229459	0.372291	0.073343	-0.920428	-0.085694	-0.277123	0.766438	0.686687	-1.23395	0.186342	0.089352	0.465783	-0.086509	0.0761	26	mucinous
JHOM-2B	-1.022412	-2.177457	-2.470309	0.102761	0.222429	0.364201	-0.648242	-1.500192	0.302776	0.987137	-0.687409	-0.387119	0.350373	-0.161169	0.951998	-0.229459	0.230829	-0.06058	1.129708	0.924488	-0.099316	0.818021	0.198563	-0.713648	0.607481	0.091447	0.410484	1.115218	0.026793	19	mucinous
OV7	0.439687	-0.143007	0.084449	-1.596787	0.935718	-0.012373	1.742118	0.539634	0.427251	0.429177	-0.687409	-0.349506	-3.242446	-2.930488	-0.383663	-0.229459	-0.084836	-0.116903	-0.975531	-0.274922	0.250607	-0.1368	-1.755531	-0.413639	-1.965683	0.483234	-2.070699	-1.50094	0.422556	16	mixed_sero
IGROV1	-0.187287	0.214598	0.162667	-1.596787	0.322723	0.201671	-0.648242	0.840248	0.373065	-1.357804	-0.687409	1.325478	0.518403	-1.022173	0.27534	-0.229459	-2.732623	0.480118	-0.017812	0.24095	-0.038625	0.265341	0.773662	0.400243	0.132069	0.166871	0.256437	0.108551	0.195286	3	mixed_end
Hey-A8	-0.520327	-1.675435	-2.470309	0.469371	0.821951	0.08729	-0.648242	1.200571	0.512875	1.114646	-0.687409	0.170519	0.031747	0.361441	-0.764145	-0.229459	-2.732623	0.548957	-0.785024	0.578214	0.206037	-0.82844	0.210278	-1.576817	-0.321407	0.013927	-2.070699	0.084046	0.105162	14	low_grade_
Caov-3	-0.583403	0.546414	-0.0101	0.824808	0.54876	0.266851	-0.648242	-1.500192	0.517987	0.614308	1.182161	0.027314	0.210895	0.598697	-0.849776	-0.229459	0.492258	-5.495937	0.817219	0.377078	-0.196043	-1.108465	0.41103	0.215523	0.474918	-0.05364	0.558503	-0.337441	-0.048311	88	high_grade
OVCAR-8	0.374088	0.450709	0.341451	0.524662	0.782282	-0.087015	-0.648242	0.653867	0.39888	0.068615	1.900605	0.010405	0.397623	0.51562	0.246649	-0.229459	-0.400501	0.723349	-0.680704	0.3774	0.016851	0.266393	0.156673	-0.750078	-1.965683	-0.009643	0.5874	0.237938	0.212919	88	high_grade
SNU-119	1.211736	0.108004	0.551393	1.149036	0.905779	0.190317	1.029261	-1.500192	0.307632	-1.357804	1.495609	0.357894	0.422133	0.764663	0.757343	-0.229459	0.328303	-0.407278	0.76188	-0.315471	0.333584	-0.246284	0.359378	0.236095	0.963756	0.270056	0.629394	3.009948	0.265492	87	high_grade
JHOS-4	0.307228	-0.204327	0.420099	0.495379	-0.238627	-0.7323	-0.648242	0.645367	0.437475	0.127377	-0.687409	1.353774	0.555547	0.548699	0.187061	-0.229459	0.704451	0.549792	1.088262	0.220997	0.090818	-0.475778	1.026422	0.504389	0.895678	0.182584	0.827099	1.51514	0.304676	83	high_grade
OAW28	-0.878598	-1.206652	0.581692	1.230912	0.222429	0.293975	1.57397	0.787589	0.489361	-1.357804	-0.687409	0.656382	0.231109	0.392429	0.772792	-0.229459	0.706201	-0.355545	0.878445	0.709515	0.10362	0.791703	-1.755531	1.357272	0.585734	0.31772	0.573887	0.554543	0.273982	76	high_grade
OVCAR-4	-0.567004	1.293143	0.722441	0.361296	1.035264	0.789345	1.360891	0.671904	-0.070905	-1.357804	1.601304	-0.431978	-0.093834	0.842988	0.33228	-0.229459	0.217332	-0.260422	0.998072	0.444016	1.02537	-0.882129	-1.755531	0.409243	0.553775	0.803264	0.422542	0.300671	0.562314	74	high_grade
COV362	0.746236	-0.637005	0.021059	-1.596787	0.336944	-2.424887	-0.648242	-1.500192	-0.494427	0.25831	1.264615	0.590128	-0.166605	0.39433	0.657146	-0.229459	0.276816	0.527262	0.359671	0.137003	0.187071	-0.55789	0.52037	-0.072486	0.408352	0.032783	0.163509	1.226961	0.283125	72	high_grade
FU-OV-1	0.256767	-1.070257	0.720077	0.512525	-0.341915	0.6224	1.487563	0.132456	0.474025	-0.280823	1.260776	0.493162	0.33875	-0.310975	-0.271107	-0.229459	0.125857	0.571069	-1.665268	-0.246602	1.171409	0.337979	0.855844	1.097978	1.197302	1.499368	0.535635	-0.220797	1.025017	51	high_grade
OVSAHO	0.322366	0.483375	0.35198	0.160555	0.071987	0.852003	-0.648242	0.669831	0.25089	0.846791	-0.687409	0.377218	-0.04153	0.761811	0.98422	-0.229459	0.726695	-1.009723	0.275131	0.766476	0.467294	-0.862128	0.355828	0.144378	0.790913	0.956208	0.526488	-0.500154	0.671377	50	high_grade
59M	0.393011	0.764186	0.684191	0.151308	0.494122	-2.424887	-0.648242	0.075236	0.34316	1.028784	-0.687409	-0.146948	0.035789	0.28863	0.098782	-0.229459	0.260071	-0.143604	-0.837066	-0.032916	0.557857	0.325346	0.428248	-0.780936	0.517088	0.096161	-2.070699	-0.227658	0.168837	47	high_grade
ONCO-DG-	-1.055211	1.189988	0.556765	0.858907	0.014355	0.660036	1.831636	1.06063	0.411148	0.464549	0.937021	0.053884	0.384989	0.677783	0.897265	-0.229459	1.032613	0.252741	0.757876	0.523827	0.280005	0.858025	0.696982	1.096264	0.335357	0.105589	0.810467	-0.014954	-0.037209	45	high_grade
KURAMOC	0.227752	0.007142	0.60361	0.56088	-0.319461	0.572148	0.824477	0.250422	-0.227329	0.438306	1.913741	0.869292	0.673547	0.015253	-0.516523	-0.229459	-2.732623	0.097958	-0.05078	-0.880259	0.520873	0.453778	0.788217	-0.012484	0.59708	0.670748	0.786768	0.310473	0.499292	42	high_grade
NIH_OVCA	-0.336146	0.404289	0.373684	1.013026	-0.082197	0.408566	1.716714	1.243901	0.213829	0.690043	1.271284	-0.017546	0.697551	0.667707	0.556067	-0.229459	0.806174	0.384578	0.772477	0.184632	0.139182	0.739067	0.938737	1.046548	0.156842	-0.02588	0.690306	-0.935364	-0.03427	42	high_grade
COV318	0.823189	0.014592	0.825585	0.740621	1.53	0.520003	-0.648242	1.082813	0.548659	0.420762	1.631214	0.300612	-3.242446	0.297184	0.999227	-0.229459	-0.370259	0.145102	-0.143797	0.348437	-0.141042	-0.364189	-1.755531	0.520247	1.009142	-0.136922	-2.070699	-1.351949	-0.029372	39	high_grade
OV-90	-1.617848	-0.523535	0.643578	0.921133	0.75384	0.304277	-0.648242	0.655733	-0.875008	1.322882	-0.687409	0.756798	0.337739	-0.25128	0.043166	-0.229459	0.435774	0.521422	1.088969	-0.623128	0.035817	-0.247336	0.532617	0.747396	-0.923142	-0.188252	0.417137	0.139917	-0.132884	28	high_grade
Caov-4	0.989709	-0.467946	0.596948	0.77761	-0.298504	0.404151	1.745056	-0.035266	0.456133	0.988135	-0.687409	0.298541	0.329148	0.688999	-0.072921	-0.229459	0.755937	0.136341	0.912119	0.478129	0.771699	0.504309	0.966959	1.417274	0.794696	0.961446	0.561205	1.210298	0.613254	21	high_grade
SNU-8	0.411934	-0.438718	0.758756	0.740428	0.342184	0.53346	1.40012	0.250422	0.028777	1.156721	-0.687409	-0.06206	0.055246	0.76029	0.341108	-0.229459	-0.710918	-0.140684	0.730325	0.044964	1.169986	0.701169	0.521967	0.287954	0.068718	1.176719	0.415058	-0.599154	0.754644	14	high_grade
TYK-nu	1.099461	-2.355113	0.631974	-1.596787	1.152025	0.451038	-0.648242	-1.500192	0.449488	0.184999	-0.687409	0.130836	0.519414	-2.930488	-0.599946	-0.229459	0.167846	0.251907	-1.245869	0.3066	-3.425487	-0.057846	0.70426	-1.937685	0.178778	-3.499591	0.371609	-1.419583	-2.055862	14	high_grade
SK-OV-3	0.30092	1.390568	0.034596	0.219313	-0.472148	-2.424887	-0.648242	0.679782	0.335492	-1.357804	-0.687409	0.356513	0.377915	0.486343	0.279754	-0.229459	-0.058343	0.61571	0.522156	-0.272669	-0.007331	0.355875	0.36772	1.167838	0.475107	0.353861	0.243963	-1.246087	0.238062	15	endometric
TOV-112D	1.291212	-0.492588	-2.470309	0.709604	-1.062688	0.841069	1.65623	-0.082742	0.584442	-0.377096	-0.687409	0.169484	0.765521	-0.89518	-0.113088	-0.229459	0.571987	-0.455257	-1.238804	0.482634	0.130173	2.306574	0.754315	1.195267	-1.965683	0.074162	0.453518	0.233037	0.314799	12	endometric
OVK18	1.069185	0.400851	0.388726	0.088312	-1.500541	0.766427	-0.648242	-1.500192	0.442587	-1.357804	-0.687409	-0.63143	0.726104	-0.683208	0.225021	-0.229459	0.558491	0.539361	-0.639729	0.252213	0.398542	1.125417	0.109103	1.153694	0.289405	0.475378	0.230658	1.168149	0.456842	2	endometric
COV434	-0.144395	-0.422099	-2.470309	0.336444	0.039803	0.810161	-0.648242	-1.500192	0.588787	0.511759	-0.687409	-0.365034	0.267242	-2.930488	0.022862	4.322776	0.104613	0.092951	-0.834476	0.116729	0.037713	1.277009	-1.755531	0.403672	0.39398	0.319815	0.01258	0.306552	0.322962	0	endometric
EFO-21	-0.285685	0.095397	0.463935	0.387303	0.292785	-2.424887	-0.648242	0.486974	0.793775	1.089971	1.215102	-1.131441	0.555799	-0.382075	0.262981	4.167194	0.344048	-0.336771	0.074969	-0.134288	0.970843	0.422197	0.521612	0.262239	0.677072	0.108731	0.403208	0.693731	0.26092	89	clear_cell
JHOC-5	0.830758	-0.82555	0.122269	-0.404291	1.241092	0.348642	-0.648242	0.461889	0.622526	-1.357804	-0.687409	-0.421281	-0.048857	-0.202232	-0.311715	-0.229459	0.303059	0.783844	-1.279778	0.389951	0.683033	-0.51157	0.218975	-0.853795	0.408352	0.628845	0.448528	0.002689	0.56264	48	clear_cell
RMG-I	-2.311683	-0.973406	0.398825	-1.596787	0.453705	-2.424887	-0.648242	-1.500192	0.159131	0.149057	-0.687409	-4.654981	-0.363693	0.231977	-0.399553	-0.229459	0.138854	0.025364	0.848538	0.111258	0.721439	-1.38849	-1.755531	0.121663	0.627904	0.063686	-2.070699	0.144818	0.241654	38	clear_cell
ES-2	0.380396	-0.578551	0.107227	-1.596787	-0.46167	0.539347	-0.648242	1.168437	0.158109	0.355011	-0.687409	-0.123138	0.334454	0.157834	-0.659976	-0.229459	-0.021853	0.411696	-3.43235	-3.920461	0.234486	0.915925	-1.755531	-1.780823	0.279571	0.241248	-2.070699	0.485929	0.238389	37	clear_cell
OVMANA	-3.391542	-1.07083	-0.05587	0.171151	-2.675634	-0.576498	1.134505	-1.500192	-0.415448	-1.357804	-0.687409	-0.337773	0.357195	0.642042	-0.188125	-0.229459	-0.110079	-0.330095	0.470113	0.643221	-0.127291	-3.47499	0.46073	-1.0008	-1.965683	-0.415049	-0.00218	-0.903997	-0.361787	31	clear_cell
OVTOKO	-1.219208	0.973935	0.235943	-1.596787	0.371374	0.495193	-0.648242	-1.500192	0.559649	0.296391	-0.687409	-0.182145	-0.338931	-0.451465	-0.268459	-0.229459	0.315806	-0.212443	-0.877334	-3.920461	-0.424584	-1.27269	0.684557	0.384814	-0.017325	-0.538137	0.333356	-1.380375	-0.265132	16	clear_cell
TOV-21G	0.530517	1.798604	0.122914	-1.596787	-1.841842	0.140275	-0.648242	0.688283	0.397857	-1.357804	-0.687409	1.6181	0.847894	-0.589865	-0.110881	-0.229459	0.357045	0.962825	0.089333	0.407651	0.114526	0.6359	0.471735	0.355242	0.634523	0.232867	0.225253	-0.464867	0.277573	2	clear_cell
SNU-840	1.160014	0.485094	-0.114533	0.984129	0.877337	0.165927	-0.648242	0.52056	-0.289439	-1.357804	-0.687409	-0.340534	0.471152	1.138607	0.789565	-0.229459	0.675459	0.337017	0.703008	0.454314	0.214572	-0.046266	0.180991	0.083948	0.278058	0.265342	0.737705	0.628058	0.242307	38	

Supplementary Table 5 |Patient information

AGO Parafin blocks

Patient Demographics

Characteristic	Value
Total patients	27
Median age at diagnosis	59 years (range: 21-76)

BRCA Status

BRCA Status	n (%)
BRCA mutated	12 (44.4%)
Wild type	15 (55.6%)

Histology Distribution

Histology Type	n (%)
High grade serous	21 (77.8%)
High grade endometrial	2 (7.4%)
Low grade endometrial	1 (3.7%)
Clear cell	1 (3.7%)
Mucinous	1 (3.7%)
Other/unspecified	1 (3.7%)

Clinical Characteristics

Characteristic	Category	n (%)
FIGO Stage	IA	2 (7.4%)
	IIC	1 (3.7%)
	IIIB	3 (11.1%)
	IIIC	17 (63.0%)
	IV	4 (14.8%)
Tumor Grade	G1	2 (7.4%)
	G2	2 (7.4%)
	G3	23 (85.2%)
Patient Group	Primary diagnosis	18 (66.7%)
	Platinum-sensitive recurrence	9 (33.3%)

Plasma Mannheim Cohort 2

Patient Demographics

Characteristic	Value
Total patients	16
Median age at diagnosis	67.5 years (range: 38-80)

Histology Distribution

Histology Type	n (%)
High-grade serous carcinoma (M8461/3)	13 (81.3%)
Mucinous adenocarcinoma (M8480/3)	1 (6.3%)
Adenocarcinoma NOS (M8140/3)	1 (6.3%)
Carcinoma NOS (M8010/3)	1 (6.3%)

Clinical Characteristics

Characteristic	Category	n (%)
Tumor stage	T1	2 (12.5%)
	T3	14 (87.5%)
Lymph node involvement	Node negative	8 (50.0%)
	Node positive	4 (25.0%)
	Not assessed/reported	4 (25.0%)
Distant metastasis	M0	9 (56.3%)
	M1	7 (43.8%)
Resection status	R0	6 (37.5%)
	R1	7 (43.8%)
	R2	1 (6.3%)
	Not reported	2 (12.5%)

Plasma HIPO H059 Cohort 1

Patient Demographics

Characteristic	Value
Total patients	24
Median age at surgery	64.1 years (range: 21.6-76.9)

BRCA Status

BRCA Status	n (%)
No mutation	17 (70.8%)
BRCA1 germline INDEL	6 (25.0%)
BRCA1 somatic INDEL	1 (4.2%)
Any BRCA1 alteration	7 (29.2%)

Tumor Characteristics

Characteristic	Value
Mean HRD score	47.5
Median HRD score	51

Histology Distribution

Histology Type	n (%)
High grade serous	24 (100%)

Sample Type Distribution

Sample Type	n (%)
Primary tumor samples	23 (95.8%)
Metastasis samples	1 (4.2%)

Supplementary Table 6 |used R packages

Package	Version	Source	Description
tidyverse	2.0.0	https://cran.r-project.org/package=tidyverse	Data wrangling and visualization tools
tidymodels	1.1.1	https://cran.r-project.org/package=tidymodels	Machine learning framework for modeling and evaluation
ranger	0.12.1	https://cran.r-project.org/package=ranger	Fast random forest implementation
vip	0.3.1	https://cran.r-project.org/package=vip	Variable importance plots
finetune	1.1.0	https://cran.r-project.org/package=finetune	Hyperparameter tuning with tidymodels
tune	1.0.0	https://cran.r-project.org/package=tune	Hyperparameter tuning and model optimization
shapviz	0.3.1	https://cran.r-project.org/package=shapviz	SHAP (Shapley additive explanations) visualizations
ComplexHeatmap	2.10.0	https://bioconductor.org/packages/release/bioc/html/ComplexHeatmap .html	Visualization of complex heatmaps
circlize	0.4.15	https://cran.r-project.org/package=circlize	Circular visualization tools
caret	6.0-91	https://cran.r-project.org/package=caret	Classification and regression training
randomForest	4.7-1.1	https://cran.r-project.org/package=randomForest	Random forest for classification and regression
rpart	4.1-15	https://cran.r-project.org/package=rpart	Recursive partitioning for regression and classification
rpart.plot	3.0.9	https://cran.r-project.org/package=rpart.plot	Plotting decision trees
multiROC	1.1.0	https://cran.r-project.org/package=multiROC	Multi-class ROC analysis
C50	0.1.0	https://cran.r-project.org/package=C50	C5.0 decision tree implementation
kernlab	0.9-31	https://cran.r-project.org/package=kernlab	Kernel-based machine learning algorithms
mlbench	2.1-3	https://cran.r-project.org/package=mlbench	Machine learning benchmark datasets
caretEnsemble	2.0.0	https://cran.r-project.org/package=caretEnsemble	Ensemble models with caret
ggplot2	3.3.6	https://cran.r-project.org/package=ggplot2	Data visualization with grammar of graphics
doSNOW	1.0.20	https://cran.r-project.org/package=doSNOW	Parallel computing with snow
RColorBrewer	1.1-2	https://cran.r-project.org/package=RColorBrewer	Color palettes for data visualization
survival	3.2-13	https://cran.r-project.org/package=survival	Survival analysis
survminer	0.4.9	https://cran.r-project.org/package=survminer	Visualizing survival curves
glmnet	4.1-5	https://cran.r-project.org/package=glmnet	Elastic net and lasso regularization for regression
TCGAbiolinks	2.22.2	https://bioconductor.org/packages/release/bioc/html/TCGAbiolinks.html	Accessing and analyzing TCGA data
DT	0.25	https://cran.r-project.org/package=DT	Interactive data tables
psych	2.2.9	https://cran.r-project.org/package=psych	Psychological research and data analysis
reshape2	1.4.4	https://cran.r-project.org/package=reshape2	Reshaping data
ggpmisc	0.3.9	https://cran.r-project.org/package=ggpmisc	Adding statistical annotations to ggplot2
ggpubr	0.4.0	https://cran.r-project.org/package=ggpubr	Publication-ready ggplot2 graphs
corrplot	0.92	https://cran.r-project.org/package=corrplot	Visualizing correlation matrices
corrr	0.4.3	https://cran.r-project.org/package=corrr	Correlation analysis and visualization
VennDiagram	1.6.20	https://cran.r-project.org/package=VennDiagram	Venn diagram generation
stringr	1.4.0	https://cran.r-project.org/package=stringr	String manipulation functions
keras	2.10.0	https://cran.r-project.org/package=keras	Interface to 'Keras' deep learning library
lime	0.5.0	https://cran.r-project.org/package=lime	Local interpretable model-agnostic explanations
tidyquant	1.0.5	https://cran.r-project.org/package=tidyquant	Quantitative finance tools
rsample	1.1.0	https://cran.r-project.org/package=rsample	Resampling functions for model validation
recipes	0.2.0	https://cran.r-project.org/package=recipes	Preprocessing tools for machine learning models
yardstick	0.0.8	https://cran.r-project.org/package=yardstick	Performance metrics for machine learning models
pROC	1.18.0	https://cran.r-project.org/package=pROC	ROC curve analysis
ggbiplot	0.55	https://cran.r-project.org/package=ggbiplot	PCA biplot visualization
Rtsne	0.15	https://cran.r-project.org/package=Rtsne	t-SNE algorithm for dimensionality reduction
kohonen	3.0.10	https://cran.r-project.org/package=kohonen	Self-organizing maps
hrbrthemes	0.8	https://cran.r-project.org/package=hrbrthemes	Beautiful ggplot2 themes
GGally	2.1.2	https://cran.r-project.org/package=GGally	Extension of ggplot2 for creating scatterplot matrices
viridis	0.6.2	https://cran.r-project.org/package=viridis	Color scales for ggplot2
ReactomePA	1.34.0	https://bioconductor.org/packages/ReactomePA	For Reactome pathway analysis.
DOSE	3.20.0	https://bioconductor.org/packages/DOSE	For disease ontology-based analysis.
graphite	1.32.0	https://bioconductor.org/packages/graphite	For pathway-based analysis.
GOSemSim	2.18.0	https://bioconductor.org/packages/GOSemSim	For Gene Ontology-based semantic similarity.
clusterProfiler	4.2.2.	https://bioconductor.org/packages/clusterProfiler	For functional enrichment analysis.

Supplementary Table 7 | Antibody list

Target Protein	Host Species	Catalog Number	Supplier	Dilution (WB/IF/IHC)
MCM2	Rabbit	PA5-32484	Invitrogen	1:1000
MCM7	Rabbit	3735S	CellSignaling	1:1000
53BP1	Rabbit	88439S	CellSignaling	1:1000
GAPDH	Mouse	14-9523-80	Invitrogen	1:1000
pyH2AX	-	AB22551	Abcam	1:1000
RAD51	Rabbit	ab63801	Abcam	1:1000
p-p65	-	3033	CellSignaling	1:1000
p-stat1	-	9167	CellSignaling	1:1000
BrdU/CIdU	Rat	ab6326	Abcam	1:200
BrdU/IdU	Mouse	347580	Becton Dickinson	1:100
pChk1	Rabbit	2348T	CellSignaling	1:1000 (1:56)
pChk2	Rabbit	2197S	CellSignaling	1:1000 (1:62)

Antibody Name	Catalog Number	Supplier	Dilution (WB/IF/IHC)
Anti-RAT Cy3	ab98416	Abcam	1:300
Anti-Mouse Alexa 488	A11001	Invitrogen	1:300
Anti-Rabbit Alexa 488	A11034	Invitrogen	1:250
Anti-Mouse Alexa 594	A11005	Invitrogen	1:250
Anti-Rabbit Alexa 594	A11037	Invitrogen	1:250
Goat anti-Rabbit POX	31466	Invitrogen	1:10000
Goat anti-Mouse POX	31431	Invitrogen	1:10000

Supplementary Table 8 |PCR primer list

Gene / lncRNA	Forward Primer (5’→3’)	Reverse Primer (5’→3’)
ENSG00000233029.3	GCCTAGAACGCGTATGGAGA	CACGCCTCTGTGTTCAGGAT
ENSG00000234264.1	ATA GAG GGA GCG GAT AAG GAA G	TAG AGG AGT GCC AGG ATT GTA G
ENSG00000255471.1	TGCCACAAGGGCCACAAATA	CCCAGTGAAATCCCACCGAT
ENSG00000255651.2	CGTTGCAGACAAAGAAGCTCA	GTCCTGGCTTCACTGGTAACT
ENSG00000257671.1	GGG TTT CCT CTG TGG ATG TT	GCC TTC ATG GGC ATA TTG TTG
ENSG00000260369.2	GAG CGA TCT CGG AAA GTA CAG	CGG TGA GGA CCT TGA CTT AAA
ENSG00000261183.1	TGA GCC AGC TGT GAT GTA AAG	CCT TTA GAG TCT CTC ATC CTC CA
ENSG00000261824.2	GTT GTC TGG GCA ACC TTA GA	TCA GGT TTC AGT CAG TGA GAT G
ENSG00000264885.1	GTC ACG GGT CCC TAA CAA TAA T	GCC TCG GAT AGT TCT CAT CAT AAA
ENSG00000266999.1	CAC TTT GAG TTG TTC CAC CTT TC	GAG AGG CAT GAG AAC TTC AAT CA
ENSG00000267575.2	GGCTTTTACCTGGTCTGCGA	GCGGCTGATCTCACCTTGTA
ENSG00000272172.1	CCC TAA GAA GAC AGC AAC AGA G	GGT TCC AGG CAC GAG ATG
ENSG00000272635.1	GCACAAGTCATGAAGCCGAC	GAGAAGATAGCTTGGCCGGG
ATR	TCCCTTGAATACAGTGGCCTA	TCCTTGAAAGTACGGCAGTTC
CDK12	GCACAGTTATCAAACCTTTGGTG	CGTTCTCCCTTCGGTCTAGTT
CDKN1A (P21)	TGTCCGTCAGAACCCATGC	AAAGTCGAAGTTCCATCGCTC
CDT1	GAC ATG ATG CGT AGG CGT TTT	GAG CTG GTA ATC TGA CCT CCT
FEN1	CACCTGATGGGCATGTTCTAC	CTCGCCTGACTTGAGCTGT
GAPDH	AAT GCA TCC TGC ACC ACC AA	GTC ATT GAG AGC AAT GCC AGC
MCM7	CCTACCAGCCGATCCAGTCT	CCTCCTGAGCGGTTGGTTT
PARP11	TTTGTGGAAGCAATCTGCATTCA	GCAGCATCTCTAGCAAAATAGGT
PCNA	CCTGCTGGGATATTAGCTCCA	CAGCGGTAGGTGTCGAAGC
POLD1	CAG TGC CAA GGT GGT GTA TGG	CTT GCT GAT AAG CAG GTA TGG G
POLD2	CCA TCA GCC AAC AAT GCC AC	CTA GCC GGA AGG GTT GTG A
POLD3	GAG TTC GTC ACG GAC CAA AAC	GCC AGA CAC CAA GTA GGT AAC
POLD4_1	ATC ACT GAT TCC TAC CCG GTT	AGA GAT GCC AGA GAC TGC ACT
POLG	CATTCGTGAGAACTTCCAGGAC	GTGGGGACACCTCTCCAAG
POLH	AAGGGTGGTGGAATAATTGCAG	CTCACGAACTTGTGCCAGTAG
POLK	TGAGGGACAATCCAGAATTGAAG	CTGCACGAACACCAAATCTCC
POLQ	ACT TTT GCT GAC CAA GAT TTG CT	ACT CAT GCC AAC GAT TTG CAC
RAD17	AAAGCCCGAGGATATGCTCAT	CCAGGCAATTTTCCCGATACTT
RAD50	TTTGGTTGGACCCAATGGGG	CAGGAGGGAAATCTCCAGTACAA
TOP1MT	ACGAAGACGGGGTGAAGTG	CCGGAAAACCTCCTTTGTTGTG
TOPBP1	TTCAGCAACTCACAGTTAAGCA	GGCACACTCATACTTCTGACC
TRESLIN	CCGTGGAGCCAAAAGGATCT	GTGGAGCCGGAAACAAACAC
XRCC5	GCACTGACAATCCCCTTTCTG	TCAATGTCCTCCAGCAAATCAAA
53BP1	ATG GAC CCT ACT GGA AGT CAG	TTT CTT TGT GCG TCT GGA GAT T
APEX2	CTG GAA CAT CAA TGG GAT TCG G	CCA GCT CGT CCA AAA TGC G
ATM	ATC TGC TGC CGT CAA CTA GAA	GAT CTC GAA TCA GGC GCT TAA A
BRCA1	GAA ACC GTG CCA AAA GAC TTC	CCA AGG TTA GAG AGT TGG ACA C
BRCA2	CTG CTC GCG TTG TAC TAA CAT	TCC AAT GCA GTC ACT TAC ACA AT
BRD1	CTG CTC GCG TTG TAC TAA CAT	TCC AAT GCA GTC ACT TAC ACA AT
CDK12	GCACAGTTATCAAACCTTTGGTG	CGTTCTCCCTTCGGTCTAGTT
CHEK1	ATA TGA AGC GTG CCG TAG ACT	TGC CTA TGT CTG GCT CTA TTC TG
CHEK2	TGA GAA CCT TAT GTG GAA CCC C	ACA GCA CGG TTA TAC CCA GC
EZH2	AAT CAG AGT ACA TGC GAC TGA GA	GCT GTA TCC TTC GCT GTT TCC
FANCC	TCA AGG TCT TGG GTA TGC ACC	GCC ATT CGC CTT TGA GTG TTA AA
LIG1	GAA GGA GGC ATC CAA TAG CAG	ACT CTC GGA CAC CAC TCC ATT
MU581	AGC CCG AGT GAT ACT GCT G	TCC TCC TTG GTT AAG AAG TGG T
PALB2	AGG ATC TCT CAC CGC AGC TAA	TCA GGC CCA ACA TCA AGT GTG
PARG	AGC TTT TGG AAA GTG AAC CTC A	ATC TTC CGT AGT CTG CTT TGC
PARP1	TGGAAAAGTCCCACACTGGTA	AAGCTCAGAGAACCCATCCAC
RAD51	CAA CCC ATT TCA CGG TTA GAG C	TTC TTT GGC GCA TAG GCA ACA
RAD51C	TTT GGT GAG TTT CCC GCT GTC	AAC TTC TTT GCT AAG CTC GGA G
RAD51D	CCA GCA CTC GGA TTC TCC TG	TTG GCT GTC GGG AAG ATT TGG
XRCC1	TCA AGG CAG ACA CTT ACC GAA	TCC AAC TGT AGG ACC ACA GAG
XRCC2	ACTGCAGTAGTAGCACCCAC	TCCATTGACGCGGTCTATCC
