# Supplementary material for: Long non-coding RNAs as a biomarker for homologous recombination deficiency and parp inhibitor sensitivity in high-grade serous ovarian cancers
Source: Commun Biol. 2025 Oct 1;8:1410. doi: 10.1038/s42003-025-08836-9 (PMC12488886; doi:10.1038/s42003-025-08836-9)
Supplement: Supplementary file 2 — Description of Additional Supplementary Files [file 42003_2025_8836_MOESM2_ESM.pdf]

Description of Additional Supplementary Files

File name: Supplementary Data File 1

Description: TCGA ovarian lncRNA data with associated scores.

File name: Supplementary Data File 2

Description: Values to generate the main figures
