# Supplementary material for: Long non-coding RNAs as a biomarker for homologous recombination deficiency and parp inhibitor sensitivity in high-grade serous ovarian cancers
Source: Commun Biol. 2025 Oct 1;8:1410. doi: 10.1038/s42003-025-08836-9 (PMC12488886; doi:10.1038/s42003-025-08836-9)
Supplement: Supplementary file 5 — Reporting Summary [file 42003_2025_8836_MOESM5_ESM.pdf]

nature portfolio

Corresponding author(s): Kai Doberstein

Last updated by author(s): Aug 6, 2025

Reporting Summary

Nature Portfolio wishes to improve the reproducibility of the work that we publish. This form provides structure for consistency and transparency in reporting. For further information on Nature Portfolio policies, see our Editorial Policies and the Editorial Policy Checklist.

Statistics

For all statistical analyses, confirm that the following items are present in the figure legend, table legend, main text, or Methods section.

n/a	Confirmed	
☐	☒	The exact sample size (*n*) for each experimental group/condition, given as a discrete number and unit of measurement
☐	☒	A statement on whether measurements were taken from distinct samples or whether the same sample was measured repeatedly
☐	☒	The statistical test(s) used AND whether they are one- or two-sided<br>*Only common tests should be described solely by name; describe more complex techniques in the Methods section.*
☐	☒	A description of all covariates tested
☐	☒	A description of any assumptions or corrections, such as tests of normality and adjustment for multiple comparisons
☐	☒	A full description of the statistical parameters including central tendency (e.g. means) or other basic estimates (e.g. regression coefficient) AND variation (e.g. standard deviation) or associated estimates of uncertainty (e.g. confidence intervals)
☐	☒	For null hypothesis testing, the test statistic (e.g. *F*, *t*, *r*) with confidence intervals, effect sizes, degrees of freedom and *P* value noted<br>*Give P values as exact values whenever suitable.*
☒	☐	For Bayesian analysis, information on the choice of priors and Markov chain Monte Carlo settings
☒	☐	For hierarchical and complex designs, identification of the appropriate level for tests and full reporting of outcomes
☐	☒	Estimates of effect sizes (e.g. Cohen's *d*, Pearson's *r*), indicating how they were calculated

Our web collection on statistics for biologists contains articles on many of the points above.

Software and code

Policy information about availability of computer code

Data collection	Data from the TCGA ovarian cancer cohort (phs000178.v11.p8) were extracted and downloaded via the UCSC XENA portal (http://xena.ucsc.edu/) and cBioPortal (https://www.cbioportal.org). Cell line expression and drug response data were obtained from DepMap (https://depmap.org/, release 23Q4). lncRNA expression data from both TCGA and CCLE were retrieved from the TANRIC database (https://ibl.mdanderson.org/tanric), hosted by MD Anderson. Genomic instability scores were sourced from Knijnenburg et al.; BRCA aberration data from Kraya et al.; and HRD-scores for CCLE from Takamatsu et al. Data analyses were performed using the R programming language and GraphPad Prism. Machine learning models were implemented using R packages including tidymodels, ranger, kernlab, kknn, xgboost, glmnet, lme4, baguette, ipred, finetune, and partykit. Feature selection was conducted using caret, randomForest, reprtree, glmnet, psych, and corrr; UMAP was used for dimensionality reduction. Pathway analyses were performed using ReactomePA, DOSE, graphite, GOSemSim, and clusterProfiler. All custom code and documentation are openly available at: https://github.com/kaidob/lncRNA_HRD_PAPER_2025.git.
Data analysis	All data analyses were performed using the R programming language (v4.2.3) and GraphPad Prism. Image-based quantification of nuclear foci and fluorescence intensity was conducted using CellProfiler (Broad Institute), an open-source software for high-throughput image analysis. The complete CellProfiler pipeline, including all input settings and modules, is publicly available at: https://github.com/kaidob/lncRNA_HRD_PAPER_2025.git.A comprehensive suite of R packages was used for statistical analysis, visualization, machine learning, and pathway enrichment, including but not limited to:Machine learning and modeling: tidymodels, ranger, vip, finetune, tune, shapviz, caret, randomForest, rpart, C50, kernlab, mlbench, caretEnsemble, glmnet, keras, lime, rsample, recipes, yardstick, pROCVisualization and data handling: ggplot2, ComplexHeatmap, circlize, multiROC, doSNOW, ggpubr, ggpmisc, RColorBrewer, reshape2, DT, hrbrthemes, viridis, GGally, corrplot, corrr, VennDiagram, stringr, tidyverse, tidyquant, ggbiplot, Rtsne, kohonenSurvival and statistical testing: survival, survminer, psychPathway and enrichment analysis: ReactomePA, DOSE, graphite, GOSemSim, clusterProfilerData acquisition and management: TCGAbiolinksAll package versions used are listed in Supplementary Table S6. Full analysis scripts and documentation are available on GitHub:

https://github.com/kaidob/lncRNA_HRD_PAPER_2025.git.

For manuscripts utilizing custom algorithms or software that are central to the research but not yet described in published literature, software must be made available to editors and reviewers. We strongly encourage code deposition in a community repository (e.g. GitHub). See the Nature Portfolio guidelines for submitting code & software for further information.

Data

Policy information about availability of data

All manuscripts must include a data availability statement. This statement should provide the following information, where applicable:

- Accession codes, unique identifiers, or web links for publicly available datasets
- A description of any restrictions on data availability
- For clinical datasets or third party data, please ensure that the statement adheres to our policy

All data used in this study are either included in the Supplementary Data files or are publicly available through the TCGA (dbGaP Study Accession: phs000178.v11.p8) and CCLE data portals. CCLE expression and drug response data are summarized in Supplementary Table S4, and TCGA ovarian cancer data with associated scores are provided in Supplementary Table S9. All custom code and analysis pipelines are available at: https://github.com/kaidob/lncRNA_HRD_PAPER_2025.git.

Research involving human participants, their data, or biological material

Policy information about studies with human participants or human data. See also policy information about sex, gender (identity/presentation), and sexual orientation and race, ethnicity and racism.

Reporting on sex and gender	This study about ovarian cancer, can contain only samples from female patients.
Reporting on race, ethnicity, or other socially relevant groupings	No information were collected.
Population characteristics	Patient demographics are described in Table S5
Recruitment	We used samples of patients that were recruded for other studies.
Ethics oversight	Ethik-Kommission II<br>Medizinische Fakultät Mannheim<br>der Ruprecht-Karls-Universität Heidelberg<br>Zentrum Medizinischer Forschung<br>2011-380N-MA

Note that full information on the approval of the study protocol must also be provided in the manuscript.

Field-specific reporting

Please select the one below that is the best fit for your research. If you are not sure, read the appropriate sections before making your selection.

☒ Life sciences ☐ Behavioural & social sciences ☐ Ecological, evolutionary & environmental sciences

For a reference copy of the document with all sections, see nature.com/documents/nr-reporting-summary-flat.pdf

Life sciences study design

All studies must disclose on these points even when the disclosure is negative.

Sample size	The sample size used in this study was determined based on practical constraints, including the limited availability of high-quality, clinically annotated patient samples and the difficulty in obtaining sufficient material for both tissue- and plasma-based lncRNA analyses. Analytically, we prioritized consistency and reproducibility across biological replicates rather than large-scale cohort expansion. Despite these limitations, we observed statistically significant and biologically consistent results across independent experiments. Statistical analyses, including unpaired two-tailed t-tests, Pearson correlation, and one-way ANOVA, consistently demonstrated robust differences and associations, supporting the reliability of our findings at the current sample size.
Data exclusions	Patient samples were excluded from the analysis if, following RNA isolation, the RNA quality or quantity was insufficient for reliable downstream analysis. Quality control criteria included RNA integrity (as assessed by RIN scores or electropherogram profiles) and minimum concentration thresholds required for s qPCR assays. Only samples meeting these predefined quality standards were included in the final dataset.
Replication	All experiments were performed with a minimum of three independent biological replicates to ensure reproducibility and reliability of the results. Technical replicates were included where applicable, and consistent findings across replicates support the robustness of the observed effects.

Randomization

All available samples that met quality control criteria were included and measured. No additional randomization was applied, as the study design involved comprehensive analysis of the full available cohort.

Blinding

Blinding was not relevant to this study, as all analyses were based on objective molecular measurements and predefined computational pipelines.

Reporting for specific materials, systems and methods

We require information from authors about some types of materials, experimental systems and methods used in many studies. Here, indicate whether each material, system or method listed is relevant to your study. If you are not sure if a list item applies to your research, read the appropriate section before selecting a response.

Materials & experimental systems

n/a	Involved in the study
☐	☒ Antibodies
☐	☒ Eukaryotic cell lines
☐	☐ Palaeontology and archaeology
☐	☐ Animals and other organisms
☐	☒ Clinical data
☐	☐ Dual use research of concern
☐	☐ Plants

Methods

n/a	Involved in the study
☐	☐ ChIP-seq
☐	☒ Flow cytometry
☐	☐ MRI-based neuroimaging

Antibodies

Antibodies used

Antibody list
Target Protein Host Species Catalog Number Supplier Dilution (WB/IF/IHC)
MCM2 Rabbit PA5-32484 Invitrogen 1:1000
MCM7 Rabbit 3735S CellSignaling 1:1000
53BP1 Rabbit 88439S CellSignaling 1:1000
GAPDH Mouse 14-9523-80 Invitrogen 1:1000
pyH2AX - AB22551 Abcam 1:1000
RAD51 Rabbit ab63801 Abcam 1:1000
p-p65 - 3033 CellSignaling 1:1000
p-stat1 - 9167 CellSignaling 1:1000
BrdU/CldU Rat ab6326 Abcam 1:100
BrdU/IdU Mouse 347580 Becton Dickinson 1:250
pChk1 Rabbit 2348T CellSignaling 1:1000 (1:56)
pChk2 Rabbit 2197S CellSignaling 1:1000 (1:62)

Antibody Name Catalog Number Supplier Dilution (WB/IF/IHC)
Anti-RAT Cy3 ab98416 Abcam 1:300
Anti-Mouse Alexa 488 A11001 Invitrogen 1:300
Anti-Rabbit Alexa 488 A11034 Invitrogen 1:250
Anti-Mouse Alexa 594 A11005 Invitrogen 1:250
Anti-Rabbit Alexa 594 A11037 Invitrogen 1:250
Goat anti-Rabbit POX 31466 Invitrogen 1:10000
Goat anti-Mouse POX 31431 Invitrogen 1:10000

Validation

All primary antibodies used in this study were validated for the indicated species and applications either by the manufacturer or through prior peer-reviewed publications. Validation information, including specificity and application suitability, was confirmed via the manufacturer's datasheets and, where applicable, supported by references listed in the manuscript. Additional validation details (e.g., observed band sizes, expected subcellular localization) are were performed during experiments.

Eukaryotic cell lines

Policy information about cell lines and Sex and Gender in Research

Cell line source(s)

The cell lines FT282 FT240, FT246, KURAMOCHI, OVKATE, COV318 and OVSAHO cells were a gift from Ronny Drapkin (UPenn, Philadelphia, USA). The ovarian cancer cell lines (OVCAR8, OVCAR3, CAOV4 and CAOV3) were obtained from American Type Culture Collection (ATCC, Manassas, VA).

Authentication

All cancer cell lines were profiled using Eurofins CLA service.

Mycoplasma contamination	No Mycoplasma contamination has been detected.
Commonly misidentified lines (See ICLAC register)	*Name any commonly misidentified cell lines used in the study and provide a rationale for their use.*

Palaeontology and Archaeology

Specimen provenance	*Provide provenance information for specimens and describe permits that were obtained for the work (including the name of the issuing authority, the date of issue, and any identifying information). Permits should encompass collection and, where applicable, export.*
Specimen deposition	*Indicate where the specimens have been deposited to permit free access by other researchers.*
Dating methods	*If new dates are provided, describe how they were obtained (e.g. collection, storage, sample pretreatment and measurement), where they were obtained (i.e. lab name), the calibration program and the protocol for quality assurance OR state that no new dates are provided.*

☐ Tick this box to confirm that the raw and calibrated dates are available in the paper or in Supplementary Information.

Ethics oversight	*Identify the organization(s) that approved or provided guidance on the study protocol, OR state that no ethical approval or guidance was required and explain why not.*

Note that full information on the approval of the study protocol must also be provided in the manuscript.

Animals and other research organisms

Policy information about studies involving animals; ARRIVE guidelines recommended for reporting animal research, and Sex and Gender in Research

Laboratory animals	*For laboratory animals, report species, strain and age OR state that the study did not involve laboratory animals.*
Wild animals	*Provide details on animals observed in or captured in the field; report species and age where possible. Describe how animals were caught and transported and what happened to captive animals after the study (if killed, explain why and describe method; if released, say where and when) OR state that the study did not involve wild animals.*
Reporting on sex	*Indicate if findings apply to only one sex; describe whether sex was considered in study design, methods used for assigning sex. Provide data disaggregated for sex where this information has been collected in the source data as appropriate; provide overall numbers in this Reporting Summary. Please state if this information has not been collected. Report sex-based analyses where performed, justify reasons for lack of sex-based analysis.*
Field-collected samples	*For laboratory work with field-collected samples, describe all relevant parameters such as housing, maintenance, temperature, photoperiod and end-of-experiment protocol OR state that the study did not involve samples collected from the field.*
Ethics oversight	*Identify the organization(s) that approved or provided guidance on the study protocol, OR state that no ethical approval or guidance was required and explain why not.*

Note that full information on the approval of the study protocol must also be provided in the manuscript.

Clinical data

Policy information about clinical studies

All manuscripts should comply with the ICMJE guidelines for publication of clinical research and a completed CONSORT checklist must be included with all submissions.

Clinical trial registration	*Provide the trial registration number from ClinicalTrials.gov or an equivalent agency.*
Study protocol	*Note where the full trial protocol can be accessed OR if not available, explain why.*
Data collection	*Describe the settings and locales of data collection, noting the time periods of recruitment and data collection.*
Outcomes	*Describe how you pre-defined primary and secondary outcome measures and how you assessed these measures.*

Dual use research of concern

Policy information about dual use research of concern

Hazards

Could the accidental, deliberate or reckless misuse of agents or technologies generated in the work, or the application of information presented in the manuscript, pose a threat to:

No	Yes	
☒	☐	Public health
☒	☐	National security
☒	☐	Crops and/or livestock
☒	☐	Ecosystems
☒	☐	Any other significant area

Experiments of concern

Does the work involve any of these experiments of concern:

No	Yes	
☒	☐	Demonstrate how to render a vaccine ineffective
☒	☐	Confer resistance to therapeutically useful antibiotics or antiviral agents
☒	☐	Enhance the virulence of a pathogen or render a nonpathogen virulent
☒	☐	Increase transmissibility of a pathogen
☒	☐	Alter the host range of a pathogen
☒	☐	Enable evasion of diagnostic/detection modalities
☒	☐	Enable the weaponization of a biological agent or toxin
☒	☐	Any other potentially harmful combination of experiments and agents

Plants

Seed stocks	*Report on the source of all seed stocks or other plant material used. If applicable, state the seed stock centre and catalogue number. If plant specimens were collected from the field, describe the collection location, date and sampling procedures.*
Novel plant genotypes	*Describe the methods by which all novel plant genotypes were produced. This includes those generated by transgenic approaches, gene editing, chemical/radiation-based mutagenesis and hybridization. For transgenic lines, describe the transformation method, the number of independent lines analyzed and the generation upon which experiments were performed. For gene-edited lines, describe the editor used, the endogenous sequence targeted for editing, the targeting guide RNA sequence (if applicable) and how the editor was applied.*
Authentication	*Describe any authentication procedures for each seed stock used or novel genotype generated. Describe any experiments used to assess the effect of a mutation and, where applicable, how potential secondary effects (e.g. second site T-DNA insertions, mosiacism, off-target gene editing) were examined.*

ChIP-seq

Data deposition

☐ Confirm that both raw and final processed data have been deposited in a public database such as GEO.

☐ Confirm that you have deposited or provided access to graph files (e.g. BED files) for the called peaks.

Data access links<br>*May remain private before publication.*	*For "Initial submission" or "Revised version" documents, provide reviewer access links. For your "Final submission" document, provide a link to the deposited data.*
Files in database submission	*Provide a list of all files available in the database submission.*
Genome browser session<br>(e.g. UCSC)	*Provide a link to an anonymized genome browser session for "Initial submission" and "Revised version" documents only, to enable peer review. Write "no longer applicable" for "Final submission" documents.*

Methodology

Replicates	*Describe the experimental replicates, specifying number, type and replicate agreement.*
Sequencing depth	*Describe the sequencing depth for each experiment, providing the total number of reads, uniquely mapped reads, length of reads and whether they were paired- or single-end.*
Antibodies	*Describe the antibodies used for the ChIP-seq experiments; as applicable, provide supplier name, catalog number, clone name, and lot number.*

Peak calling parameters	*Specify the command line program and parameters used for read mapping and peak calling, including the ChIP, control and index files used.*
Data quality	*Describe the methods used to ensure data quality in full detail, including how many peaks are at FDR 5% and above 5-fold enrichment.*
Software	*Describe the software used to collect and analyze the ChIP-seq data. For custom code that has been deposited into a community repository, provide accession details.*

Flow Cytometry

Plots

Confirm that:

☒ The axis labels state the marker and fluorochrome used (e.g. CD4-FITC).

☒ The axis scales are clearly visible. Include numbers along axes only for bottom left plot of group (a 'group' is an analysis of identical markers).

☒ All plots are contour plots with outliers or pseudocolor plots.

☐ A numerical value for number of cells or percentage (with statistics) is provided.

Methodology

Sample preparation	Cells were incubated with 10μM EdU for 20–30 minutes, washed with PBS, and fixed in 70% ethanol at −20°C for 1 hour. After washing, EdU incorporation was detected using a click chemistry reaction (10mM 6-Carboxyfluorescein-TEG-azide, 10mM sodium ascorbate, 2mM $CuSO_4$ in PBS) for 30 minutes in the dark. Cells were then washed and stained with 1μg/mL DAPI. Fluorescence was measured on a BD FACS Canto II, acquiring 10,000 single cells per sample, and analyzed using FlowJo. S phase cells were identified via DAPI vs. EdU dot plots, and EdU intensity was exported for individual cells. Data were plotted in GraphPad Prism as scatter dot plots. Mean fluorescence intensity was normalized to the untreated control for each biological replicate (n=3) and presented as relative expression.
Instrument	BD FACS Canto II,
Software	FlowJo
Cell population abundance	n/a
Gating strategy	Initial gating was performed on forward scatter (FSC) and side scatter (SSC) to exclude debris and select the main cell population. Next, doublets were excluded by plotting DAPI width (355–450/50 linear mode) against DAPI area, and gating on the singlet population to ensure only single nuclei were analyzed.

☐ Tick this box to confirm that a figure exemplifying the gating strategy is provided in the Supplementary Information.

Magnetic resonance imaging

Experimental design

Design type	*Indicate task or resting state; event-related or block design.*
Design specifications	*Specify the number of blocks, trials or experimental units per session and/or subject, and specify the length of each trial or block (if trials are blocked) and interval between trials.*
Behavioral performance measures	*State number and/or type of variables recorded (e.g. correct button press, response time) and what statistics were used to establish that the subjects were performing the task as expected (e.g. mean, range, and/or standard deviation across subjects).*

Acquisition

Imaging type(s)	*Specify: functional, structural, diffusion, perfusion.*
Field strength	*Specify in Tesla*
Sequence & imaging parameters	*Specify the pulse sequence type (gradient echo, spin echo, etc.), imaging type (EPI, spiral, etc.), field of view, matrix size, slice thickness, orientation and TE/TR/flip angle.*
Area of acquisition	*State whether a whole brain scan was used OR define the area of acquisition, describing how the region was determined.*

Diffusion MRI ☐ Used ☐ Not used

Preprocessing

Preprocessing software — *Provide detail on software version and revision number and on specific parameters (model/functions, brain extraction, segmentation, smoothing kernel size, etc.).*

Normalization — *If data were normalized/standardized, describe the approach(es): specify linear or non-linear and define image types used for transformation OR indicate that data were not normalized and explain rationale for lack of normalization.*

Normalization template — *Describe the template used for normalization/transformation, specifying subject space or group standardized space (e.g. original Talairach, MNI305, ICBM152) OR indicate that the data were not normalized.*

Noise and artifact removal — *Describe your procedure(s) for artifact and structured noise removal, specifying motion parameters, tissue signals and physiological signals (heart rate, respiration).*

Volume censoring — *Define your software and/or method and criteria for volume censoring, and state the extent of such censoring.*

Statistical modeling & inference

Model type and settings — *Specify type (mass univariate, multivariate, RSA, predictive, etc.) and describe essential details of the model at the first and second levels (e.g. fixed, random or mixed effects; drift or auto-correlation).*

Effect(s) tested — *Define precise effect in terms of the task or stimulus conditions instead of psychological concepts and indicate whether ANOVA or factorial designs were used.*

Specify type of analysis: ☐ Whole brain ☐ ROI-based ☐ Both

Statistic type for inference — *Specify voxel-wise or cluster-wise and report all relevant parameters for cluster-wise methods.*

(See Eklund et al. 2016)

Correction — *Describe the type of correction and how it is obtained for multiple comparisons (e.g. FWE, FDR, permutation or Monte Carlo).*

Models & analysis

n/a	Involved in the study
☐	☐ Functional and/or effective connectivity
☐	☐ Graph analysis
☐	☐ Multivariate modeling or predictive analysis

Functional and/or effective connectivity — *Report the measures of dependence used and the model details (e.g. Pearson correlation, partial correlation, mutual information).*

Graph analysis — *Report the dependent variable and connectivity measure, specifying weighted graph or binarized graph, subject- or group-level, and the global and/or node summaries used (e.g. clustering coefficient, efficiency, etc.).*

Multivariate modeling and predictive analysis — *Specify independent variables, features extraction and dimension reduction, model, training and evaluation metrics.*
